# Automated brain tumor identification using magnetic resonance imaging: A systematic review and meta-analysis

**DOI:** 10.1093/noajnl/vdac081

**Published:** 2022-05-27

**Authors:** Omar Kouli, Ahmed Hassane, Dania Badran, Tasnim Kouli, Kismet Hossain-Ibrahim, J Douglas Steele

**Affiliations:** School of Medicine, University of Dundee, Dundee, UK; NHS Greater Glasgow and Clyde, Glasgow, UK; NHS Greater Glasgow and Clyde, Glasgow, UK; NHS Greater Glasgow and Clyde, Glasgow, UK; School of Medicine, University of Dundee, Dundee, UK; Division of Neurosurgery, NHS Tayside, Dundee, UK; Division of Imaging Science and Technology, School of Medicine, University of Dundee, Dundee, UK

**Keywords:** artificial intelligence, brain tumor, machine learning, meta-analysis, segmentation

## Abstract

**Background:**

Automated brain tumor identification facilitates diagnosis and treatment planning. We evaluate the performance of traditional machine learning (TML) and deep learning (DL) in brain tumor detection and segmentation, using MRI.

**Methods:**

A systematic literature search from January 2000 to May 8, 2021 was conducted. Study quality was assessed using the Checklist for Artificial Intelligence in Medical Imaging (CLAIM). Detection meta-analysis was performed using a unified hierarchical model. Segmentation studies were evaluated using a random effects model. Sensitivity analysis was performed for externally validated studies.

**Results:**

Of 224 studies included in the systematic review, 46 segmentation and 38 detection studies were eligible for meta-analysis. In detection, DL achieved a lower false positive rate compared to TML; 0.018 (95% CI, 0.011 to 0.028) and 0.048 (0.032 to 0.072) (*P* < .001), respectively. In segmentation, DL had a higher dice similarity coefficient (DSC), particularly for tumor core (TC); 0.80 (0.77 to 0.83) and 0.63 (0.56 to 0.71) (*P* < .001), persisting on sensitivity analysis. Both manual and automated whole tumor (WT) segmentation had “good” (DSC ≥ 0.70) performance. Manual TC segmentation was superior to automated; 0.78 (0.69 to 0.86) and 0.64 (0.53 to 0.74) (*P* = .014), respectively. Only 30% of studies reported external validation.

**Conclusions:**

The comparable performance of automated to manual WT segmentation supports its integration into clinical practice. However, manual outperformance for sub-compartmental segmentation highlights the need for further development of automated methods in this area. Compared to TML, DL provided superior performance for detection and sub-compartmental segmentation. Improvements in the quality and design of studies, including external validation, are required for the interpretability and generalizability of automated models.

**Key Points**

- Human expertise outperformed automated methods in sub-compartmental segmentation.
- DL performed superiorly to TML for detection and sub-compartmental segmentation.
- Transparency and generalizability of models should be improved.

**Importance of the Study**

Despite the increasing research on artificial intelligence techniques in medical imaging, their safe implementation into clinical practice depends on rigorous and generalizable evidence. This study systematically evaluated the performance of automated brain tumor detection and segmentation methods, and assessed the quality of reporting using the Checklist for Artificial Intelligence in Medical Imaging guideline. Although automated and manual methods in whole tumor segmentation performed comparably, manual methods performed better in sub-compartmental segmentation. Within automated methods, deep learning was found to be superior to traditional machine learning in detection and sub-compartmental segmentation, but explaining this was hindered by the paucity in reported methods of model interpretability. Less than a third of studies reported external validation of their automated method. The variability found in study reporting undermines the credibility of automated methods, impacting their benefit for patients and health systems. Hence, there is a need for adherence to international reporting standards and guidelines.

Brain tumors present a significant burden on healthcare worldwide due to the neurological deficits produced and subsequent poor prognosis, with an average 5-year survival of 35% in malignant subtypes.^1^ MRI is the gold standard modality engendering brain tumor diagnosis and subsequently informing surgical intervention, radiotherapy planning, and chemotherapy. Inevitably, qualitative MRI assessment has always been subject to high inter-rater variability, as well as being a notoriously laborious process.^2^ However, the emergence of Artificial Intelligence (AI) has sparked the hope of overcoming these limitations.

The advent of Computer-Aided Diagnosis (CAD) using AI can potentially improve brain tumor patient outcomes. Traditional machine learning (TML) techniques have become widely used for image classification but are restricted by a requirement for specifying “feature vectors” for extraction from the raw data.^3^ Conversely, deep learning (DL) techniques provide effective and automatic representation of complex image features, which has contributed to their increased popularity,^3^ but the interpretation of automatically identified features remains a problem.^4^ In addition, both TML and DL techniques are vulnerable to overfitting and selection bias.^5^ Therefore, to safely use CAD in clinical settings, large robust studies which evaluate their quality and generalizability are crucial.^4^ Holistic and standardized evaluation of scientific reporting is facilitated by established guidelines, such as the recently proposed Checklist for Artificial Intelligence in Medical Imaging (CLAIM).^6^

The research on AI in neuro-oncology imaging has been amplified by the introduction of open access image datasets, such as the annual Multimodal Brain Tumor Segmentation Challenge (BRATS).^7^ This provides the ideal foundation for an in-depth review to identify optimal automated methods. Three former systematic reviews and meta-analyses evaluated performance of AI-related techniques in neuro-oncological imaging.^8–10^ However, these focused on specific brain tumor types and whole tumor (WT) segmentation, and none have evaluated sub-compartmental segmentation nor addressed performance disparities between CAD and human expert segmentation. Moreover, there remains a paucity in comprehensively assessing the quality of studies in this field. We present the largest systematic review and meta-analysis that objectively evaluates performance of automated detection and segmentation techniques and assesses the reporting quality of included studies.

## Materials and Methods

### Search Strategy

This systematic review and meta-analysis were conducted in accordance with the Preferred Reporting Items for Systematic Reviews and Meta-Analyses statement^11^ (PROSPERO; CRD42021247925). We searched PubMed, Web of Science, and Scopus for studies published between January 1, 2000, and May 8, 2021. The search was initially performed on June 19, 2020 and updated on May 8, 2021. The search strategy is found in the Supplementary Appendix. The search was limited to publications written in English. The citations of included articles were hand-searched to identify additional appropriate articles.

### Inclusion and Exclusion Criteria

Studies were included if they developed or validated a semi-automatic or fully automatic adult brain tumor detection or segmentation method using MRI. Exclusion criteria: (1) studies reporting tumor classification or tumor grading methods only; (2) studies utilizing MRI spectroscopy only for method development; (3) studies reporting methods on pediatric, pituitary, and/or brainstem tumors only; (4) abstracts or conference proceedings; and (5) no performance metrics reported.

### Study Selection and Data Extraction

Extracted citations were imported into the Rayyan systematic review site (https://www.rayyan.ai) for study selection. Following removal of duplicates, titles and abstracts were screened, and full texts of relevant publications reviewed. Study screening was completed by two independent reviewers (O.K., J.D.S.), with disagreements resolved through a consensus-based approach with the wider group.

Two independent reviewers (O.K., A.H.) extracted study characteristics from included studies with disagreements resolved through consensus. Data extracted included: (1) Author; (2) year; (3) dataset(s) utilized with the number of patients/images; (4) type of tumors(s) studied; (5) MRI modality; (6) performance evaluation metrics; (7) type of algorithm utilized; (8) feature extraction; (9) inference time in slice/second for segmentation; (10) user interaction (ie, automatic vs semi-automatic); and (11) validation technique(s).

### Reporting and Quality Evaluation

The reporting quality of studies was assessed according to CLAIM.^6^ The risk of bias and applicability was assessed using the Quality Assessment of Diagnostic Accuracy Studies 2 (QUADAS-2) guideline,^12^ with consideration of some CLAIM items (see Supplementary Appendix). Three reviewers (O.K., A.H., D.B.) independently appraised included studies with any disagreements resolved through consensus. A “good” domain was deemed by its reporting in ≥70% of studies.

### Definitions

DL was referred to studies that utilized deep neural networks as their method of choice. TML was referred to methods not classified as DL. *Detection studies* were defined as those that reported performance results for techniques that identified the presence of a tumor in an image. *Segmentation studies* were defined as those that reported performance results for techniques that segmented brain tumors, whether it was WT, tumor core (TC), and/or enhancing tumor (ET) segmentations as defined by BRATS.^7^ Following previous work, dice similarity coefficient (DSC) of ≥0.7 was considered to represent “good” overlap.^13^

### Statistical Analysis

A meta-analysis was conducted for both automated detection and segmentation studies to compare DL with TML methods and to evaluate the segmentation performance of CAD to that of manual experts. Studies providing performance metrics for their method on different datasets were assumed to be independent of each other. This is because we are interested in providing an overview of the two methods rather than exact point estimates.

For detection methods, contingency tables consisting of True Positive, False Positive, False Negative, and True Negative were constructed. For studies that did not directly provide contingency tables, missing data were calculated with Review Manager 5.3 (https://revman.cochrane.org/) using sensitivity, specificity, and number of images. If neither contingency tables nor sufficient data were reported for computation, then the study was excluded from meta-analysis. A unified hierarchical summary receiver operating characteristic model was developed for the detection meta-analysis. Summary estimates of sensitivity and specificity with 95% CIs were derived using the random-effects bivariate binomial model parameters and equivalence equations of Harbord et al.^14^ The reason for using the hierarchical model is that it considers the correlation between sensitivity and specificity, accounting for within-study variability, as well as variability (also called heterogeneity) in effects between studies (ie, between-study variability). Receiver operating characteristic (ROC) curves were used to plot summary estimates of sensitivity against false positive rate (FPR, ie, 1-specificity). The ROC curve plots also exhibit the uncertainty around the summary estimates via 95% confidence regions, and heterogeneity between accuracy estimates via 95% prediction regions.

Segmentation methods were evaluated using a random effects model, and reported in terms of pooled DSC, a universally used and reported metric. The restricted maximum likelihood estimator was used to calculate the heterogeneity variance (τ ^2^). The inverse variance method was used to calculate a pooled effect size. Knapp-Hartung adjustments were used to calculate the confidence interval. A prerequisite for study inclusion in the meta-analysis was reporting outcome of interest (ie, DSC), in combination with an SD. Subgroup analysis comparing tumor types was performed where possible. A comparative analysis was conducted to evaluate the performance of CAD versus human experts. Sensitivity analysis was performed looking at studies that only performed out-of-sample external validation. Subgroup or sensitivity analysis was avoided when the number of studies in a group is small (*n* < 5). Study heterogeneity was formally evaluated using Higgins’ inconsistency index (I^2^) (I^2^ > 50% = significant heterogeneity). All analyses were performed in R (version 4.0.2, http://www.r-project.org/) using the *tidyverse*, *metaDTA*, *dmetar*, *meta*, and *ComplexUpset* packages.

## Results

**Figure 1.**
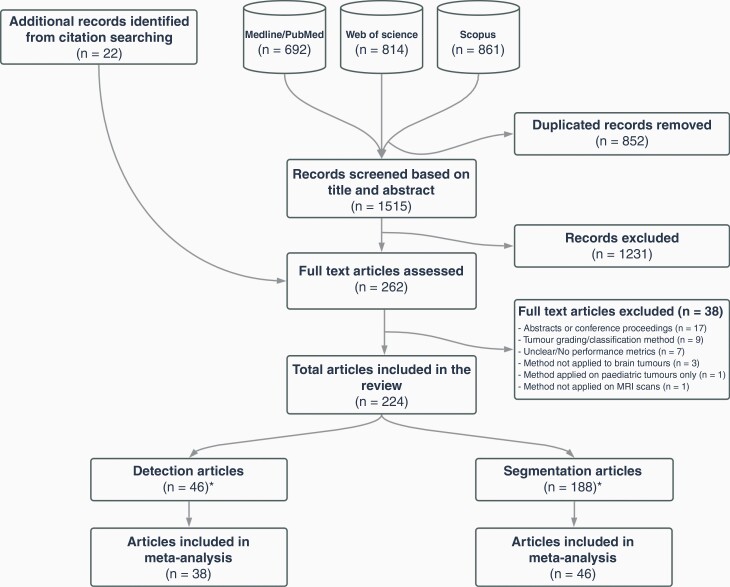
Study selection flow diagram (*10 studies reported both detection and segmentation results).

Our search identified 2367 records, of which 1515 records were screened (Figure 1). An additional 22 texts were identified through cross-referencing. Two-hundred and sixty-two full texts were assessed for eligibility and 224 were included in the systematic review: 188 segmentation and 46 detection studies (10 studies reported both detection and segmentation results; see “Eligible Studies” in Supplementary Appendix). Forty-six segmentation^15–60^ and 38 detection^39,42,45,61–95^studies were eligible for meta-analysis.

### Study Characteristics

Study characteristics are shown in Supplementary Table 1 (segmentation) and Supplementary Table 2 (detection). 40.6% (*n* =95) of studies used DL and 59.4% (*n* = 139) used TML methods. There was a clear increase in the use of DL from 2018 (Supplementary Figure 1). Most studies utilized a fully automated algorithm (*n* = 222; 94.9%).

80.7% (*n* = 189) used data from open-access repositories, with BRATS being the most popular of them (*n* = 156; 66.7%). 29.0% (*n* = 68) used local datasets, all of which were retrospectively collected data. 11.9% (*n* = 28) used both local and public datasets. 2.1% (*n* = 5) did not specify dataset(s) used (Supplementary Figure 2). Publicly available datasets are detailed in Supplementary Table 3.

The most studied tumors were high-grade gliomas (HGG) (*n* = 173; 73.9%) and low-grade gliomas (LGG) (*n* = 171; 73.1%), with 59.0% (*n* = 138) of studies involving both (Supplementary Figure 3). 9.8% (*n* = 23) did not report the type of tumor studied. Regarding MRI sequences, T2 (*n* = 169; 72.2%), fluid-attenuated inversion recovery (FLAIR) (*n* = 165; 70.5%), T1-contrast enhanced (T1CE) (*n* = 164; 70.1%), and T1 (*n* = 143; 61.1%) modalities were the most studied (Supplementary Figure 4). 48.3% (*n* = 113) of studies combined all these sequences, and 20.1% (*n* = 47) used just one for the algorithm development. A small minority (*n* = 19, 8.1%) did not report the type of MRI used.

The most common metrics used for evaluating performance were DSC (*n* = 168; 71.8%) and sensitivity (*n* = 168; 71.8%) (Supplementary Figure 5). 55.1% (*n* = 129) of studies reported internal validation. 31.2% (*n* = 73) used random split validation and 32.1% (*n* = 75) used resampling methods (Supplementary Figure 6). Overall, less than a third of studies (*n* = 70; 30%) performed external validation. Specifically, 49.5% (*n* = 47/95) of DL and 16.5% (*n* = 23/139) of TML studies reported external validation (Supplementary Figure 7). Details of algorithm performance and validation techniques of studies are found in Supplementary Table 4 (segmentation) and Supplementary Table 5 (detection). Regarding segmentation inference time, DL methods performed the fastest (median: 0.2 s/MRI slice, interquartile range [IQR]: 0.1–0.9), whereas fully automated TML methods achieved a median of 2.6 s (IQR: 1.1–12.6) and semi-automated techniques achieved 48.16 s (IQR: 6.2–134.9) (*P* < .001; Kruskal-Wallis test) (Supplementary Figure 5).

## Reporting Quality

Detailed CLAIM assessment is presented in Supplementary Table 6 (segmentation) and Supplementary Table 7 (detection). With respect to “good” reported CLAIM items, 95.3% (*n* = 223) stated the source of the data (CLAIM item 7) and 86.8% (*n* = 203) clearly reported how ground truths were derived (CLAIM items 14–18). Almost all studies reported detailed model structure and initialization of parameters (CLAIM items 22–24). 83.8% (*n* = 196) clearly reported training procedures and hyperparameters in sufficient detail (CLAIM item 25) (Supplementary Figure 9).

However, only 1.3% (*n* = 3) of studies clarified missing data handling. No studies reported sample size calculations (CLAIM item 19). Less than two-thirds (*n* = 144, 61.5%) specified how data was partitioned (CLAIM item 20). Only 32.5% (*n* = 76) of studies reported uncertainty around performance metrics (CLAIM item 29). 67.1% (*n* = 157) studies reported performing internal and/or external validation (CLAIM item 32). Just 2.6% (*n* = 6) specified inclusion and exclusion flow of participants or images (CLAIM item 33) and only 6% (*n* = 14) defined demographics and clinical characteristics of cases in each partition (CLAIM item 34). Ten studies made the algorithm source code publicly available (CLAIM item 41; for available links to source codes see Supplementary Table 8).

### Risk of bias and applicability assessment

—Detailed QUADAS-2 assessment is presented in Supplementary Table 9 (segmentation) and Supplementary Table 10 (detection). In the patient selection domain of risk of bias, 21.4% (*n* = 50) studies were considered to have unclear or high risk of bias as they did not express the exclusion criteria in the utilized dataset(s). In the reference standard domain, 13.2% (*n* = 31) were deemed to have unclear or high risk of bias as they did not clearly define how the ground truth segmentation was derived. In terms of applicability, the main source of concern was in the index test domain; 31.6% (*n* = 74) had high applicability concerns as they did not validate the algorithm (Supplementary Figure 10).

### Meta-analysis

#### Detection meta-analysis

—Thirty-eight detection studies provided sufficient data to construct contingency tables (69 tables). Only one study performed an external validation. 28.9% (*n* = 11; 20 tables) of studies utilized DL methods and the remaining 71.1% (*n* = 27; 49 tables) utilized TML methods (Table 1).

**Table 1.** Detection Meta-analysis Results

Method	Author	Year	TP	FN	FP	TN	Total	Sensitivity	Specificity	Weighted Specificity	Weighted Sensitivity
DL	Çinar and Yildirim^80^	2020	147	8	0	98	253	0.948	1	5.129	4.467
DL	Devanathan and Venkatachalapathy^81^	2020	153	2	3	95	253	0.987	0.969	4.241	4.708
DL	Gurunathan and Krishnan^82^	2020	514	25	21	1752	2312	0.954	0.988	6.356	5.694
DL	Rai et al.^65^	2021	1232	141	135	2421	3929	0.897	0.947	6.417	6.582
DL	Abd-Ellah et al.^63^	2018	239	1	0	109	349	0.996	1	2.887	3.571
DL	Atici et al.^79^	2019	1082	220	110	2171	3583	0.831	0.952	6.424	6.567
DL	Kaur and Ghandi^86^	2020	20	0	0	30	50	1	1	2.003	0.991
DL	Kaur and Ghandi^86^	2020	52	0	0	22	74	1	1	1.343	1.889
DL	Kaur and Ghandi^86^	2020	140	0	0	20	160	1	1	0.877	2.958
DL	Kaur and Ghandi^86^	2020	238	12	18	238	506	0.952	0.93	5.983	6.155
DL	Thangarajan and Chokkalingam^87^	2020	159	10	7	93	269	0.941	0.93	5.501	5.835
DL	Kalaiselvi et al.^88^	2020	56	7	17	201	281	0.889	0.922	6.145	5.163
DL	Rajinikanth et al.^89^	2020	388	12	11	589	1000	0.97	0.982	6.092	5.725
DL	Rajinikanth et al.^89^	2020	387	13	9	591	1000	0.968	0.985	6.105	5.611
DL	Rajinikanth et al.^89^	2020	392	8	8	592	1000	0.98	0.987	5.948	5.45
DL	Rajinikanth et al.^89^	2020	395	5	4	596	1000	0.988	0.993	5.692	4.845
DL	Rajinikanth et al.^89^	2020	393	7	9	391	800	0.983	0.978	5.727	5.771
DL	Rajinikanth et al.^89^	2020	395	5	6	194	600	0.988	0.97	4.967	5.814
DL	Huang et al.^90^	2020	4244	52	34	6348	10678	0.988	0.995	6.36	6.373
DL	Huang et al.^90^	2020	397	6	12	480	895	0.985	0.976	5.805	5.83
TML	Jayachandran and Dhanasekaran^67^	2013	10	0	1	4	15	1	0.8	0.424	1.387
TML	Deepa and Emmanuel^72^	2018	68	2	0	11	81	0.971	1	1.517	1.927
TML	Selvapandian and Manivannan^73^	2018	47	3	3	72	125	0.94	0.96	3.22	1.897
TML	Chen et al.^91^	2021	238	9	3	54	304	0.964	0.947	2.98	2.475
TML	Edalati-rad and Mosleh^75^	2019	42	0	1	36	79	1	0.973	1.442	1.71
TML	Dahshan et al.^95^	2014	87	0	1	13	101	1	0.929	0.534	2.283
TML	Song et al.^76^	2019	242	7	1	56	306	0.972	0.982	2.853	2.346
TML	Johnpeter and Ponnuchamy^77^	2019	58	2	2	98	160	0.967	0.98	3.142	1.729
TML	Amin et al.^69^	2017	42	4	0	39	85	0.913	1	3.12	1.362
TML	Amin et al.^69^	2017	60	5	0	35	100	0.923	1	3.045	1.619
TML	Amin et al.^64^	2019	70	0	2	14	86	1	0.875	0.64	2.333
TML	Amin et al.^64^	2019	61	1	7	17	86	0.984	0.708	1.725	2.463
TML	Amin et al.^64^	2019	68	6	2	10	86	0.919	0.833	2.433	2.375
TML	Amin et al.^64^	2019	69	0	5	12	86	1	0.706	0.646	2.473
TML	Amin et al.^64^	2019	69	0	4	15	88	1	0.789	0.72	2.432
TML	Amin et al.^64^	2019	72	0	4	10	86	1	0.714	0.543	2.469
TML	Amin et al.^64^	2019	290	0	10	106	406	1	0.914	1.481	2.544
TML	Amin et al.^64^	2019	296	0	5	105	406	1	0.955	1.445	2.487
TML	Amin et al.^64^	2019	301	50	5	50	406	0.858	0.909	3.363	2.558
TML	Amin et al.^64^	2019	296	0	10	100	406	1	0.909	1.422	2.55
TML	Amin et al.^64^	2019	306	11	0	89	406	0.965	1	3.151	2.187
TML	Amin et al.^64^	2019	295	10	1	100	406	0.967	0.99	3.175	2.298
TML	Amin et al.^64^	2019	70	4	1	11	86	0.946	0.917	2.117	2.236
TML	Amin et al.^64^	2019	70	0	2	14	86	1	0.875	0.64	2.333
TML	Amin et al.^64^	2019	74	6	0	6	86	0.925	1	1.798	2.045
TML	Amin et al.^64^	2019	71	0	3	12	86	1	0.8	0.59	2.419
TML	Amin et al.^64^	2019	74	0	0	12	86	1	1	0.518	1.969
TML	Amin et al.^64^	2019	70	3	0	13	86	0.959	1	1.965	1.916
TML	Jayachandran and Dhanasekaran^66^	2012	4	1	0	5	10	0.8	1	1.719	0.482
TML	Wang et al.^83^	2020	25	0	1	24	50	1	0.96	1.271	1.525
TML	Kesav and Rajini^84^	2020	43	1	1	21	66	0.977	0.955	1.91	1.891
TML	Alam et al.^78^	2019	38	1	0	1	40	0.974	1	0.193	1.806
TML	Murali and Meena^85^	2020	182	5	0	25	212	0.973	1	2.234	2.225
TML	Arunkumar et al.^74^	2018	20	0	1	19	40	1	0.95	1.146	1.457
TML	Gupta and Khanna^62^	2017	600	0	13	488	1101	1	0.974	2.358	2.511
TML	Gupta and Khanna^62^	2017	320	0	12	269	601	1	0.957	2.221	2.489
TML	Bahadure et al.^61^	2015	128	3	4	65	200	0.977	0.942	2.835	2.371
TML	Dvorák et al.^68^	2013	63	9	9	122	203	0.875	0.931	3.453	2.246
TML	Sriramakrishnan et al.^39^	2019	4441	70	60	78	4649	0.984	0.565	3.02	2.63
TML	Patil and Hamde^92^	2021	50	0	0	44	94	1	1	1.558	1.421
TML	Patil and Hamde^92^	2021	50	0	0	44	94	1	1	1.558	1.421
TML	Anitha and Raja^70^	2017	14	1	2	83	100	0.933	0.976	3.199	0.92
TML	Anitha and Raja^70^	2017	14	1	1	59	75	0.933	0.983	3.075	0.848
TML	Kebir et al.^42^	2019	961	332	805	1630	3728	0.743	0.669	3.547	2.625
TML	Lahmiri^71^	2017	20	0	1	29	50	1	0.967	1.496	1.311
TML	Simaiya et al.^93^	2017	504	63	111	843	1521	0.889	0.884	3.536	2.598
TML	Kalaiselvi et al.^45^	2019	1683	73	154	2740	4650	0.958	0.947	3.539	2.61
TML	Kalaiselvi et al.^45^	2019	63	0	7	211	281	1	0.968	2.734	1.917
TML	Tejas P and Padma^94^	2021	74	6	0	20	100	0.925	1	2.749	1.875

DL, deep learning; FN, False Negative; FP, false Positive; TML, Traditional Machine Learning; TN, True Negative; TP, True Positive.

**Figure 2.**
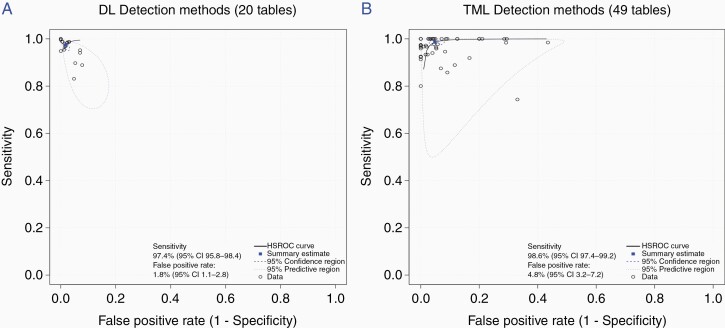
Hierarchical receiving operating curves (ROC) of (A) deep learning (DL) and (B) traditional machine learning (TML) studies included in detection meta-analysis.

Overall, the pooled sensitivity was 0.98 (95% CI, 0.97 to 0.99) and the FPR was 0.035 (95% CI, 0.025 to 0.048). DL and TML had comparable sensitivity, but DL achieved a lower FPR compared to TML; 0.018 (95% CI, 0.011 to 0.028) and 0.048 (95% CI, 0.032 to 0.072) (*P* < .001), respectively (Figures 2A and 2B).

#### Segmentation meta-analysis

—Due to limited numbers of semi-automated studies, segmentation meta-analysis solely focused on fully automated methods. Forty-six fully automated segmentation studies provided sufficient data to be included in the meta-analysis. 34.8% (*n* = 16) of studies utilized DL and 65.2% (*n* = 30) utilized TML methods. Less than half (*n* = 19; 41.3%) of studies performed external validation. 97.8% (*n* = 45) of studies provided segmentation results for WT, 41.3% (*n* = 19) for TC and 39.1% (*n* = 18) for ET.

**Figure 3.**
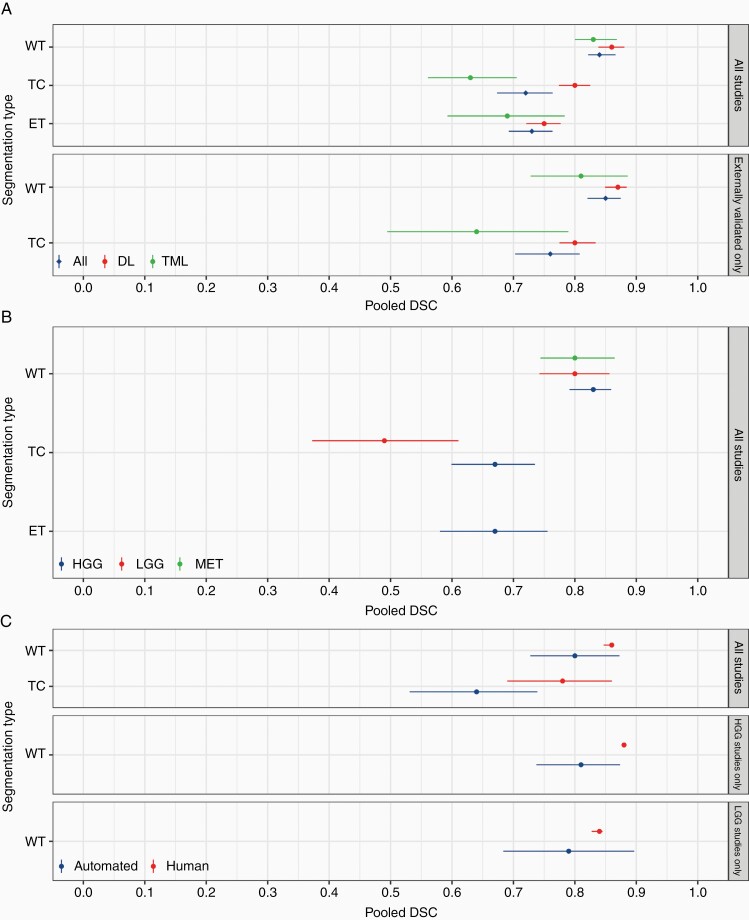
Segmentation meta-analysis for (A) all studies and externally validated only studies, stratified by deep learning (DL) and traditional machine learning (TML), (B) subgroup segmentation meta-analysis by tumor type (high-grade glioma [HGG], low-grade glioma [LGG], and metastatic brain tumor [MET]), and (C) automated versus human segmentation.

Overall, a DSC of 0.84 (95% CI, 0.82 to 0.87; I^2^ = 99.99%) for WT, 0.72 (95% CI, 0.67 to 0.76; I^2^ = 99.99%) for TC, and 0.73 (95% CI, 0.69 to 0.76; I^2^ = 99.99%) for ET (Figure 3A; Supplementary Table 11) were achieved. This persisted on sensitivity analysis of externally validated studies; a DSC of 0.85 (95% CI, 0.82 to 0.87; I^2^ = 99.97%) was achieved for WT and 0.76 (95% CI, 0.70 to 0.80; I^2^ = 99.96%) for TC (Figure 3A; Supplementary Table 12).

##### TML v*ersus* DL segmentation meta-analysis

—DL was comparable to TML for WT segmentation, 0.86 (95% CI, 0.84 to 0.88; I^2^ = 99.99%) and 0.83 (95% CI, 0.80 to 0.87; I^2^ = 99.99%; *P* = .21), respectively (Figure 3A; Supplementary Table 11). This was relatively consistent on sensitivity analysis; 0.87 (95% CI, 0.85 to 0.88; I^2^ = 100%) and 0.81 (95% CI, 0.73 to 0.89; I^2^ = 99.94%; *P* = .10), respectively (Figure 3A; Supplementary Table 12).

In terms of TC segmentation, DL achieved a statistically significant higher DSC compared to TML, 0.80 (95% CI, 0.77 to 0.83; I^2^ = 99.97%) and 0.63 (95% CI, 0.56 to 0.71; I^2^ = 100%; *P* < .001). This remained unchanged on sensitivity analysis; 0.80 (95% CI, 0.77 to 0.83; I^2^ = 99.97%) and 0.64 (95% CI, 0.49 to 0.79; I^2^ = 99.87%; *P* = .009), respectively.

Finally, for ET segmentation, DL methods achieved higher DSC when compared to TML, 0.75 (95% CI, 0.72 to 0.78; I^2^ = 99.91%) and 0.69 (95% CI, 0.59 to 0.78; I^2^ = 100%), respectively. However, this did not reach statistical significance (*P* = .17).

##### Subgroup analysis by tumor type

—Most studies (91.3%; *n* = 42/46) applied their segmentation method on gliomas (91.3%; *n* = 42/46) HGG and 84.78% (*n* = 39/46; LGG), 10.87% (*n* = 5/46) on metastatic brain tumors, 4.35% (*n* = 2/46) on meningiomas, and 1.79% (*n* = 1/46) on nerve sheath tumors. 58.69% of studies (*n* = 27/46) sufficiently categorized their segmentation results by tumor type required for subgroup analysis (Supplementary Table 13).

Since few studies applied their segmentation techniques to meningiomas and nerve sheath tumors, they could not be included in subgroup analyses. The subgroup analysis thus compared HGG, LGG, and metastatic brain tumors. Only WT segmentation results for metastatic brain tumors were possible to compute due to limited studies. ET segmentation was predominantly performed on HGG, thereby excluding it from subgroup analysis. It was not possible to compare DL and TML methods in diagnosing different types of tumors due to the small number of studies.

For WT segmentation, no difference was observed between HGG, LGG, and metastatic tumors, 0.83 (95% CI, 0.79 to 0.86; I^2^ = 99.99%), 0.80 (95% CI, 0.74 to 0.86; I^2^ = 99.98%) and 0.80 (95% CI, 0.74 to 0.86; I^2^ = 99.95%; *P* = .64), respectively (Figure 3B; Supplementary Table 13). For TC segmentation, a higher DSC was achieved for HGG compared to LGG, 0.67 (95% CI, 0.60 to 0.74; I^2^ = 99.97%) and 0.49 (95% CI, 0.37 to 0.61; I^2^ = 99.98%; *P* = .0027), respectively.

##### Automated v*ersu*s human expert segmentation

—Only 30.4% (*n* = 14/46) of studies provided sufficient data for comparison between automated and expert manual segmentation for WT and TC segmentation. All studies included multiple (>1) independent expert operators for generating ground truth segmentations; one study (7.1%) utilized two operators and 13 (92.9%) utilized four operators as part of the BRATS challenge (Supplementary Table 14).

For WT segmentation, both achieved “good” performance, but higher DSC was achieved in the manual group than the automated group 0.86 (95% CI, 0.85 to 0.86; I^2^ = 99.90%) and 0.80 (95% CI, 0.73 to 0.87; I^2^ = 99.98%; *P* = .11), respectively (Figure 3C; Supplementary Table 14). However, for TC segmentation, manual segmentation outperformed automated segmentation, 0.78 (95% CI, 0.69 to 0.86; I^2^ = 99.94%) and 0.64 (95% CI, 0.53 to 0.74; I^2^ = 99.98%; *P* = .014), respectively.

For HGG tumors, manual segmentation outperformed automated 0.88 (95% CI, 0.87 to 0.88; I^2^ = 28.85%) and 0.81 (95% CI, 0.74 to 0.87; I^2^ = 99.98%; *P* = .015), respectively. Conversely, manual was comparable to automated segmentation for LGG; 0.84 (95% CI, 0.83 to 0.85; I^2^ = 95.68%) and 0.79 (95% CI, 0.68 to 0.90, I^2^ = 99.96%, *P* = .33), respectively.

## Discussion

To date, this is the largest meta-analysis evaluating automated brain tumor segmentation and detection methods. Automation provides benefits including elimination of human inter-rater variability and reduced inference time^2^; particularly DL methods, which showed an impressive median inference time of 0.2 seconds/MRI slice.

Previous studies have concluded that, in general, automated methods are comparable to human expertise in terms of performance.^10,96^ However, our research highlights that this only holds true for WT segmentation in brain tumors. Notably, we found that manual methods outperformed automated techniques for TC segmentation. Sub-compartmental segmentation, including TC, is a major influence on tumor progression monitoring and radiotherapy planning.^97^ Hence, our finding cautions the application of machine learning in all its potential uses in routine clinical practice and highlights the need for further research on sub-compartmental automated segmentation (TC and ET). Since most methods used conventional MRI scans (ie, T1, T2, T1CE, and FLAIR), future studies could combine these multimodal sequences with other specialized MRI sequences to increase the number of features, assessing for potential enhanced segmentation results. Soltaninejad et al.^30^ and Durmo et al.^98^ incorporated features obtained from diffusion-weighted and diffusion tensor imaging and showed promising results in the automated identification of brain tumors. Including other MRI sequences in publicly available datasets, such as BRATS, could facilitate investigations into the diagnostic value of additional features.

Regarding automated detection, we have replicated the findings of Cho et al.’s systematic review on brain tumor metastasis^8^; DL had a significantly lower FPR than TML, whilst sensitivity between the two methods remained similar. To the best of our knowledge, there has been no previous evaluation of automated sub-compartmental segmentation of brain tumors. Our study extends confidence in DL to tumor segmentation; the DL group achieved “good” (DSC ≥ 0.7) performance for all segmentation types (WT, TC, ET), whereas for TML, “good” performance was limited to WT segmentation. This trend persisted with sensitivity analysis investigating only externally validated studies, reinforcing these results. DL techniques support the automatic identification of complex features unlike TML, which requires hand-crafted feature vectors.^3^ However, the advantages of DL remain ambiguous, due to its “black box” nature; the interpretability of learned features and the explainability of the model’s decisions could be improved.^3,4^ Certain methods, such as saliency maps or feature attribution attempt to deduce how these learning algorithms detect complex features.^99^ However, just 2.1% (*n* = 5) of studies reported such methods, hindering model interpretation. This highlights the importance of future work reporting DL interpretation to improve comprehension and transparency of algorithmic predictions.

Van Kempen et al.^9^ reported good performance of machine learning algorithms for glioma WT segmentation, also showing that automated segmentation for both HGG and LGG were comparable. Our subgroup analysis, stratified by tumor type, showed “good” performance, and no statistically significant difference between tumor types for WT segmentation. However, this was not consistent for TC segmentation; both HGG and LGG tumors did not reach “good” performance as was evident for WT. This is clinically pertinent, because of the aforementioned value of reliable automated sub-compartmental segmentation in treatment pathways. HGG TC segmentation performance was found to be significantly better than LGG. This may be due to LGG’s slow growth, lack of surrounding vasogenic edema, and poor enhancement on MRI, making LGGs radiologically more difficult to identify.^98^ Moreover, HGGs are highly proliferative tumors resulting in higher lesion contrast and enhancement, making them radiologically more noticeable.^98^ This study shows that although manual WT segmentation statistically outperformed automated segmentation for HGG, both achieved “good” performance (DSC ≥ 0.7). On the other hand, for LGG tumors, manual and automated segmentation were statistically comparable in terms of performance; however, only manual segmentation achieved “good” performance. This could be because LGGs can simply conform to normal anatomy (eg, expanding gyri), making them difficult to diagnose, especially when small. This further highlights the need for future work on improving machine learning performance to segment LGG more accurately to achieve comparable results to that of manual segmentation.

Reporting guidelines reinforce robust evaluation and generalizability of diagnostic models. The recent CLAIM checklist, developed on the foundations of earlier well-established guidelines, is the first to address AI applications in medical imaging. This is the first study to adopt this pertinent guideline for the comprehensive assessment of reporting quality for brain tumor identification. Although over 70% of studies detailed data sources, model design, and ground truth definitions, only a minority reported missing data handling, data partitioning, study participant flow, and external validation. This is consistent with Yusuf et al.’s systematic review^5^ which found poor reporting of the study participant flow, the distribution of disease severity, and model validation techniques within ML-based diagnosis models. Such findings reiterate the necessity for studies to employ guidelines to aid their interpretation and reusability. This is paramount in ensuring reliable research is the basis of pioneering novel techniques into clinical practice.

The absence of external validation jeopardizes the generalizability of models for clinical use. Our study highlights such a limitation, with only 41.3% (*n* = 19/46) of segmentation and 2.6% (*n* = 1/38) of detection studies in the meta-analysis undertaking external validation. To address this, we performed a sensitivity analysis on segmentation models that were externally validated, which showed similar results to the original analysis. To ensure that future studies externally validate their machine learning algorithms, authors should utilize the CLAIM guideline when reporting their study. In addition, journals should encourage authors to provide details about elements of reporting outlined CLAIM for editors and reviewers during the assessment of AI-related manuscripts in medical imaging. Secondly, high heterogeneity was observed which may be due to methodological diversity in machine learning techniques. Thirdly, only a quarter of included studies were eligible for meta-analysis because of inadequate reporting, particularly the uncertainty values of performance metrics, thus compromising data availability. This issue has been recognized by non-neuro-oncology systematic reviews.^96^ Fourthly, most studies failed to report manual segmentation results, impeding a direct comparison of the techniques. To promote standardization of ground-truth images for training AI algorithms, experts should utilize structured reporting during manual segmentation.^100^ Finally, most studies tested and trained their algorithms on open-access datasets. We propose that available automated algorithms be applied to prospective, routinely collected MRI data to assess performance and feasibility for use in daily clinical practice.

To conclude, we found promising results for the use of AI algorithms in brain tumor identification and highlight the areas for future research. Further improvements to study design are needed, with adherence to reporting guidelines, which will avail transparent evaluation and generalizability of diagnostic AI models.

## Supplementary Material

vdac081_suppl_Supplementary_Appendix
