# Supplementary material for: Automated brain tumor identification using magnetic resonance imaging: A systematic review and meta-analysis
Source: Neurooncol Adv. 2022 May 27;4(1):vdac081. doi: 10.1093/noajnl/vdac081 (PMC9234754; doi:10.1093/noajnl/vdac081)
Supplement: vdac081_suppl_Supplementary_Appendix [file vdac081_suppl_supplementary_appendix.docx]

**Supplementary material: Automated brain tumour identification using magnetic resonance imaging: a systematic review and meta-analysis**

Table of Contents

[Section 1: Search Strategy 3](#_Toc101731643)

[a) PubMed 3](#_Toc101731644)

[b) Scopus 3](#_Toc101731645)

[c) Web of Science 3](#_Toc101731646)

[Section 2: Methods 4](#_Toc101731647)

[Reporting and quality evaluation 4](#_Toc101731648)

[Section 3: Eligible studies 5](#_Toc101731649)

[Supplementary table 1 – Study characteristics of segmentation studies 11](#_Toc101731650)

[Supplementary table 2 – Study characteristics of detection studies 30](#_Toc101731651)

[Supplementary table 3 – Description of publicly available MRI brain tumour datasets 36](#_Toc101731652)

[Supplementary table 4 - Performance evaluation of segmentation studies 39](#_Toc101731653)

[Supplementary table 5 – Performance evaluation of detection studies 49](#_Toc101731654)

[Supplementary table 6: Detailed CLAIM quality reporting assessment for segmentation studies 52](#_Toc101731655)

[Supplementary table 7: Detailed CLAIM quality reporting assessment for detection studies 64](#_Toc101731656)

[Supplementary table 8: Available source codes for methods used in included studies 68](#_Toc101731657)

[Supplementary table 9: Detailed QUADAS-2 Risk of Bias and Applicability for segmentation studies 69](#_Toc101731658)

[Supplementary table 10: Detailed QUADAS-2 Risk of Bias and Applicability for detection studies 76](#_Toc101731659)

[Supplementary table 11: Segmentation meta-analysis for all studies for TML and DL methods 79](#_Toc101731660)

[Supplementary table 12: Segmentation meta-analysis for out-of-sample-externally validated studies for TML and DL methods 85](#_Toc101731661)

[Supplementary table 13: Subgroup segmentation meta-analysis by tumour type 88](#_Toc101731662)

[Supplementary table 14: Automated vs human operator segmentation meta-analysis 92](#_Toc101731663)

[Supplementary Figure 1: Cumulative number of Traditional Machine Learning (TML) and Deep Learning (DL) studies from 2000-2021 included in this review 97](#_Toc101731664)

[Supplementary Figure 2: Venn Diagram of datasets utilised in included studies 98](#_Toc101731665)

[Supplementary Figure 3: UpSet plot of interactions between the different types of tumours across included studies 99](#_Toc101731666)

[Supplementary Figure 4: UpSet plot of interactions between the different types of MRI modalities across included studies 100](#_Toc101731667)

[Supplementary Figure 5: UpSet plot of interactions between the different types of performance metrics utilised across included studies 101](#_Toc101731668)

[Supplementary Figure 6: UpSet plot of interactions between the different types of internal validation techniques utilised across included studies 102](#_Toc101731669)

[Supplementary Figure 7: Out-of-sample external validation for Traditional Machine Learning (TML) and Deep Learning (DL) of studies included 103](#_Toc101731670)

[Supplementary Figure 8: Computational (inference) time (second/MRI slice) for Traditional Machine Learning (TML) and Deep Learning (DL) segmentation for both semi-automated and fully automated techniques 105](#_Toc101731671)

[Supplementary Figure 9: Summary of CLAIM reporting quality across included studies 106](#_Toc101731672)

[Supplementary Figure 10: Summary of QUADAS-2 risk of bias and applicability assessment across included studies 107](#_Toc101731673)

[References: 108](#_Toc101731674)

# Section 1: Search Strategy

The search strategy for a) PubMed, b) Scopus, and c) Web of Science are shown. Search was limited to English language, human studies, journal articles and start date of year 2000.

## a) PubMed

((((((((((((automated) OR (automatic)) OR (fully automated)) OR (fully-automated)) OR (fully automatic)) OR (fully-automatic)) OR (semi automated)) OR (semi automatic)) OR (semi-automated)) OR (semi-automatic)) AND ((((identification) OR (detection)) OR (segmentation)) OR (diagnosis))) AND (brain tumour[MeSH Terms])) AND (MRI[MeSH Terms])

## b) Scopus

( TITLE-ABS-KEY ( automatic OR automated OR "fully automated" OR fully-automated OR "fully automatic" OR fully-automatic OR "semi automated" OR "semi automatic" OR semi-automated OR semi-automatic ) ) AND ( TITLE-ABS-KEY ( identification OR detection OR segmentation OR diagnosis ) ) AND ( TITLE-ABS-KEY ( "brain tumour" OR "brain tumor" ) ) AND ( TITLE-ABS-KEY ( mri OR "magnetic resonance imaging" OR mr OR "magnetic resonance images") )

## c) Web of Science

(((#1) AND #2) AND #3) AND #4

Query #1: TS= (automatic) OR TS= (automated) OR TS= (fully automated) OR TS= (fully-automated) OR TS= (fully automatic) OR TS= (fully-automatic) OR TS= (semi automated) OR TS= (semi automatic) OR TS= (semi-automated) OR TS= (semi-automatic)

Query #2: TS= (identification) OR TS= (detection) OR TS= (segmentation) OR TS= (diagnosis)

Query #3: TS= (brain tumour) OR TS= (brain tumor)

Query #4: TS= (mri) OR TS= (magnetic resonance imaging) OR TS= (mr) OR TS= (magnetic resonance images)

# Section 2: Methods

## Reporting and quality evaluation

The reporting quality of included studies was assessed according to the recent published Checklist for Artificial Intelligence in Medical Imaging (CLAIM) guideline.

Since there is no established method for quality assessment of artificial intelligence (AI)-related studies, the risk of bias and applicability of included studies was assessed using the Quality Assessment of Diagnostic Accuracy Studies 2 (QUADAS-2) guideline with consideration of the following CLAIM items in the following QUADAS-2 domains:

1. Risk of bias patient selection domain: Data sources and Eligibility criteria: how, where, and when potentially eligible participants or studies were identified (e.g., symptoms, results from previous tests, inclusion in registry, patient-care setting, location, dates).
2. Applicability concerns of index test: Validation or testing on external data
3. Risk of bias reference standard domain: Definition of ground truth reference standard, in sufficient detail to allow replication, Rationale for choosing the reference standard (if alternatives exist), Source of ground-truth annotations; qualifications and preparation of annotators, Annotation tools and Measurement of inter- and intrarater variability; methods to mitigate variability and/or resolve discrepancies.

Two reviewers (OK, AH) appraised all included studies using CLAIM and QUADAS-2 with any disagreements resolved through a consensus-based approach with the wider group. CLAIM items were filled with either of the following options: “Reported”, “Not reported”, “Partially Reported”, or “Not applicable”. As for the QUADAS-2 checklist, the signalling questions were filled with either “Yes”, “No” or “Unclear”. The risk of bias and applicability domains were classified as “Low” (all signalling questions were answered as “Yes”), “High” (an answer of “No” on 1 or more signalling questions) or “Unclear” (all signalling questions are answered as “Yes”, except one or more “Unclear”).

# Section 3: Eligible studies

| **Reference** | **Author** | **Year** | **Inclusion** |
| --- | --- | --- | --- |
| 1 | Fletcher-Heath et al. | 2001 | Systematic review |
| 2 | Kaus et al. | 2001 | Systematic review |
| 3 | Prastawa et al. | 2004 | Systematic review |
| 4 | Xie et al. | 2005 | Systematic review |
| 5 | Corso et al. | 2008 | Systematic review |
| 6 | Nie et al. | 2009 | Systematic review |
| 7 | Taheri et al. | 2010 | Systematic review |
| 8 | Hsieh et al. | 2011 | Systematic review |
| 9 | Hamamci et al. | 2012 | Systematic review |
| 10 | Sanjuàn et al. | 2013 | Systematic review; segmentation meta-analysis |
| 11 | Jiang et al. | 2013 | Systematic review |
| 12 | Wu et al. | 2013 | Systematic review; segmentation meta-analysis |
| 13 | Kharrat et al. | 2014 | Systematic review |
| 14 | Tustison et al. | 2014 | Systematic review |
| 15 | Wu et al. | 2014 | Systematic review |
| 16 | Demirhan et al | 2015 | Systematic review |
| 17 | Abdel-Maksoud et al. | 2015 | Systematic review |
| 18 | Ali et al. | 2015 | Systematic review |
| 19 | Njeh et al. | 2015 | Systematic review |
| 20 | Albarracín et al. | 2015 | Systematic review |
| 21 | Bahadure et al. | 2015 | Systematic review; detection meta-analysis |
| 22 | Nabizadeh and Kubat | 2015 | Systematic review |
| 23 | Dvorák et al. | 2015 | Systematic review; segmentation meta-analysis |
| 24 | Szwarc et al. | 2015 | Systematic review |
| 25 | Steed et al. | 2015 | Systematic review; segmentation meta-analysis |
| 26 | Zhan et al. | 2015 | Systematic review |
| 27 | Akkus et al. | 2015 | Systematic review |
| 28 | Hasan et al. | 2016 | Systematic review; segmentation meta-analysis |
| 29 | Mbuyamba et al. | 2016 | Systematic review; segmentation meta-analysis |
| 30 | Vishnuvarthanan et al. | 2016 | Systematic review |
| 31 | Thiruvenkadam and Perumal | 2016 | Systematic review; segmentation meta-analysis |
| 32 | Zhao and Jia | 2016 | Systematic review |
| 33 | Cordier et al. | 2016 | Systematic review |
| 34 | Pereira et al. | 2016 | Systematic review |
| 35 | Koley et al. | 2016 | Systematic review |
| 36 | Liu et al. | 2016 | Systematic review; segmentation meta-analysis |
| 37 | Li et al. | 2016 | Systematic review; segmentation meta-analysis |
| 38 | Vishnuvarthanan et al. | 2017 | Systematic review |
| 39 | Kamnitsas et al. | 2017 | Systematic review |
| 40 | Havaei et al. | 2017 | Systematic review |
| 41 | Soltaninejad et al. | 2017 | Systematic review; segmentation meta-analysis |
| 42 | Gupta and Khanna | 2017 | Systematic review; detection meta-analysis |
| 43 | Anitha and Raja | 2017 | Systematic review |
| 44 | Cui et al. | 2017 | Systematic review |
| 45 | Sasikanth and Kumar | 2017 | Systematic review |
| 46 | Imtiaz et al. | 2017 | Systematic review; segmentation meta-analysis |
| 47 | Kaur et al. | 2017 | Systematic review; segmentation meta-analysis |
| 48 | Rajinikanth et al. | 2017 | Systematic review |
| 49 | Rajinikanth et al. | 2017 | Systematic review |
| 50 | Liu et al. | 2017 | Systematic review; segmentation meta-analysis |
| 51 | Zhuge et al. | 2017 | Systematic review |
| 52 | Li et al. | 2017 | Systematic review |
| 53 | Selvapandian and Manivannan | 2018 | Systematic review |
| 54 | Raju et al. | 2018 | Systematic review |
| 55 | Essadike et al. | 2018 | Systematic review; segmentation meta-analysis |
| 56 | Kermi et al. | 2018 | Systematic review |
| 57 | Pinto et al. | 2018 | Systematic review; segmentation meta-analysis |
| 58 | Narayanan et al. | 2018 | Systematic review |
| 59 | Ma et al. | 2018 | Systematic review |
| 60 | Amin et al. | 2018 | Systematic review |
| 61 | Tong et al. | 2018 | Systematic review |
| 62 | Laukamp et al. | 2018 | Systematic review |
| 63 | Lim and Mandava | 2018 | Systematic review |
| 64 | Szilágyi et al. | 2018 | Systematic review |
| 65 | Abd-Ellah et al. | 2018 | Systematic review; detection meta-analysis |
| 66 | Angulakshmi et al. | 2018 | Systematic review |
| 67 | Perkuhn et al. | 2018 | Systematic review |
| 68 | Soltaninejad et al. | 2018 | Systematic review; segmentation meta-analysis |
| 69 | Naceur et al. | 2018 | Systematic review |
| 70 | Charron et al. | 2018 | Systematic review; segmentation meta-analysis |
| 71 | Li et al. | 2018 | Systematic review; segmentation meta-analysis |
| 72 | Zaouche et al. | 2018 | Systematic review |
| 73 | Hussain et al. | 2018 | Systematic review |
| 74 | Iqbal et al. | 2018 | Systematic review |
| 75 | Cui et al. | 2018 | Systematic review |
| 76 | Bonte et al. | 2018 | Systematic review |
| 77 | Zhan et al. | 2018 | Systematic review |
| 78 | Virupakshappa and Basavaraj | 2018 | Systematic review |
| 79 | Zhao et al. | 2018 | Systematic review |
| 80 | Zhao et al. | 2018 | Systematic review |
| 81 | Jijja and Rai | 2019 | Systematic review |
| 82 | Natarajan and Kumarasamy | 2019 | Systematic review |
| 83 | Chaudhari and Kulkarni | 2019 | Systematic review |
| 84 | Hachemi et al. | 2019 | Systematic review |
| 85 | Sheela and Suganthi | 2019 | Systematic review |
| 86 | Zhang et al. | 2019 | Systematic review |
| 87 | Grøvik et al. | 2019 | Systematic review; segmentation meta-analysis |
| 88 | Eltayeb et al. | 2019 | Systematic review; segmentation meta-analysis |
| 89 | Nagarathinam and Ponnuchamy | 2019 | Systematic review |
| 90 | Wang et al. | 2019 | Systematic review; segmentation meta-analysis |
| 91 | Li et al. | 2019 | Systematic review; segmentation meta-analysis |
| 92 | Amin et al. | 2019 | Systematic review; detection meta-analysis |
| 93 | Sun et al. | 2019 | Systematic review |
| 94 | Chang et al. | 2019 | Systematic review |
| 95 | Tong et al. | 2019 | Systematic review; segmentation meta-analysis |
| 96 | Shapey et al. | 2019 | Systematic review |
| 97 | Zhao et al. | 2019 | Systematic review |
| 98 | Dogra et al. | 2019 | Systematic review; segmentation meta-analysis |
| 99 | Hu et al. | 2019 | Systematic review |
| 100 | Na et al. | 2019 | Systematic review |
| 101 | Wang et al. | 2019 | Systematic review |
| 102 | Thaha et al. | 2019 | Systematic review |
| 103 | Razzak et al. | 2019 | Systematic review |
| 104 | Sriramakrishnan et al. | 2019 | Systematic review; detection meta-analysis; segmentation meta-analysis |
| 105 | Kumar and VijayKumar | 2019 | Systematic review |
| 106 | Mlynarski et al. | 2019 | Systematic review; segmentation meta-analysis |
| 107 | Mallick et al. | 2019 | Systematic review; segmentation meta-analysis |
| 108 | Sun et al. | 2019 | Systematic review |
| 109 | Janardhanaprabhu and Malathi | 2019 | Systematic review |
| 110 | Kebir et al. | 2019 | Systematic review; detection meta-analysis; segmentation meta-analysis |
| 111 | Meghana S et al. | 2019 | Systematic review |
| 112 | Iqbal et al. | 2019 | Systematic review |
| 113 | Alagarsamy et al. | 2019 | Systematic review; segmentation meta-analysis |
| 114 | Shivhare et al. | 2019 | Systematic review; segmentation meta-analysis |
| 115 | Nema et al. | 2019 | Systematic review |
| 116 | Peng et al. | 2019 | Systematic review |
| 117 | Kalaiselvi et al. | 2019 | Systematic review; detection meta-analysis; segmentation meta-analysis |
| 118 | Yang et al. | 2019 | Systematic review |
| 119 | Virupakshappa and Amarapur | 2019 | Systematic review |
| 120 | Wu et al. | 2019 | Systematic review; segmentation meta-analysis |
| 121 | Wang et al. | 2019 | Systematic review |
| 122 | Rehman et al. | 2019 | Systematic review; segmentation meta-analysis |
| 123 | Kharrat and Neji | 2020 | Systematic review |
| 124 | Rehman et al. | 2020 | Systematic review |
| 125 | Khosravanian et al. | 2020 | Systematic review |
| 126 | Srinivas and Rao | 2020 | Systematic review |
| 127 | Yogananda et al. | 2020 | Systematic review |
| 128 | Zhou et al. | 2020 | Systematic review; segmentation meta-analysis |
| 129 | Kumar et al. | 2020 | Systematic review |
| 130 | Chen et al. | 2020 | Systematic review |
| 131 | Chen et al. | 2020 | Systematic review |
| 132 | Tjahyaningtijas et al. | 2020 | Systematic review |
| 133 | Khan et al. | 2020 | Systematic review; segmentation meta-analysis |
| 134 | Zhang et al. | 2020 | Systematic review |
| 135 | Zhang et al. | 2020 | Systematic review |
| 136 | Xue et al. | 2020 | Systematic review; segmentation meta-analysis |
| 137 | Sun et al. | 2020 | Systematic review |
| 138 | Liu et al. | 2020 | Systematic review |
| 139 | Chaudhary et al. | 2020 | Systematic review |
| 140 | Laukamp et al. | 2020 | Systematic review |
| 141 | Thiruvenkadam and Nagarajan | 2020 | Systematic review; segmentation meta-analysis |
| 142 | Gupta et al. | 2020 | Systematic review |
| 143 | Yepuganti et al. | 2020 | Systematic review |
| 144 | Bousabarah et al. | 2020 | Systematic review |
| 145 | Ejaz et al. | 2020 | Systematic review |
| 146 | Pennig et al. | 2020 | Systematic review |
| 147 | Mohamed et al. | 2020 | Systematic review |
| 148 | Ali et al. | 2020 | Systematic review |
| 149 | Katouli and Rahmani | 2020 | Systematic review |
| 150 | Naceur et al. | 2020 | Systematic review; segmentation meta-analysis |
| 151 | Sharif et al. | 2020 | Systematic review |
| 152 | Aboelenein et al. | 2020 | Systematic review; segmentation meta-analysis |
| 153 | Zaihani et al. | 2020 | Systematic review |
| 154 | Hassen et al. | 2020 | Systematic review; segmentation meta-analysis |
| 155 | Chithra and Dheepa | 2020 | Systematic review |
| 156 | Kao et al. | 2020 | Systematic review; segmentation meta-analysis |
| 157 | Pitchai et al. | 2020 | Systematic review |
| 158 | Zeineldin et al. | 2020 | Systematic review |
| 159 | Al-qazzaz et al. | 2020 | Systematic review |
| 160 | Al-qazzaz et al. | 2020 | Systematic review |
| 161 | Sathish and Elango | 2020 | Systematic review |
| 162 | Banerjee and Mitra | 2020 | Systematic review |
| 163 | Tripathi et al. | 2020 | Systematic review |
| 164 | Kanniappan et al. | 2020 | Systematic review |
| 165 | Debnath et al. | 2020 | Systematic review; segmentation meta-analysis |
| 166 | Baid et al. | 2020 | Systematic review; segmentation meta-analysis |
| 167 | Hu et al. | 2020 | Systematic review |
| 168 | Zhou et al. | 2020 | Systematic review |
| 169 | Zhou et al. | 2020 | Systematic review |
| 170 | Barzegar and jamzad | 2020 | Systematic review |
| 171 | Gyorfi et al. | 2021 | Systematic review |
| 172 | Leva et al. | 2021 | Systematic review |
| 173 | Biratu et al. | 2021 | Systematic review |
| 174 | Rai et al. | 2021 | Systematic review; detection meta-analysis |
| 175 | Mitchell et al. | 2021 | Systematic review; segmentation meta-analysis |
| 176 | Abirami et al. | 2021 | Systematic review |
| 177 | Al-Dabagh | 2021 | Systematic review |
| 178 | Sohail et al. | 2021 | Systematic review |
| 179 | Sran et al. | 2021 | Systematic review; segmentation meta-analysis |
| 180 | Saxena et al. | 2021 | Systematic review |
| 181 | Takahashi et al. | 2021 | Systematic review; segmentation meta-analysis |
| 182 | Latif et al. | 2021 | Systematic review |
| 183 | Zhang et al. | 2021 | Systematic review |
| 184 | Lei et al. | 2021 | Systematic review |
| 185 | Wang et al. | 2021 | Systematic review |
| 186 | Zhao et al. | 2021 | Systematic review |
| 187 | Cao et al. | 2021 | Systematic review |
| 188 | Barzegar and jamzad | 2021 | Systematic review |
| 189 | Jayachandran and Dhanasekaran | 2012 | Systematic review; detection meta-analysis |
| 190 | Farjam et al. | 2012 | Systematic review |
| 191 | Jayachandran and Dhanasekaran | 2013 | Systematic review; detection meta-analysis |
| 192 | Dvorák et al. | 2013 | Systematic review; detection meta-analysis |
| 193 | Dahshan et al. | 2014 | Systematic review; detection meta-analysis |
| 194 | Helen and Kamaraj | 2015 | Systematic review |
| 195 | Thirumurugan et al. | 2016 | Systematic review |
| 196 | Banerjee et al. | 2016 | Systematic review |
| 197 | Amin et al. | 2017 | Systematic review; detection meta-analysis |
| 198 | Anitha and Raja | 2017 | Systematic review; detection meta-analysis |
| 199 | Lahmiri | 2017 | Systematic review; detection meta-analysis |
| 200 | Deepa and Emmanuel | 2018 | Systematic review; detection meta-analysis |
| 201 | Selvapandian and Manivannan | 2018 | Systematic review; detection meta-analysis |
| 202 | Arunkumar et al. | 2018 | Systematic review; detection meta-analysis |
| 203 | Edalati-rad and Mosleh | 2019 | Systematic review; detection meta-analysis |
| 204 | Song et al. | 2019 | Systematic review; detection meta-analysis |
| 205 | Johnpeter and Ponnuchamy | 2019 | Systematic review; detection meta-analysis |
| 206 | Alam et al. | 2019 | Systematic review; detection meta-analysis |
| 207 | Atici et al. | 2019 | Systematic review; detection meta-analysis |
| 208 | Çinar and Yildirim | 2020 | Systematic review; detection meta-analysis |
| 209 | Devanathan and Venkatachalapathy | 2020 | Systematic review; detection meta-analysis |
| 210 | Dikici et al. | 2020 | Systematic review |
| 211 | Dheepa and Chithra | 2020 | Systematic review |
| 212 | Gurunathan and Krishnan | 2020 | Systematic review; detection meta-analysis |
| 213 | Wang et al. | 2020 | Systematic review; detection meta-analysis |
| 214 | Kesav and Rajini | 2020 | Systematic review; detection meta-analysis |
| 215 | Murali and Meena | 2020 | Systematic review; detection meta-analysis |
| 216 | Kaur and Ghandi | 2020 | Systematic review; detection meta-analysis |
| 217 | Thangarajan and Chokkalingam | 2020 | Systematic review; detection meta-analysis |
| 218 | Kalaiselvi et al. | 2020 | Systematic review; detection meta-analysis |
| 219 | Rajinikanth et al. | 2020 | Systematic review; detection meta-analysis |
| 220 | Huang et al. | 2020 | Systematic review; detection meta-analysis |
| 221 | Chen et al. | 2021 | Systematic review; detection meta-analysis |
| 222 | Patil and hamde | 2021 | Systematic review; detection meta-analysis |
| 223 | Simaiya et al. | 2021 | Systematic review; detection meta-analysis |
| 224 | Tejas P and Padma | 2021 | Systematic review; detection meta-analysis |

# Supplementary table 1 – Study characteristics of segmentation studies

| **Author** | **Year** | **Group** | **Tumour Type** | **MRI Type** | **User interaction** | **DS1** | **DS2** | **DS3** | **DS4** | **Inference time (second/slice)** | **Feature extraction** | **Algorithm** |
| --- | --- | --- | --- | --- | --- | --- | --- | --- | --- | --- | --- | --- |
| Fletcher-Heath et al. | 2001 | TML | HGG,LGG | T1,T2,PD | Automatic | Local dataset (n=6, 45 images) |  |  |  |  | Intensity statistical features | Knowledge-based FCM |
| Kaus et al. | 2001 | TML | LGG,MEN | T1,T1CE | Automatic | Local dataset (n=20) |  |  |  | 180 |  | Statistical classification with atlas prior |
| Prastawa et al. | 2004 | TML | GBM | T2 | Automatic | Local dataset (n=4) |  |  |  |  |  | Knowledge-based generative model with outlier detection |
| Xie et al. | 2005 | TML | MEN,LGG | Unspecified | Semi-Automatic | Local dataset (n=10, 246 images) |  |  |  |  |  | Hybrid level set segmentation method driven by region and boundary information simultaneously |
| Corso et al. | 2008 | TML | GBM | T1,T2,T1CE,FLAIR | Automatic | Local dataset (n=20) |  |  |  | 5 |  | Generative model with Weighted aggregation |
| Nie et al. | 2009 | TML | LGG,HGG | T1,T2,T1CE,FLAIR | Automatic | Local dataset (n=15) |  |  |  |  |  | Hidden Markov random field + Expectation maximization |
| Taheri et al. | 2010 | TML | Unspecified | T1,T1CE | Semi-Automatic | Local dataset (n=16) |  |  |  |  | Intensity statistical features | Level-set using threshold-based speed function |
| Hsieh et al. | 2011 | TML | MEN | T1,T2 | Automatic | Local dataset (n=29) |  |  |  |  |  | Knowledge-based FCM + RGA |
| Hamamci et al. | 2012 | TML | LGG,HGG,MEN,MET,NST | T1CE | Semi-Automatic | NITRC (n=5) | WBA (n=10) | Local dataset (n=19) |  |  |  | Tumor-cut: Cellular automata |
| Sanjuàn et al. | 2013 | TML | LGG,HGG,MEN | T1 | Automatic | Local dataset (n=18) |  |  |  |  |  | Unified segmentation-normalization procedure of SPM + Outlier detection according to fuzzy-logic clustering method |
| Jiang et al. | 2013 | TML | LGG,HGG | T1,T2,T1CE,FLAIR | Semi-Automatic | BRATS 2012 taining set (n=23) |  |  |  |  | Gabor texture features | Graph cut + Adaboost |
| Wu et al. | 2013 | TML | HGG,LGG | T1CE | Semi-Automatic | Local dataset (n=21, 137 images) |  |  |  | 8 | GLCM + moments of the gray-level histogram | Graph-cut + KNN |
| Kharrat et al. | 2014 | TML | LGG,HGG,MET,MEN,SARCOMA | T1,T2,T1CE,PD | Automatic | WBA (n=7, 83 images) | Local dataset (n=2, 6 images) |  |  |  | WT-spatial gray level dependence matrix | SVM |
| Tustison et al. | 2014 | TML | LGG,HGG | T1,T2,T1CE,FLAIR | Automatic | BRATS 2013 test set (n=10) | BRATS 2013 leaderboard set (n=25) |  |  | 38.7 | Asymmetry and template normalization + intensity modeling and connected component geometry + neighborhood first-order statistics + brain mask coordinate systems | RF (ANTs/ANTsR package) |
| Wu et al. | 2014 | TML | GBM,LGG,HGG | T1,T2,T1CE,FLAIR | Automatic | Local dataset (n=20) | BRATS 2012 training set (n=30 real + 50 synthetic) |  |  | 23.2 | GWT based on superpixels | CRF + SVM + model-aware affinity |
| Demirhan et al | 2015 | TML | LGG,HGG | T1,T2,FLAIR | Automatic | Local dataset (n=20) |  |  |  | 1 | SWT | SOM |
| Abdel-Maksoud et al. | 2015 | TML | LGG,HGG | T1,T2,T1CE,FLAIR,PD | Automatic | DICOM Image Library (22 images) | BrainWeb (152 images) | BRATS 2012 training subset (n=30, 81 images) |  |  | Intensity statistical features | KIFCM (hybrid K-means + FCM) + Thresholding + Active Contour (Level Set Contouring) |
| Ali et al. | 2015 | TML | LGG,HGG | T1,T2,T1CE,FLAIR,PD | Automatic | BrainWeb (152 images) | BRATS 2012 training subset (n=30, 81 images) |  |  | 12 | Haar WT | FCM |
| Njeh et al. | 2015 | TML | LGG,HGG | T1,T2,T1CE,FLAIR | Automatic | BRATS 2012 test set (n=15 real and 15 simulated) |  |  |  | 0.5 |  | Graph cut distribution matching |
| Albarracín et al. | 2015 | TML | HGG | T1,T2,T1CE,FLAIR | Automatic | BRATS 2013 test subset (n=10) | BRATS 2013 Leaderboard subset (n=21) |  |  | 54 | Histograms | GMM/Gaussian Hidden Markov Random Field |
| Bahadure et al. | 2015 | TML | Unspecified | T1,T2,FLAIR,PD | Automatic | DICOM Image Library & BrainWeb & Local dataset (201 images) |  |  |  |  | Berkeley wavelet transformation | SVM |
| Nabizadeh and Kubat | 2015 | TML | HGG | T1,FLAIR | Automatic | BRATS 2013 training set (n=25) |  |  |  |  | GWT + statistical texture-based features (GLCM,GLRLM,HOG,LBP) | SVM |
| Dvorák et al. | 2015 | TML | HGG,LGG | T2,FLAIR | Automatic | BRATS 2012 training subset (n=22, 357 axial and 443 coronal images) |  |  |  |  | Global maximum of the total probabilistic map | Otsu thresholding |
| Szwarc et al. | 2015 | TML | HGG,MET,MEN | T1,T1CE,FLAIR,RCBV | Automatic | Local dataset (n=20) |  |  |  |  |  | Thresholding |
| Steed et al. | 2015 | TML | GBM | T1,T2,T1CE,FLAIR | Automatic | TCIA - TCGA (n=15) |  |  |  |  |  | Iterative probabilistic voxel labeling using KNN and GMM |
| Zhan et al. | 2015 | TML | HGG,LGG | T1,T2,T1CE,FLAIR | Automatic | BRATS 2012 (n=20) |  |  |  |  |  | Multiple Classifier System (Multinomial logistic regression + NB) |
| Akkus et al. | 2015 | TML | LGG | T2,T1CE | Semi-Automatic | Local dataset (n=30) | BRATS 2014 (n=25) |  |  | 275 |  | k-means + geodesic active contours using atlas prior information |
| Hasan et al. | 2016 | TML | HGG,LGG | T2 | Automatic | Local dataset (n=50) | BRATS 2013 training subset (n=25) |  |  | 243 | Modified GLCM | Three-Dimensional Active Contour without Edge |
| Mbuyamba et al. | 2016 | TML | HGG,LGG | T2,T1CE | Automatic | BRATS 2012 training subset (n=11, 312 image slices) |  |  |  | 15.8 | Hierarchical Centroid Shape Descriptor | Localized active contour model with background intensity compensation |
| Vishnuvarthanan et al. | 2016 | TML | HGG,PNET,MEN,MET,NST | T1,T2,FLAIR | Automatic | Local dataset (n=4, 17 images) & WBA (21 images) |  |  |  | 0.07 |  | SOM + FKM |
| Thiruvenkadam and Perumal | 2016 | TML | LGG,HGG | T1,T2,T1CE,FLAIR | Automatic | BRATS 2012 training subset (n=30) |  |  |  | 1.16 | DWT | FCM |
| Zhao and Jia | 2016 | DL | HGG,LGG | T1,T2,T1CE,FLAIR | Automatic | BRATS 2013 training subset (n=30 real and 50 synthetic) |  |  |  |  |  | Three-stream framework CNN |
| Cordier et al. | 2016 | TML | LGG,HGG | T1,T2,T1CE,FLAIR | Automatic | BRATS 2013 test set (n=10) | BRATS 2013 leaderboard set (n=25) | BRATS 2014 test set (n=100) |  | 6.2 | Intensity statistical features | Multi-atlas patch-based voting technique |
| Pereira et al. | 2016 | DL | LGG,HGG | T1,T2,T1CE,FLAIR | Automatic | BRATS 2013 test set (n=10) | BRATS 2013 leaderboard set (n=25) | BRATS 2015 test set (n=110) |  |  |  | CNN with small (3x3) filters for deeper architecture |
| Koley et al. | 2016 | TML | GBM,MET,MEN,GN | T1CE | Automatic | Local dataset (n=69, 360 images) |  |  |  |  | Histogram + Fourier descriptors + Fractal dimension + Gray-level difference statistics + Gabor filter bank + WT+ Fuzzy LBP | Rough entropy based image thresholding in granular computing paradigm + RF |
| Liu et al. | 2016 | TML | MET,HGG,LGG | T1CE | Automatic | BRATS 2012 training subset (n=21) | Local dataset (n=15) |  |  | 2.4 |  | Regional active contour technique |
| Li et al. | 2016 | TML | LGG,HGG | T1,T2,T1CE,FLAIR | Automatic | BRATS 2013 test set (n=10) | BRATS 2013 leaderboard set (n=25) |  |  | 1.16 | Intensity statistical features | MRF + Sparse representation |
| Vishnuvarthanan et al. | 2017 | TML | HGG,LGG,MEN,MET,PNET | T1,T2,T1CE,FLAIR | Semi-Automatic | Local dataset (n=10) |  |  |  | 0.79 | Intensity statistical features | Modified PSO + FCM + RGA |
| Kamnitsas et al. | 2017 | DL | LGG,HGG | T1,T2,T1CE,FLAIR | Automatic | BRATS 2015 training set (n=274) | BRATS 2015 test set (n=110) |  |  | 0.19 |  | DCNN+CRF (DeepMedic) |
| Havaei et al. | 2017 | DL | LGG,HGG | T1,T2,T1CE,FLAIR | Automatic | BRATS 2013 test subset (n=10) | BRATS 2013 leaderboard subset (n=25) |  |  | 1.16 |  | DCNN (InputCascadeCNN model) |
| Soltaninejad et al. | 2017 | TML | LGG,HGG | FLAIR | Automatic | Local dataset (n=19, 19 images) | BRATS 2012 training set (n=30) |  |  |  | Intensity statistical features + Gabor texton based features + Segmentation-based fractal texture analysis method (SFTA) | ERT + supervoxels |
| Gupta and Khanna | 2017 | TML | GBM,LGG | T2 | Automatic | Local dataset (n=55, 1100 images) | Local dataset (n=30, 600 images) |  |  | 1.52 | LBP + Tamura texture | Otsu’s thresholding + SVM |
| Anitha and Raja | 2017 | DL | LGG,HGG | T1,T2,T1CE,FLAIR | Automatic | BraTS 2013 training set (n=30) | BRATS 2013 test set (n=10) | BRATS 2013 Leaderboard set (n=25) |  |  | DWT + GLCM | CNN |
| Cui et al. | 2017 | DL | HGG | T1,T2,T1CE,FLAIR | Automatic | BRATS 2015 training subset (n=220) |  |  |  | 0.15 |  | Fully Convolutional Networks |
| Sasikanth and Kumar | 2017 | TML | HGG,LGG | T1,T2,T1CE,FLAIR | Automatic | BRATS 2015 training set (n=100) |  |  |  |  | LBP + LTP + GLCM + Law’s texture features | ANFIS based Graph cut approach |
| Imtiaz et al. | 2017 | TML | HGG | T1CE,T2,FLAIR | Automatic | BRATS 2013 training set (n=20) |  |  |  | 1.35 | Gabor + statistical (superpixel-based) | ERT |
| Kaur et al. | 2017 | TML | LGG | FLAIR | Automatic | BRATS 2012 training subset (n=33) | Local dataset (n=8) |  |  | 5 | Total entropy optimised via Mutation Based PSO | Optimized intuitionistic fuzzy tsallis entropy |
| Rajinikanth et al. | 2017 | TML | HGG,LGG | T1,T2,FLAIR | Automatic | BRATS 2012 taining set (2 images) |  |  |  |  | Geometric features | Firefly Algorithm + Tsallis entropy function |
| Rajinikanth et al. | 2017 | TML | LGG,HGG | T2,T1CE,FLAIR | Automatic | BRATS 2012 training subset (n=6, 72 images) |  |  |  |  |  | Teaching Learning based Optimization + Shannon’s entropy + level set/Active Contour |
| Liu et al. | 2017 | DL | LGG,HGG,MET | T1CE | Automatic | Local dataset (n=240) | BRATS 2015 training set (n=265) |  |  |  |  | DCNN (En-DeepMedic) |
| Zhuge et al. | 2017 | DL | HGG | T1,T2,T1CE,FLAIR | Automatic | BRATS 2013 training set (n=20) | Local dataset (n=10) |  |  | 0.17 |  | Holistically nested neural networks |
| Li et al. | 2017 | DL | LGG | FLAIR | Automatic | Local dataset (n=160) |  |  |  | 6 |  | CNN with Fully Connected CRF |
| Selvapandian and Manivannan | 2018 | TML | LGG,HGG | T1,T2,T1CE,FLAIR | Automatic | BRATS 2013 test subset (n=10) | BRATS 2013 leaderboard subset (n=25) |  |  |  | GLCM + Law’s texture features | ANFIS |
| Raju et al. | 2018 | TML | HGG,LGG | T1,T2,T1CE | Automatic | BRATS 2012 |  |  |  |  | Information theoretic measures + scattering WT + WT | Bayesian Harmony-Crow Search-based multi-SVNN |
| Essadike et al. | 2018 | TML | HGG,LGG | T1,T2,T1CE,FLAIR | Automatic | BRATS 2012 training subset (n=10) | BRATS 2013 training subset (n=10) |  |  | 0.41 |  | Active contour via optical Vander Lugt correlator |
| Kermi et al. | 2018 | TML | LGG,HGG | T2,FLAIR | Automatic | BRATS 2017 training subset (n=285) |  |  |  | 1.94 |  | Symmetry analysis using FBB + RGA + geodesic level set |
| Pinto et al. | 2018 | TML | LGG,HGG | T1,T2,T1CE,FLAIR | Automatic | BRATS 2013 test subset (n=10) | BRATS 2013 leaderboard subset (n=25) |  |  |  | Local and context features | Extremely randomized trees |
| Narayanan et al. | 2018 | TML | LGG,HGG,MEN,MET,PNET,NST | T1,T2,T1CE,FLAIR | Automatic | Local dataset & WBA & BrainWeb & BRATS 2013 (115 image slices) |  |  |  | 7.25 | Contrast limited adaptive histogram equalization | PSO + bacteria foraging optimization + modified FCM |
| Ma et al. | 2018 | TML | HGG,LGG | T1,T2,T1CE,FLAIR | Automatic | BRATS 2015 test set (n=110) |  |  |  | 1.94 | Fourier + WT + HOG + CNN | Concatenated and Connected RF + Contour Evolution With Multiscale Patch Driven Active Contour |
| Amin et al. | 2018 | DL | HGG,LGG | T1,T1CE,T2,FLAIR | Automatic | BRATS 2013 test subset (n=10) | BRATS 2014 test subset (n=100) | BRATS 2015 test subset (n=110) |  | 0.04 |  | CNN with 3 × 3 kernel size |
| Tong et al. | 2018 | TML | LGG,HGG | T1,T2,T1CE,FLAIR | Automatic | BRATS 2015 training subset (n=274) | BRATS 2015 test subset (n=110) |  |  |  | Non-linear intensity features | Kernel clustering dictionary learning |
| Laukamp et al. | 2018 | DL | MEN | T1CE,FLAIR | Automatic | Local dataset (n=56) |  |  |  |  |  | DCNN+CRF (DeepMedic) |
| Lim and Mandava | 2018 | TML | HGG,LGG | T2,T1CE,FLAIR | Semi-Automatic | BRATS 2013 training subset (n=30) |  |  |  |  | Homogeneity- and Object-feature based Random Walks | Information theoretic rough sets and Otsu thresholding |
| Szilágyi et al. | 2018 | TML | HGG,LGG | T1,T2,T1CE,FLAIR | Automatic | BRATS 2016 training subset (n=274) |  |  |  |  | Morphological operations + GWT | RF |
| Abd-Ellah et al. | 2018 | DL | LGG,HGG | T2 | Automatic | BRATS 2013 training subset (n=30, 804 images) |  |  |  |  |  | Five-layer region-based CNN |
| Angulakshmi et al. | 2018 | TML | HGG | T1,T1CE,T2,FLAIR | Automatic | BRATS 2012 training set (n=50 simulated and 30 real) |  |  |  | 0.13 | LBP | Superpixel based spectral clustering |
| Perkuhn et al. | 2018 | DL | GBM | T1,T2,T1CE,FLAIR | Automatic | Local dataset (n=62) |  |  |  |  |  | DCNN+CRF (DeepMedic) |
| Soltaninejad et al. | 2018 | TML | LGG,HGG | T2,T1CE,FLAIR,DTI | Automatic | Local dataset (n=11) | BRATS 2013 training subset (n=30) |  |  |  | Intensity statistical features + Gabor texton based features | RF + supervoxels |
| Naceur et al. | 2018 | DL | LGG,HGG | T1,T2,T1CE,FLAIR | Automatic | BRATS 2017 training subset (n=285) |  |  |  | 0.13 |  | End-to-end Incremental DCNN (2CNet, 3CNet and EnsembleNet) |
| Charron et al. | 2018 | DL | MET | T1,T1CE,FLAIR | Automatic | Local database (n=182, 412 images) |  |  |  |  |  | DCNN+CRF (DeepMedic) |
| Li et al. | 2018 | TML | HGG,LGG | T2,FLAIR | Automatic | BRATS 2015 training set (n=274) |  |  |  |  | Texture and intensity features | Spatial FCM + RGA + distance regularized level set evolution |
| Zaouche et al. | 2018 | TML | LGG | T1,T2,T1CE,FLAIR | Semi-Automatic | Local database (n=23) | BRATS 2015 training set (n=54) |  |  |  |  | Phase Gaussian Active Contour |
| Hussain et al. | 2018 | DL | LGG,HGG | T1,T2,T1CE,FLAIR | Automatic | BRATS 2013 training subset (n=30) | BRATS 2015 training subset (n=274) |  |  | 1.94 |  | DCNN (Inception linear nexus (ILinear)) |
| Iqbal et al. | 2018 | DL | LGG,HGG | T1,T2,T1CE,FLAIR | Automatic | BRATS 2015 test set (n=110) |  |  |  | 0.07 |  | CNN (IntNet) |
| Cui et al. | 2018 | DL | HGG,LGG | T1,T1CE,T2,FLAIR | Automatic | BRATS 2015 training set (n=274) |  |  |  | 1.54 |  | Cascaded DCNN (FCN with transfer learning + CNN with deeper architecture and smaller kernel) |
| Bonte et al. | 2018 | TML | HGG,LGG | T1CE,FLAIR | Automatic | BRATS 2017 training set (n=285) |  |  |  |  | GLCM + GLRLM + GLSZM + Tissue probability maps + Abnormality features + Symmetry-based features | RF |
| Zhan et al. | 2018 | TML | HGG,LGG | T1,T2,T1CE,FLAIR | Automatic | BRATS 2012 training set (n=50 simulated) | BRATS 2012 training set (n=30 real) | BRATS 2013 training set (n=30) |  |  |  | Multiple classifier collaborative training (SVM + sparse representation classification) with Superpixel-Based Spatial and Clinical Constraints |
| Virupakshappa and Basavaraj | 2018 | TML | LGG,HGG | T1,T2,T1CE,FLAIR | Automatic | BRATS 2015 test set (n=110) |  |  |  |  | GLCM + Gabor texture features + moment invariant | Modified level set + Adaptive ANN with Whale Optimization Algorithm |
| Zhao et al. | 2018 | DL | LGG,HGG | T1,T2,T1CE,FLAIR | Automatic | BRATS 2013 test set (n=10) | BRATS 2013 leaderboard set (n=25) | BRATS 2015 test set (n=110) |  | 1.55 |  | Fully CNN + CRF |
| Zhao et al. | 2018 | TML | LGG,HGG | T1,T2,T1CE,FLAIR | Automatic | BRATS 2013&2015 (n=354) | Local dataset (n=161) |  |  |  | LBP + GLRLM | CRF superpixel-based |
| Jijja and Rai | 2019 | DL | Unspecified | Unspecified | Automatic | Unspecified (400 images) |  |  |  |  |  | CNN with Water Cycle optimization algorithm |
| Natarajan and Kumarasamy | 2019 | TML | Unspecified | Unspecified | Automatic | ehealth laboratory (n=10) |  |  |  | 2.7 | GLCM using Fisher’s linear-discriminant analysis | Fuzzy logic with spiking neuron model |
| Chaudhari and Kulkarni | 2019 | TML | HGG | T1CE,FLAIR | Semi-Automatic | BRATS 2012&2013 training subsets (n=16) |  |  |  | 88.32 |  | K-means + Bayesian framework (Gaussian-weighted spatial neighbourhood served as prior information) |
| Hachemi et al. | 2019 | TML | HGG,LGG | T1,T1CE,T2,FLAIR | Automatic | BRATS 2015 (6 images) |  |  |  | 30 |  | RGA + Quasi-Monte Carlo-expectation + expectation maximization |
| Sheela and Suganthi | 2019 | TML | LGG,HGG,MEN | T1CE | Automatic | Publically-available Brain Tumor Dataset (708 meningioma images and 1426 glioma images) |  |  |  |  | Intensity statistical features | Greedy snake algorithm active contour + FCM optimization |
| Zhang et al. | 2019 | TML | LGG,HGG | FLAIR | Automatic | BRATS 2012 training set (n=20, 100 image slices) |  |  |  |  | Texture features | K- means++ and Gaussian kernel-based FCM |
| Grøvik et al. | 2019 | DL | MET | T1,T1CE,FLAIR | Automatic | Local dataset (n=51) |  |  |  | 0.2 |  | CNN (GoogLeNet) |
| Eltayeb et al. | 2019 | TML | HGG,LGG | FLAIR | Automatic | BRATS 2012 training subset (n=77) |  |  |  | 0.44 | CIELab color features | Iterated k-means algorithm with Calinski-Harabsz index |
| Nagarathinam and Ponnuchamy | 2019 | TML | HGG,LGG | T1,T2,T1CE,FLAIR | Automatic | BRATS 2015 training set (125 iages) | BrainWeb (175 images) |  |  | 0.15 | GLCM + sub-band metric features | ANFIS |
| Wang et al. | 2019 | DL | HGG,LGG | T1,T2,T1CE,FLAIR | Automatic | BRATS 2017 validation subset (n=46) | BRATS 2017 testing subset (146) | BRATS 2018 validation subset (n=66) | BRATS 2018 testing subset (n=191) |  |  | Cascaded framework with 2.5D CNN |
| Li et al. | 2019 | DL | HGG,LGG | T1,T2,T1CE,FLAIR | Automatic | BRATS 2015 training subset (n=54) | BRATS 2017 training subset (n=285) | BRATS 2015 testing subset (n=110) |  |  |  | Inception-based U-Net + up skip connection + cascaded training strategy |
| Amin et al. | 2019 | TML | LGG,HGG | T2,FLAIR | Automatic | Local dataset (86 images (37 normal and 49 tumourous) | BRATS 2013 training subset (n=30, 86 normal and tumourous images) | BRATS 2015 training subset (n=274, 406 normal and tumourous images) |  |  | LBP + GWT | SVM/NB/Ensemble/DT/KNN/RF |
| Sun et al. | 2019 | DL | HGG,LGG | T1,T2,T1CE,FLAIR | Automatic | BRATS 2015 test set (n=110) |  |  |  |  |  | CNN (DRRNet) |
| Chang et al. | 2019 | DL | HGG,LGG | T1,T2,T1CE,FLAIR | Automatic | BRATS 2013 test set (n=10) |  |  |  |  |  | Mix-pooling CNN with fully connected CRF |
| Tong et al. | 2019 | TML | HGG,LGG | FLAIR | Automatic | BRATS 2012 training subset (n=50) |  |  |  | 38 | GLCM | Kernel-clustering algorithm based on dictionary learning |
| Shapey et al. | 2019 | DL | NST | T1CE,T2 | Automatic | Local dataset (n=243) |  |  |  |  |  | 2.5D-CNN |
| Zhao et al. | 2019 | TML | HGG,LGG | T1,T2,T1CE,FLAIR | Automatic | BRATS 2015 training set (n=274) |  |  |  |  | Gradient-based features + context-sensitive features + circular context-sensitive features | RF |
| Dogra et al. | 2019 | TML | HGG,LGG | FLAIR | Automatic | BRATS (n=50) |  |  |  |  |  | Gradient Based kernel selection graph cut |
| Hu et al. | 2019 | DL | LGG,HGG | T1,T2,T1CE,FLAIR | Automatic | BRATS 2013 test set (n=10) | BRATS 2015 training set (n=274) | BRATS 2018 validation set (n=66) |  | 1.16 |  | Multi-cascaded CNN + fully connected CRF |
| Na et al. | 2019 | TML | HGG,LGG | T1,T2,T1CE,FLAIR | Automatic | BRATS 2015 training set (n=130) |  |  |  |  | Tamura texture feature | Radial Basis Function kernel of SVM + bagging random sampling |
| Wang et al. | 2019 | DL | LGG | T1,T2,T1CE,FLAIR | Automatic | BRATS 2018 |  |  |  |  |  | Nested dilation networks (NDNs) |
| Thaha et al. | 2019 | DL | LGG,HGG | T1,T2,T1CE,FLAIR | Automatic | BRATS 2015 training set (n=274) |  |  |  |  |  | Enhanced CNN + Novel BAT optimization algorithm |
| Razzak et al. | 2019 | DL | LGG,HGG | T1,T2,T1CE,FLAIR | Automatic | BRATS 2013 training set (n=30) | BRATS 2015 training set (n=274) |  |  |  |  | Cascaded two-pathway-group CNN |
| Sriramakrishnan et al. | 2019 | TML | HGG,LGG | T1CE,T2,FLAIR | Automatic | BRATS 2013 training set (n=30) | BRATS 2015 training set (n=274) |  |  | 0.6 | Probabilistic LTP | SVM + FCM |
| Kumar and VijayKumar | 2019 | TML | Unspecified | Unspecified | Automatic | Olivetti Research Lab (ORL) database |  |  |  |  | GLCM | Ensemle classification (SVM + Feed forwarded ANN + ELM) |
| Mlynarski et al. | 2019 | DL | LGG,HGG | T1,T2,T1CE,FLAIR | Automatic | BRATS 2017 validation set (n=46) |  |  |  |  |  | 3D CNN using long-range 2D context |
| Mallick et al. | 2019 | DL | GBM | T1CE,T2,FLAIR | Automatic | RIDER Neuro MRI (n=19) |  |  |  |  |  | Deep Wavelet Autoencoder Deep Neural Network (DWA-DNN) |
| Sun et al. | 2019 | TML | HGG,LGG | T1CE,T2,DWI,DTI | Semi-Automatic | Local dataset (n=5, 164 images) |  |  |  |  |  | Global potential field segmentation |
| Janardhanaprabhu and Malathi | 2019 | TML | Unspecified | Unspecified | Automatic | Unspecified |  |  |  |  | GLCM | Depth-First Search segmentation algorithm based on graph theory |
| Kebir et al. | 2019 | TML | HGG | T1 | Automatic | BRATS 2012 training subset (n=20, 3728 images) |  |  |  |  | SWT | FCM + Maxima Entropy Segmentation |
| Meghana S et al. | 2019 | DL | HGG,LGG | Unspecified | Automatic | Unspecified |  |  |  |  | Shape, texture and colour features using the median filter | CNN |
| Iqbal et al. | 2019 | DL | HGG,LGG | T1,T2,T1CE,FLAIR | Automatic | BRATS 2015 test set (n=110) |  |  |  |  |  | Ensemble of Long short term memory and CNN (ConvNet) |
| Alagarsamy et al. | 2019 | TML | HGG,LGG,PNET,MEN,MET,NST | T1,T2,T1CE,FLAIR,PD | Automatic | BRATS & LD & WBA & BrainWeb (245 images) |  |  |  | 2.7 |  | BAT based Interval Type-2 FCM |
| Shivhare et al. | 2019 | TML | HGG | T1CE,FLAIR | Automatic | BRATS 2015 training subset (n=22) |  |  |  | 15 | Convex hull and Harris feature extractor function | Region-based active contour |
| Nema et al. | 2019 | DL | HGG,LGG | T1,T1CE,T2,FLAIR | Automatic | BRATS 2015 training set (n=274) | BRATS 2017 training set (n=285) |  |  |  |  | CNN (RescueNet) |
| Peng et al. | 2019 | DL | HGG,LGG | T1,T1CE,T2,FLAIR | Automatic | BRATS 2015 test subset (n=110) |  |  |  |  |  | CNN (3D U-Net) |
| Kalaiselvi et al. | 2019 | TML | LGG,HGG,METS,MEN,SARCOMA | T1,T2,T1CE,FLAIR | Automatic | BRATS 2013 training set (n=30) |  |  |  | 1.54 | GLCM | Patch-based updated run length RGA + SVM |
| Yang et al. | 2019 | DL | HGG,LGG | T1,T2,T1CE,FLAIR | Automatic | BRATS 2015 training subset (n=274) |  |  |  |  | Small kernels two-path CNN | RF |
| Virupakshappa and Amarapur | 2019 | TML | HGG,LGG | T1,T2,T1CE,FLAIR | Automatic | BRATS 2015 test subset (n=110) |  |  |  |  |  | Initial contour Optimized Kernel Possibilistic C-Means + level-set |
| Wu et al. | 2019 | TML | HGG,LGG | T1,T2,T1CE,FLAIR | Automatic | BRATS 2017 training set (n=285) |  |  |  |  | Intensity statistical features + GLCM + GLGCM + GLCCM + Curvature feature + Otsu fractal features | Adaptive superpixel generation algorithm based on simple linear iterative clustering version with 0 parameter + SVM |
| Wang et al. | 2019 | DL | HGG,LGG | T1,T2,T1CE,FLAIR | Automatic | BRATS 2015 training subset (n=60) | BRATS 2018 training subset (n=60) |  |  |  |  | CNN (wide residual network & pyramid pool network (WRN-PPNet)) |
| Rehman et al. | 2019 | TML | LGG,HGG | FLAIR | Automatic | BRATS 2012 training subset (n=30) |  |  |  | 14.4 | Intensity-based statistical features + Gabor filter based texton map histogram + Otsu algorithm Fractal features | Superpixel-based RF |
| Kharrat and Neji | 2020 | DL | HGG,LGG | T1,T1CE,T2,FLAIR | Automatic | BRATS 2013 training subset (n=30) | BRATS 2015 training subset (n=274) |  |  | 0.1 |  | 3D-CNN |
| Rehman et al. | 2020 | DL | HGG,LGG | T1,T1CE,T2,FLAIR | Automatic | BRATS 2015 training set (n=274) | BRATS 2017 training and validation sets (n=431) | BRATS 2018 training and validation sets (n=476) |  |  | CNN (VGG19) | Feed-forward Neural Network |
| Khosravanian et al. | 2020 | TML | HGG,LGG | FLAIR | Automatic | BRATS 2017 training set (40 images) |  |  |  | 3.25 |  | Level-set method based on Superpixel fuzzy clustering + Lattice Boltzmann Method |
| Srinivas and Rao | 2020 | DL | HGG,LGG | T1,T1CE,T2,FLAIR | Automatic | BRATS 2018 training set (n=285) |  |  |  | 0.17 |  | CNN (VGG16 + U-net) |
| Yogananda et al. | 2020 | DL | HGG,LGG | T1,T1CE,T2,FLAIR | Automatic | BRATS 2018 training set (n=285) | BRATS 2017 validation set (n=46) | BRATS 2018 validation set (n=66) | Local dataset (n=52) |  |  | CNN (Dense UNet) |
| Zhou et al. | 2020 | DL | LGG,HGG | T1,T2,T1CE,FLAIR | Automatic | BRATS 2018 validation set (n=191) | BRATS 2015 test set (n=110) | BRATS 2017 validation set (n=46) |  |  |  | One-pass Multi-task Network (OM-Net) + cross-task guided attention module |
| Kumar et al. | 2020 | TML | HGG,LGG | T1,T1CE,T2,FLAIR | Automatic | BRATS (1000 images) |  |  |  |  | GLCM | Adaptive KNN + Optimal possibilistic FCM |
| Chen et al. | 2020 | TML | HGG | T1,T2,T1CE,FLAIR | Automatic | BRATS 2015 training subset (n=220) | BRATS 2018 training subset (n=210) |  |  | 1.35 | GMM based features + Intensity statistical features + Template based asymmetry features | RF integrated with Dense Conditional Random fields |
| Chen et al. | 2020 | DL | LGG,HGG | T1,T2,T1CE,FLAIR | Automatic | BRATS 2015 test subset (n=110) |  |  |  | 0.06 |  | Symmetric DCNN |
| Tjahyaningtijas et al. | 2020 | DL | GBM | T1,T1CE,T2,FLAIR | Automatic | BRATS 2018 (n=102) |  |  |  |  |  | CNN (mU-Net3) |
| Khan et al. | 2020 | DL | HGG,LGG | T1,T2,T1CE,FLAIR | Automatic | BRATS 2015 training subset (n=274) |  |  |  |  | Mean intensity + LBP + HOG + Three-pathway CNN | SVM |
| Zhang et al. | 2020 | DL | HGG,LGG | T1,T1CE,T2,FLAIR | Automatic | BRATS 2017 validation set (n=46) | BRATS 2018 validaton set (n=66) | BRATS 2019 validation set (n=125) |  |  |  | CNN (AGResU-Net) |
| Zhang et al. | 2020 | DL | HGG,LGG | T1,T1CE,T2,FLAIR | Automatic | BRATS 2017 training set (n=285) | BRATS 2018 validation set (n=66) |  |  |  |  | CNN (AResU-Net) |
| Xue et al. | 2020 | DL | MET | T1 | Automatic | Local datasets (n=1201) | Local datasets (n=231) | Local datasets (n=220) |  | 0.2 |  | Fully convolution network (BMDS net model) |
| Sun et al. | 2020 | DL | HGG,LGG | T1,T1CE,T2,FLAIR | Automatic | BRATS 2019 validation set (n=127) | BRATS 2018 validation set (n=66) |  |  |  |  | 3D deep multi-pathed fully convolutional neural network |
| Liu et al. | 2020 | DL | GBM,LGG | T1,T2,T1CE,FLAIR | Automatic | BRATS 2017 training set (n=167) |  |  |  |  |  | Intersection over union constraint 3D symmetric full CNN (IOUC-3DSFCNN) |
| Chaudhary et al. | 2020 | DL | LGG,HGG | T1,T1CE,T2,FLAIR | Automatic | BRATS 2013 training set (n=30) |  |  |  | 0.19 |  | DCNN inspired by UNet |
| Laukamp et al. | 2020 | DL | MEN | T1,T1CE,T2,FLAIR | Automatic | Local dataset (n=126) |  |  |  |  |  | DCNN+CRF (DeepMedic) |
| Thiruvenkadam and Perumal | 2016 | TML | HGG,LGG | T2,T1CE,FLAIR | Automatic | BRATS 2013 training subset (n=30) |  |  |  |  |  | Modified FCM |
| Gupta et al. | 2020 | TML | Unspecified | T1 | Automatic | Kaggle dataset (250 images) |  |  |  | 4.5 |  | K-means + FCM |
| Yepuganti et al. | 2020 | TML | Unspecified | Unspecified | Automatic | Local dataset (n=1) |  |  |  |  | DWT | Modified FCM |
| Bousabarah et al. | 2020 | DL | MET | T1CE,T2,FLAIR | Automatic | Local dataset (n=509) |  |  |  |  |  | DCNN (NetSUM) |
| Ejaz et al. | 2020 | TML | HGG,LGG | T1,T1CE,T2,FLAIR | Automatic | BRATS 2017 (n=285) |  |  |  |  | Intensity + shape + texture features | SOM Pixel Labelling + Kmeans + FCM |
| Pennig et al. | 2020 | DL | Lymphoma | T1,T1CE,T2,FLAIR | Automatic | Local dataset (n=43, 69 images) |  |  |  |  |  | DCNN+CRF (DeepMedic) |
| Mohamed et al. | 2020 | TML | HGG,LGG | T1,T1CE,T2,FLAIR | Automatic | BRATS 2013 (n=150) |  |  |  |  | GLCM + multifractal detrending moving average (superpixels) | Random walk + RF |
| Ali et al. | 2020 | DL | HGG,LGG | T1,T1CE,T2,FLAIR | Automatic | BRATS 2019 validation set (n=125) |  |  |  |  |  | Ensemble of 3D CNN and U-Net |
| Katouli and Rahmani | 2020 | TML | LGG,HGG,MEN | T1CE | Automatic | Publically-available Brain Tumor Dataset (708 meningioma images and 1426 glioma images) |  |  |  |  | Gabor texture features | Superpixel-based spectral clustering |
| Naceur et al. | 2020 | DL | LGG,HGG | T1,T2,T1CE,FLAIR | Automatic | BRATS 2018 validation set (n=66) |  |  |  | 0.08 | Selective attention using overlapping patches and multi-class weighted cross-entropy | DCNN (SparseMultiOCM/InputSparseMultiOCM/DenseMultiOCM models) |
| Sharif et al. | 2020 | TML | HGG,LGG | T1,T1CE,T2,FLAIR | Automatic | BRATS 2013 test set (n=10) | BRATS 2013 leaderboard set (n=25) | BRATS 2014 (n=100) | BRATS 2015 test set (n=110) | 0.71 | Gabor texture features | ELM |
| Aboelenein et al. | 2020 | DL | HGG,LGG | T1,T1CE,T2,FLAIR | Automatic | BRATS 2018 taining set (n=200) | BRATS 2018 test set (n=60) |  |  |  |  | CNN (HTTU-Net) |
| Zaihani et al. | 2020 | TML | HGG,LGG | T1 | Automatic | BRATS (30 images) |  |  |  |  |  | Sobel edge detection and mathematical morphological operations |
| Hassen et al. | 2020 | TML | HGG,LGG | T1CE,FLAIR,T2 | Automatic | BRATS 2017 training set (n=285) | BRATS 2019 training set (n=335) | BRATS 2013 training set (n=30) |  |  |  | Level-Set Segmentation + Population-based Artificial Bee Colony Clustering |
| Chithra and Dheepa | 2020 | DL | HGG,LGG | T1,T1CE,T2,FLAIR | Automatic | BRATS 2012 training set (n=80) | BRATS 2013 training set (n=80) | BRATS 2018 training set (n=275) |  |  |  | Di-phase midway convolution and deconvolution network |
| Kao et al. | 2020 | DL | HGG,LGG | T1,T1CE,T2,FLAIR | Automatic | BRATS 2017 validation set (n=46) | Brats 2018 validation set (n=66) |  |  | 1.16 |  | CNN (ensemble of 3D U-net and DeepMedic) |
| Pitchai et al. | 2020 | DL | HGG,LGG | T1,T1CE,T2,FLAIR | Automatic | BRATS 2017 training set (n=274) |  |  |  | 0.74 |  | CNN (2D-ConvNet) |
| Zeineldin et al. | 2020 | DL | HGG,LGG | FLAIR | Automatic | BRATS 2019 validation set (n=125) |  |  |  | 0.09 |  | CNN (DeepSeg) |
| Al-qazzaz et al. | 2020 | DL | HGG,LGG | T1CE,T2,FLAIR | Automatic | BRATS 2017 training set (n=285) |  |  |  |  | Histogram-based texture features + CNN | DT |
| Al-qazzaz et al. | 2020 | DL | HGG,LGG | T1,T2,T1CE,FLAIR | Automatic | BRATS 2017 training set (n=285) |  |  |  |  | Fully CNN SegNet | DT |
| Sathish and Elango | 2020 | TML | HGG,LGG | T1,T1CE,T2,FLAIR | Automatic | BRATS 2013 (n=65) | SimBRATS (n=50) |  |  | 6.09 | Statistical features + WT + Local Directional Pattern | Gaussian hybrid fuzzy clustering + Exponential cuckoo based Radial Basis Neural Network |
| Banerjee and Mitra | 2020 | DL | HGG,LGG | T1,T1CE,T2,FLAIR | Automatic | BRATS 2018 validation set (n=66) |  |  |  |  |  | Multi-Planar Spatial CNN + CRF |
| Tripathi et al. | 2020 | DL | LGG,HGG,MEN | FLAIR,T1CE | Automatic | Publically-available Brain Tumor Dataset (708 meningioma images and 1426 glioma images) |  |  |  |  |  | CNN (CCN-PR-Seg-net) |
| Kanniappan et al. | 2020 | TML | LGG,HGG | T2,T1CE | Semi-Automatic | Local dataset (22 images) | BRATS 2013 test subset (n=10, 2000 image slices) |  |  |  |  | Hybrid Fuzzy-Clustering Driven 3D-Modeling |
| Debnath et al. | 2020 | TML | HGG,LGG | FLAIR | Automatic | BRATS 2013 (n=35) |  |  |  | 1.5 |  | FCM + pixel based voxel mapping technique |
| Baid et al. | 2020 | DL | HGG,LGG | T1,T2,T1CE,FLAIR | Automatic | BRATS 2018 validation subset (n=66) | BRATS 2018 testing subset (n=191) | Local dataset (n=40) |  | 0.39 |  | CNN (3D U-Net) |
| Hu et al. | 2020 | DL | HGG,LGG | T1,T1CE,T2,FLAIR | Automatic | BRATS 2015 test subset (n=110) |  |  |  |  |  | CNN (Brain SegNet: 3D local refinement network) |
| Zhou et al. | 2020 | DL | HGG,LGG | T1,T1CE,T2,FLAIR | Automatic | BRATS 2013 test set (n=10) | BRATS 2015 test set (n=110) | BRATS 2018 validation set (n=66) |  | 0.05 |  | DCNN (AFPNet) + 3D CRF |
| Zhou et al. | 2020 | DL | HGG,LGG | T1,T1CE,T2,FLAIR | Automatic | BRATS 2013 test set (n=10) | BRATS 2013 Leaderboard set (n=25) | BRATS 2015 test set (n=110) | BRATS 2018 validation set (n=66) | 0.07 |  | CNN (DenseAFPNet) |
| Barzegar and jamzad | 2020 | TML | HGG,LGG | T1,T1CE,T2,FLAIR | Automatic | BRATS 2015 training set (n=274) | BRATS 2017 training set (n=285) | BRATS 2018 (n=335) |  |  | Gray-level differences | SVM + Ensemble (bagging) |
| Gyorfi et al. | 2021 | TML | HGG,LGG | T1,T2,T1CE,FLAIR | Automatic | BRATS 2015 training subset (n=274) | BRATS 2019 training subset (n=335) |  |  | 0.37 | Morphological features + gradient-based features + GWT | Ensembles of binary decision trees + RF |
| Leva et al. | 2021 | DL | HGG,LGG,MEN,MET,NST | T1,T1CE,FLAIR | Automatic | BRATS 2019 training subset (n=335) | BRATS 2019 validation subset (n=125) |  |  |  |  | CNN encoder-decoder architecture (ResNet) |
| Biratu et al. | 2021 | TML | HGG,LGG | T1,T2,T1CE,FLAIR | Automatic | BRATS 2015 training set (n=186) |  |  |  |  | Intensity statistical features | RGA |
| Rai et al. | 2021 | DL | LGG | T1CE,FLAIR | Automatic | TCIA (n=120, 3929 images) |  |  |  |  |  | DCNN (UnetResNext-50) |
| Mitchell et al. | 2021 | DL | HGG,LGG | T1CE,FLAIR | Automatic | Local dataset (n=741, 75,045 image slices) |  |  |  | 0.16 |  | DCNN+CRF (DeepMedic) |
| Abirami et al. | 2021 | DL | HGG | T1,T1CE,T2,FLAIR | Automatic | BRATS 2019 training set (n=250) |  |  |  |  |  | CNN (3D UNet) |
| Al-Dabagh | 2021 | TML | LGG,HGG,MEN | T1CE | Automatic | Publically-available Brain Tumor Dataset (n=233, 287 images) |  |  |  |  |  | FCM + seeded RGA |
| Sohail et al. | 2021 | DL | HGG,LGG | T1,T1CE,T2,FLAIR | Automatic | BRATS 2019 training set (n=335) | BRATS 2020 (n=335) |  |  | 0.19 |  | CNN (3D U-Net) |
| Sran et al. | 2021 | TML | HGG,LGG | T1CE,T2,FLAIR | Automatic | BRATS 2015 training set (n=10) |  |  |  |  | Center-surround Difference of Gaussians bandpass filtering | Saliency Based Segmentation + fuzzy thresholding |
| Saxena et al. | 2021 | TML | HGG,LGG | FLAIR | Automatic | BRATS 2017 training set (n=285) |  |  |  |  | Statistical features + GLRLM + GLCM + HOG + LBP + Cross Diagonal Texture Features + Simplified Texture Spectrum | SVM + FCM |
| Takahashi et al. | 2021 | DL | HGG,LGG | T1,T2,T1CE,FLAIR | Automatic | BRATS 2019 training subset (n=335) | Local dataset (n=544) |  |  |  |  | CNN (3D U-Net with fine tuning using ‘down2_up2 model’) |
| Latif et al. | 2021 | DL | HGG,LGG | T1,T2,T1CE,FLAIR | Automatic | BRATS 2015 training subset (n=274) | BRATS 2017 training subset (n=285) | BRATS 2019 training set (n=335) | BRATS 2019 validation subset (n=125) |  |  | CNN (multi-inception-UNET) |
| Zhang et al. | 2021 | DL | HGG,LGG | T1,T1CE,T2,FLAIR | Automatic | BRATS 2020 validation set (n=125) |  |  |  |  |  | CNN (multi-encoder net) |
| Lei et al. | 2021 | TML | HGG,LGG | T1,T1CE,T2,FLAIR | Automatic | BRATS 2017 training set (n=285) |  |  |  |  |  | Sparse constrained level set |
| Wang et al. | 2021 | DL | HGG,LGG | T1,T1CE,T2,FLAIR | Automatic | BRATS 2017 validation set (n=46) |  |  |  |  |  | C-dense CNN |
| Zhao et al. | 2021 | DL | HGG,LGG | T1,T1CE,T2,FLAIR | Automatic | BRATS 2018 taining set (n=285) |  |  |  | 0.01 |  | CNN (Recurrent Multi-Fiber Network) |
| Cao et al. | 2021 | DL | MET | T1CE | Automatic | Local dataset (n=195) |  |  |  |  |  | CNN (end-to-end asymmetric 3D-UNet architecture) |
| Barzegar and jamzad | 2021 | TML | HGG,LGG | T1,T1CE,T2,FLAIR | Automatic | BRATS 2015 training set (n=274) | BRATS 2017 training set (n=285) | BRATS 2019 training set (n=335) |  |  | Texture features | Weighted label fusion segmentation |
| MRI=Magnetic Resonance Imaging; DS=Dataset; DL=Deep Learning; TML=Traditional Machine Learning; HGG=High Grade Glioma; LGG=Low Grade Glioma; GBM=Glioblastoma multiforme; MET=Metastatic brain tumour; MEN=Meningioma; NST=Nerve Sheath Tumour; PNET=Primitive neuroectodermal tumor; PD=Proton-Density MRI; DTI=Diffusion Tensor Imaging MRI; DWI=Diffusion-weighted Imaging; T1CE=T1 Contrast Enhanced; FLAIR=Fluid-attenuated inversion recovery; ANFIS=Adaptive Neuro Fuzzy Inference System; FBB=Fast bounding box; RGA=Region Growing Algorithm; RF=Random forest; SVM=Support Vector Machine; CNN=Convolutional Neural Network; PSO=Particle swarm optimization; FCM=Fuzzy c-means; KNN=k-nearest neighbor; SOM=Self-organizing map; FKM=Fuzzy k-means; DCNN=Deep Convolutional Neural Network; NB=Naïve Bayes; DT=Decision Trees; CRF=Conditional Random Field; ANN=Artificial Neural Network; ELM=Extreme learning machine; GMM=Gaussian mixture model; ERT=Extremely randomised trees; GLCM=Gray-level co-occurrence matrix; SWT=Stationary wavelet transform; WT=Wavelet transform; HOG=Histogram of oriented gradients; LBP=Local Binary Pattern; GWT=Gabor Wavelet Transform; DWT=Discrete wavelet transform; LTP=Local ternary patterns; GLRLM=Grey-level run-length matrix | | | | | | | | | | | | |

# Supplementary table 2 – Study characteristics of detection studies

| **Author** | **Year** | **Group** | **Tumour Type** | **MRI Type** | **User interaction** | **DS1** | **DS2** | **DS3** | **DS4** | **Feature extraction** | **Algorithm** |
| --- | --- | --- | --- | --- | --- | --- | --- | --- | --- | --- | --- |
| Jayachandran and Dhanasekaran | 2012 | TML | Unspecified | T1 | Automatic | Local dataset (20 images (10 normal and 10 tumourous)) |  |  |  | Multi-Texton Histogram | SVM |
| Farjam et al. | 2012 | TML | MET | T1CE | Automatic | Local dataset (n=29, 4032 images) |  |  |  |  | 3D spherical shell-based template matching |
| Jayachandran and Dhanasekaran | 2013 | TML | Unspecified | Unspecified | Automatic | Local dataset (n=75) |  |  |  | Multi-Texton Histogram | Hybrid Kernel SVM |
| Dvorák et al. | 2013 | TML | Unspecified | T2 | Automatic | Local dataset (n=24 (11 normal and 13 with tumour), 203 images (131 normal and 72 tumourous) |  |  |  |  | SVM |
| Dahshan et al. | 2014 | TML | GBM,MEN,MET,SARCOMA | T2 | Automatic | WBA (101 images (14 normal and 87 tumourous) |  |  |  | DWT | ANN |
| Bahadure et al. | 2015 | TML | Unspecified | T1,T2,FLAIR,PD | Automatic | DICOM Image Library & BrainWeb & Local dataset (201 images (67 normal and 134 tumourous)) |  |  |  | Berkeley wavelet transformation | SVM |
| Helen and Kamaraj | 2015 | TML | Unspecified | T1,T2,T1CE | Automatic | TCIA (100 images) |  |  |  | Shape based features + spatial gray level dependence + GLCM | Fuzzy Clustering with Level Set Method + RF |
| Thirumurugan et al. | 2016 | TML | GBM,LGG,HGG | T1CE | Automatic | TCIA (n=17) | BRATS 2014 (n=300) |  |  | Non-subsampled Contourlet transform | ANFIS |
| Banerjee et al. | 2016 | TML | HGG,LGG | T2,T1CE,FLAIR | Automatic | BRATS 2012 training subset (n=80) |  |  |  | Pseudo-coloring scheme | Bottom-up visual saliency model |
| Amin et al. | 2017 | TML | LGG,HGG,MET,MEN,SARCOMA | T1,T2,PD | Automatic | Local dataset (85 images (39 normal and 46 tumourous) | WBA (100 images (35 normal and 65 tumourous) |  |  | GLCM | Support SVM with Gaussian Radial Based Function + Linear and Cubic Kernels |
| Gupta and Khanna | 2017 | TML | GBM,LGG | T2 | Automatic | Local dataset (n=55 (25 normal and 30 tumourous), 1100 images) | Local dataset (n=30 (14 normal and 16 tumourous), 600 images) |  |  | LBP + Tamura texture | Otsu’s thresholding + SVM |
| Anitha and Raja | 2017 | TML | Unspecified | Unspecified | Automatic | BRATS 2016 (75 images (60 normal and 15 tumourous)) | BrainWeb (100 images (85 normal and 15 tumourous)) |  |  | GLCM | RF |
| Lahmiri | 2017 | TML | HGG,LGG,MET,MEN,SARCOMA | T2 | Automatic | WBA (50 images (30 normal and 20 tumourous)) |  |  |  | Fractional-order Darwinian PSO with directional spectral distribution by Multi-scale analysis | SVM |
| Deepa and Emmanuel | 2018 | TML | LGG,HGG | T1,T2 | Automatic | BRATS 2015 training set (81 images (11 normal and 70 tumourous) |  |  |  | GWT | Adaptive Firefly backpropogation neural network |
| Selvapandian and Manivannan | 2018 | TML | MEN | Unspecified | Automatic | BrainWeb (125 images (75 normal and 50 tumourous)) |  |  |  | GLCM + intensity features + GLRLM | Gradient boosting machine learning classification |
| Abd-Ellah et al. | 2018 | DL | GBM | T1,T2 | Automatic | RIDER Neuro MRI (n=19, 349 images (109 normal and 240 tumourous)) |  |  |  | CNN | Error-correcting output codes SVM |
| Arunkumar et al. | 2018 | TML | Unspecified | Unspecified | Automatic | Unspecified (40 images (20 normal and 20 tumourous)) |  |  |  | HOG | ANN |
| Edalati-rad and Mosleh | 2019 | TML | GBM | Unspecified | Automatic | TCIA & WBA (79 images (37 normal and 42 tumourous) |  |  |  | Histogram Modeling by beta mixture model + learning automata | SVM |
| Song et al. | 2019 | TML | LGG,HGG | T2 | Automatic | Local dataset (n=120, 306 images (57 normal and 249 tumourous)) |  |  |  | GLCM + pyramid histogram + LBP + intensity-based features | Kernel SVM + PSO |
| Johnpeter and Ponnuchamy | 2019 | TML | LGG,HGG | T1,T2,T1CE,FLAIR | Automatic | BRATS 2015 taining subset (160 images (100 normal and 60 tumourous)) |  |  |  | Dual tree complex WT + Statistical features + LTP + GLCM | Co-active ANFIS |
| Amin et al. | 2019 | TML | LGG,HGG | T2,FLAIR | Automatic | Local dataset (86 images (37 normal and 49 tumourous) | BRATS 2013 training subset (n=30, 86 normal and tumourous images) | BRATS 2015 training subset (n=274, 406 normal and tumourous images) |  | LBP + GWT | SVM/NB/Ensemble/DT/KNN/RF |
| Alam et al. | 2019 | TML | Unspecified | Unspecified | Automatic | Unspecified (40 images) |  |  |  | Texture-based features | Template-based K means and improved FCM |
| Atici et al. | 2019 | DL | HGG | T2 | Automatic | Local dataset (n=179 (114 normal and 65 tumourous), 3580 images) |  |  |  |  | CNN |
| Sriramakrishnan et al. | 2019 | TML | HGG,LGG | T1CE,T2,FLAIR | Automatic | BRATS 2013 training set (n=30) |  |  |  | Probabilistic LTP | SVM + FCM |
| Kebir et al. | 2019 | TML | HGG | T1 | Automatic | BRATS 2012 training subset (n=20, 3728 images) |  |  |  | GMM + SWT | FCM + Maxima Entropy Segmentation |
| Kalaiselvi et al. | 2019 | TML | LGG,HGG,METS,MEN,SARCOMA | T1,T2,T1CE,FLAIR | Automatic | BRATS 2013 training set (n=30, 4650 image slices (2894 normal and 1756 tumourous)) | WBA (n=8 (2 normal and 6 tumourous), 281 image slices (218 normal and 63 tumourous)) |  |  | GLCM | Patch-based updated run length RGA + SVM |
| Çinar and Yildirim | 2020 | DL | Unspecified | Unspecified | Automatic | Kaggle dataset (253 images (98 normal and 155 tumourous)) |  |  |  |  | CNN (Resnet50) |
| Devanathan and Venkatachalapathy | 2020 | DL | Unspecified | Unspecified | Automatic | Kaggle dataset (253 images (98 normal and 155 tumourous)) |  |  |  | DWT | DCNN |
| Dikici et al. | 2020 | DL | MET | T1CE | Automatic | Local dataset (n=158, 217 images) |  |  |  |  | CNN (CropNet) |
| Dheepa and Chithra | 2020 | DL | HGG,LGG | T1,T1CE,T2,FLAIR | Automatic | BRATS 2018 training subset (n=285) |  |  |  |  | Input cascaded CNN |
| Gurunathan and Krishnan | 2020 | DL | HGG,LGG | T1,T1CE,T2,FLAIR | Automatic | BRATS 2015 (401 images (227 normal and 174 tumourous)) |  |  |  | GLCM + LBP | CNN (LeNET) |
| Wang et al. | 2020 | TML | HGG,LGG | T2 | Automatic | WBA (50 images (25 normal and 25 tumourous)) |  |  |  | Local generalized Hurst exponent texture features via two-dimensional multifractal detrended ﬂuctuation analysis | SVM |
| Kesav and Rajini | 2020 | TML | GBM,SARCOMA,MET | T2 | Automatic | WBA (66 images (22 normal and 44 tumourous)) |  |  |  | Discrete orthonormal S-transform | Adaboost + RF |
| Pennig et al. | 2020 | DL | Lymphoma | T1,T1CE,T2,FLAIR | Automatic | Local dataset (n=43, 69 images) |  |  |  |  | CNN (DeepMedic) |
| Murali and Meena | 2020 | TML | Unspecified | Unspecified | Automatic | Local dataset (n=200, 212 images (25 normal and 187 tumourous)) |  |  |  | Histogram | Adaptive thresholding + level set + ANN |
| Sathish and Elango | 2020 | TML | HGG,LGG | T1,T1CE,T2,FLAIR | Automatic | BRATS 2013 (65 images) | SimBRATS (50 images) |  |  | Mean + Entropy + WT + Local Directional Pattern | Gaussian hybrid fuzzy clustering + Exponential cuckoo based Radial Basis Neural Network |
| Kaur and Ghandi | 2020 | DL | HGG,LGG,MET,MEN,SARCOMA | T2 | Automatic | WBA (50 images (30 normal and 20 tumourous)) | WBA (74 images (22 normal and 52 tumourous)) | WBA (160 images (20 normal and 140 tumourous)) | Local dataset (n=200, 500 images (250 normal and 250 tumourous) |  | DCNN (Alexnet with transfer learning) |
| Thangarajan and Chokkalingam | 2020 | DL | Unspecified | Unspecified | Automatic | Kaggle dataset (270 images (100 normal and 170 tumourous)) |  |  |  | DWT + statistical features | Crossover Operated Rooster-based Chicken Swarm Optimization + CNN |
| Kalaiselvi et al. | 2020 | DL | LGG,HGG | T2 | Automatic | WBA (n=8 (2 normal and 6 tumourous), 281 images (218 normal and 63 tumourous)) |  |  |  |  | CNN (Five layers with stopping criteria and batch normalization) |
| Rajinikanth et al. | 2020 | DL | HGG,LGG | T1CE,T2,FLAIR | Automatic | BRATS (1000 images (400 normal and 600 tumourous)) | TCIA (800 images (400 normal and 400 tumourous)) | Local dataset (600 images (400 normal and 200 tumourous)) |  | Counterlet transform + curvelet transform + DWT | CNN (VGG19) + DT/KNN/SVM-Linear/SVM-RBF classifiers |
| Huang et al. | 2020 | DL | LGG | T2 | Automatic | Local dataset (n=770 (120 normal and 650 abnormal, 10678 images (6382 normal and 4296 tumourous)) | TCIA TCGA-LGG (n=110, 895 images (492 normal and 403 tumourous)) |  |  |  | Differential feature neural network |
| Chen et al. | 2021 | TML | Unspecified | T2 | Automatic | Local dataset (n=120, 304 images (57 normal and 247 tumourous) |  |  |  | GLCM | Extended Kalman Filter with SVM |
| Rai et al. | 2021 | DL | LGG | T1CE,FLAIR | Automatic | TCIA (n=120, 3929 images (2556 normal and 1373 tumourous)) |  |  |  |  | DCNN (UnetResNext-50) |
| Patil and hamde | 2021 | TML | HGG,LGG | T1,T1CE,T2,FLAIR | Automatic | ehealth laboratory + WBA (94 images (44 normal and 50 tumourous)) | BRATS 2015 (94 images (44 normal and 50 tumourous)) |  |  | LBP + Empirical wavelet transform | SVM |
| Simaiya et al. | 2021 | TML | Unspecified | Unspecified | Automatic | Kaggle dataset (1521 images) |  |  |  |  | Hierarchical K-means clustering with Fuzzy c and Super Rule tree (HKMFSRT) |
| Tejas P and Padma | 2021 | TML | Unspecified | Unspecified | Automatic | Kaggle dataset (100 images (20 normal and 80 tumourous)) |  |  |  |  | Watershed segmentation + level-set segmentation + k-means clustering |
| MRI=Magnetic Resonance Imaging; DS=Dataset; DL=Deep Learning; TML=Traditional Machine Learning; HGG=High Grade Glioma; LGG=Low Grade Glioma; GBM=Glioblastoma multiforme; MET=Metastatic brain tumour; MEN=Meningioma; NST=Nerve Sheath Tumour; PNET=Primitive neuroectodermal tumor; PD=Proton-Density MRI; DTI=Diffusion Tensor Imaging MRI; DWI=Diffusion-weighted Imaging; T1CE=T1 Contrast Enhanced; FLAIR=Fluid-attenuated inversion recovery; ANFIS=Adaptive Neuro Fuzzy Inference System; FBB=Fast bounding box; RGA=Region Growing Algorithm; RF=Random forest; SVM=Support Vector Machine; CNN=Convolutional Neural Network; PSO=Particle swarm optimization; FCM=Fuzzy c-means; KNN=k-nearest neighbor; SOM=Self-organizing map; FKM=Fuzzy k-means; DCNN=Deep Convolutional Neural Network; NB=Naïve Bayes; DT=Decision Trees; CRF=Conditional Random Field; ANN=Artificial Neural Network; ELM=Extreme learning machine; GMM=Gaussian mixture model; ERT=Extremely randomised trees; GLCM=Gray-level co-occurrence matrix; SWT=Stationary wavelet transform; WT=Wavelet transform; HOG=Histogram of oriented gradients; LBP=Local Binary Pattern; GWT=Gabor Wavelet Transform; DWT=Discrete wavelet transform; LTP=Local ternary patterns; GLRLM=Grey-level run-length matrix | | | | | | | | | | | |

# Supplementary table 3 – Description of publicly available MRI brain tumour datasets

| **Dataset** | **Tumour type** | **# Of MRI volumes** | | | **Modalities** | **Segmentation** | **Comment** | **Link to source** |
| --- | --- | --- | --- | --- | --- | --- | --- | --- |
|  |  | Total | Train | Validation/test |  |  |  |  |
| Brats 2012 | LGG, HGG | 110 | 80 (50 synthetic) | -/30 | T1,T2,T1CE,FLAIR | Manually by expert raters | 155 slices per volume | <http://www.imm.dtu.dk/projects/BRATS2012/> |
| Brats 2013 | LGG, HGG | 55 | 30 | -/25 (including leader board dataset) | T1,T2,T1CE,FLAIR | Manually by expert raters | 155 slices per volume | <https://www.smir.ch/BRATS/Start2013> |
| Brats 2014 | LGG, HGG | 238 | 200 | -/38 | T1,T2,T1CE,FLAIR | High ranked segmentation algorithms in BraTS12 and BraTS13 were fused and approved externally by raters | 155 slices per volume | <https://www.smir.ch/BRATS/Start2014> |
| Brats 2015 | LGG, HGG | 253 | 200 | -/53 | T1,T2,T1CE,FLAIR | High ranked segmentation algorithms in BraTS12 and BraTS13 were fused and approved externally by raters | 155 slices per volume | <https://www.smir.ch/BRATS/Start2015> |
| Brats 2016 | LGG, HGG | 391 | 200 | -/191 | T1,T2,T1CE,FLAIR | High ranked segmentation algorithms in BraTS12 and BraTS13 were fused and approved externally by raters | 155 slices per volume | <https://www.smir.ch/BRATS/Start2016> |
| Brats 2017 | LGG, HGG | 477 | 285 | 46/146 | T1,T2,T1CE,FLAIR | Manually using Brats 12-13 dataset and additional MRI images | 155 slices per volume | <https://www.med.upenn.edu/sbia/brats2017/data.html> |
| Brats 2018 | LGG, HGG | 542 | 285 | 66/191 | T1,T2,T1CE,FLAIR | Manually using Brats 12-13 dataset and additional MRI images | 155 slices per volume | <https://www.med.upenn.edu/sbia/brats2018/data.html> |
| Brats 2019 | LGG, HGG | 627 | 335 | 125/167 | T1,T2,T1CE,FLAIR | Manually by 1-4 neuroradiologists | 155 slices per volume | <https://www.med.upenn.edu/cbica/brats2019/data.html> |
| Brats 2020 | LGG, HGG | 660 | 369 | 125/166 | T1,T2,T1CE,FLAIR | Manually by 1-4 neuroradiologists | 155 slices per volume | <https://www.med.upenn.edu/cbica/brats2020/data.html> |
| BrainWeb | Simulated images | 152 | - | - | T1, T2, PD |  | 181 slices per volume | <https://brainweb.bic.mni.mcgill.ca/> |
| RIDER NEURO MRI | GBM | 19 | - | - | T1,T2,FLAIR,DTI | - | 368 images | <https://wiki.cancerimagingarchive.net/display/Public/RIDER+NEURO+MRI> |
| DICOM Image Library - BRAINIX |  | 22 | - | - | - | - | - | <https://www.osirix-viewer.com/resources/dicom-image-library/> |
| Publically-available Brain Tumor Dataset | Glioma, Meningioma, Pituitary | 3064 | - | - | T1CE | - | 1426,708,930 images respectively | <https://figshare.com/articles/dataset/brain_tumor_dataset/1512427> |
| Whole Brain Atlas (WBA) | Glioma, metastasis, sarcoma, meningioma |  | - | - | T1,T2 | - | - | <https://www.med.harvard.edu/aanlib/home.html> |
| TCGA-GBM | GBM | 262 | - | - | - | Automated state-of-the-art method (GLISTRboost) and manually revised by an expert board-certified neuroradiologist | 5412 images | <https://wiki.cancerimagingarchive.net/display/Public/TCGA-GBM> |
| TCGA-LGG | LGG | 199 | - | - | - | Automated state-of-the-art method (GLISTRboost) and manually revised by an expert board-certified neuroradiologist | 2275 images | <https://wiki.cancerimagingarchive.net/display/Public/TCGA-LGG> |
| MRI=Magnetic Resonance Imaging; HGG=High Grade Glioma; LGG=Low Grade Glioma; GBM=Glioblastoma multiforme; PD=Proton-Density MRI; DTI=Diffusion Tensor Imaging MRI; DWI=Diffusion-weighted Imaging; T1CE=T1 Contrast Enhanced; FLAIR=Fluid-attenuated inversion recovery | | | | | | | | |

# Supplementary table 4 - Performance evaluation of segmentation studies

| **Author** | **Year** | **Group** | **Internal validation** | **Out-of-sample external validation** | **Performance metrics** | **DSC** | **SEN** | **ACC** |
| --- | --- | --- | --- | --- | --- | --- | --- | --- |
| Fletcher-Heath et al. | 2001 | TML | Train/test split (31/69%) | No | CR,PM |  |  |  |
| Kaus et al. | 2001 | TML | NR | No | ACC |  |  | 99.68 |
| Prastawa et al. | 2004 | TML | NR | No | JSC,HD |  |  |  |
| Xie et al. | 2005 | TML | NR | No | CR,PM |  |  |  |
| Corso et al. | 2008 | TML | Train/test split (50/50%) + Cross-validation | No | JSC,PPV,SEN,HD |  | 82.5 |  |
| Nie et al. | 2009 | TML | NR | No | JSC |  |  |  |
| Taheri et al. | 2010 | TML | NR | No | JSC,HD |  |  |  |
| Hsieh et al. | 2011 | TML | NR | No | CR,PM |  |  |  |
| Hamamci et al. | 2012 | TML | NR | No | DSC | DS1: 0.83, DS2: 0.89, DS3: 0.8 |  |  |
| Sanjuàn et al. | 2013 | TML | NR | No | DSC,SEN,SPEC,AUC | 0.72 | 63.15 |  |
| Jiang et al. | 2013 | TML | NR | No | DSC,JSC,SEN,SPEC | 0.85 | 87.2 |  |
| Wu et al. | 2013 | TML | Train/test split (50/50%) | No | DSC | 0.94 | 0.03 |  |
| Kharrat et al. | 2014 | TML | Cross-validation | No | SEN,SPEC,ACC,HD,DSC | DS2: 0.86 | DS1: 95.6 | DS1: 99.5 |
| Tustison et al. | 2014 | TML | Leave-one-out cross-validation | Yes | DSC,SEN,PPV | DS1: 0.87, DS2: 0.79 | DS1: 89, DS2: 81 |  |
| Wu et al. | 2014 | TML | Cross-validation | No | DSC,JSC | DS2: 0.6 (TC) |  |  |
| Demirhan et al | 2015 | TML | NR | No | DSC,SEN,SPEC | 0.61 (TC) | 53.51 (TC) |  |
| Abdel-Maksoud et al. | 2015 | TML | NR | No | ACC,PPV,SEN |  | DS1: 90.5, DS2: 100, DS3: 100 | DS1: 90.5, DS2: 100, DS3: 100 |
| Ali et al. | 2015 | TML | NR | No | SI,ACC |  |  | DS1: 97.05, DS2: 95.85 |
| Njeh et al. | 2015 | TML | NR | Yes | DSC | 0.88 (simulated), 0.77 (real) |  |  |
| Albarracín et al. | 2015 | TML | NR | Yes | DSC,PPV,SEN,Kappa | DS1 (GMM): 0.69, DS1 (GHMRF): 0.72, DS2 (GMM): 0.74, DS2 (GHMRF): 0.77 | DS1 (GMM): 78, DS1 (GHMRF): 81, DS2 (GMM): 81, DS2 (GHMRF): 84 |  |
| Bahadure et al. | 2015 | TML | NR | No | DSC | 0.82 |  |  |
| Nabizadeh and Kubat | 2015 | TML | Cross-validation | No | ACC,SPEC,SEN |  |  | 93.8 (Statistical), 92.8 (Gabor) |
| Dvorák et al. | 2015 | TML | Cross-validation | Yes | DSC,ACC | 0.85 (axial), 0.82 (coronal) |  | 96 (axial), 94 (coronal) |
| Szwarc et al. | 2015 | TML | NR | No | SEN,SPEC,DSC | 0.72 | 64.84 |  |
| Steed et al. | 2015 | TML | NR | Yes | DSC | 0.92 (ET) |  |  |
| Zhan et al. | 2015 | TML | NR | No | JSC |  |  |  |
| Akkus et al. | 2015 | TML | Train/test split (50/50%) | Yes | DSC,JSC,NPV,PPV | DS1: 0.90, DS2: 0.85 |  |  |
| Hasan et al. | 2016 | TML | Cross-validation | Yes | DSC,JSC,SEN,SPEC,ACC | DS1: 0.89, DS2: 0.89 | DS1: 85.4, DS2: 90.9 | DS1: 99.9, DS2: 99.8 |
| Mbuyamba et al. | 2016 | TML | NR | No | DSC,SEN,SPEC,HD | 0.91 | 95.01 |  |
| Vishnuvarthanan et al. | 2016 | TML | NR | No | DSC,JSC,SEN,SPEC,ACC,MSE,EF | 0.47 | 87 |  |
| Thiruvenkadam and Nagarajan | 2020 | TML | NR | No | DSC,SEN,PPV,Kappa | 0.73 | 71 |  |
| Zhao and Jia | 2016 | DL | NR | No | DSC | 0.81 |  |  |
| Cordier et al. | 2016 | TML | Leave-one-out cross-validation | Yes | SEN,PPV,DSC,HD,ASSD | DS1: 0.87, DS2: 0.61, DS3: 0.84 |  |  |
| Pereira et al. | 2016 | DL | Cross-validation | Yes | DSC,SEN,PPV | DS1: 0.88, DS2: 0.84, DS3: 0.78 | DS1: 86, DS2: 85 |  |
| Koley et al. | 2016 | TML | Train/test split (66/33%) + Cross-validation | No | PPV,SEN,DSC,ACC,JSC,PM,CR | 0.97 | 97.2 | 97.2 |
| Liu et al. | 2016 | TML | NR | No | DSC,NMI,SSIM,HD,MSSD,SDSSD | DS1: 0.89, DS2: 0.86 |  |  |
| Li et al. | 2016 | TML | Leave-one-out cross-validation | Yes | DSC,PPV,SEN,Kappa | DS1: 0.85, DS2: 0.61 | DS1: 86, DS2: 85 |  |
| Vishnuvarthanan et al. | 2017 | TML | NR | No | EF,SEN,DSC,MSE,JSC | 0.97 | 97.3 |  |
| Kamnitsas et al. | 2017 | DL | Train/test split (78/22%) + Cross-validation | Yes | DSC,PPV,SEN | DS1: 0.9, DS2: 0.85 | DS1: 89.1, DS2: 87.7 |  |
| Havaei et al. | 2017 | DL | Cross-validation | Yes | DSC,SEN,SPEC | DS1: 0.88, DS2: 0.84 | DS1: 87, DS2: 84 |  |
| Soltaninejad et al. | 2017 | TML | Leave-one-out cross-validation | Yes | DSC,BER,SEN,PPV | DS1: 0.91, DS2: 0.88 | DS1: 89.48, DS2: 88.09 |  |
| Gupta and Khanna | 2017 | TML | Cross-validation | No | JSC,DSC | DS1: 0.73, DS2: 0.72 |  |  |
| Anitha and Raja | 2017 | DL | NR | No | ACC,SEN,SPEC |  | DS1: 97.4, DS2: 98.1, DS3: 96.6 | DS1: 98.7, DS2: 99.1, DS3: 98.4 |
| Cui et al. | 2017 | DL | Cross-validation | No | DSC,SEN,PPV | 0.84 |  |  |
| Sasikanth and Kumar | 2017 | TML | Train/test split (40/60%) | No | SEN,SPEC,ACC,DSC | 0.85 | 91 | 99.58 |
| Imtiaz et al. | 2017 | TML | Cross-validation | No | SEN,SPEC,PPV,JSC,DSC | 0.86 | 84 |  |
| Kaur et al. | 2017 | TML | NR | No | JSC,DSC,MCE | DS1: 0.80313, DS2: 0.94169 |  |  |
| Rajinikanth et al. | 2017 | TML | NR | Yes | JSC,DSC,SEN,SPEC,ACC | 0.91 | 97.86 | 96.83 |
| Rajinikanth et al. | 2017 | TML | NR | Yes | DSC,JSC,FPR,FNR,SEN,SPEC,ACC | 0.94 | 98.2 | 94.1 |
| Liu et al. | 2017 | DL | Cross-validation | No | DSC,AUC,MSSD,SDSSD | DS1: 0.67, DS2: 0.78 |  |  |
| Zhuge et al. | 2017 | DL | Cross-validation | Yes | DSC,SEN | DS1: 0.78, DS2: 0.83 | DS1: 81, DS2: 85 |  |
| Li et al. | 2017 | DL | Train/test split (62.5/37.5%) | No | DSC,SEN,PPV | 0.85 | 84.5 |  |
| Selvapandian and Manivannan | 2018 | TML | NR | Yes | SEN,SPEC,ACC |  | DS1: 96.2, DS2: 92.3 | DS1: 96.4, DS2: 95.9 |
| Raju et al. | 2018 | TML | Train/test split (90/10%) + Cross-validation | No | ACC,SEN,SPEC |  | 96.9 | 93 |
| Essadike et al. | 2018 | TML | NR | No | DSC,SEN,SPEC,HD | DS1: 0.97, DS2: 0.95 | DS1: 97.33, DS2: 96.60 |  |
| Kermi et al. | 2018 | TML | NR | No | SEN,FPR,HD,Kappa |  | 85.3 |  |
| Pinto et al. | 2018 | TML | Cross-validation | Yes | DSC,PPV,SEN | DS1: 0.85, DS2: 0.84 | DS1: 84, DS2: 84 |  |
| Narayanan et al. | 2018 | TML | NR | No | DSC,SI,SEN,SPEC | 0.67 | 95.5 |  |
| Ma et al. | 2018 | TML | Cross-validation | Yes | SEN,SPEC,DSC | 0.89 | 90 |  |
| Amin et al. | 2018 | DL | NR | Yes | DSC,SEN,SPEC,ACC,JSC,PPV | DS1: 0.998, DS2: 0.929, DS3: 0.95 | DS1: 99.7, DS2: 93.0, DS3: 95.0 | DS1: 99.8, DS2: 93.1, DS3: 95.1 |
| Tong et al. | 2018 | TML | Train/test split (25/75%) + Cross-validation | Yes | DSC,SEN,PPV,Kappa | DS1: 0.82, DS2: 0.83 | DS1: 78, DS2: 82 |  |
| Laukamp et al. | 2018 | DL | NR | Yes | DSC,ACC | 0.81 |  | 98 |
| Lim and Mandava | 2018 | TML | NR | No | DSC,JSC,FPR,FNR | 0.69 |  |  |
| Szilágyi et al. | 2018 | TML | NR | No | DSC,SEN,SPEC | 0.83 | 85 |  |
| Abd-Ellah et al. | 2018 | DL | Train/test split (74/26%) | No | SEN,PPV,DSC | 0.87 | 95.44 |  |
| Angulakshmi et al. | 2018 | TML | NR | No | DSC,SEN,SPEC | 0.87 (simulated), 0.72 (real) |  |  |
| Perkuhn et al. | 2018 | DL | Train/test split (86/14%) | Yes | DSC,PPV,SEN | 0.86 | 84 |  |
| Soltaninejad et al. | 2018 | TML | Cross-validation | Yes | DSC,PPV,BER,SEN | DS1: 0.84, DS2: 0.89 | DS1: 86.25, DS2: 96.1 |  |
| Naceur et al. | 2018 | DL | NR | No | DSC,SEN,SPEC,HD | 0.88 | 86.3 |  |
| Charron et al. | 2018 | DL | Train/test split (90/10%) | Yes | DSC,SEN,FPR,PPV | 0.79 | 98 |  |
| Li et al. | 2018 | TML | Train/test split (80/20%) | No | DSC,SEN,PPV,HD,ED | 0.86 | 83.67 |  |
| Zaouche et al. | 2018 | TML | NR | No | DSC,HD,MAD,ACC,SEN,SPEC,PPV | DS1: 0.91, DS2: 0.87 | DS1: 83.9, DS2: 94.1 | DS1: 99.3, DS2: 89.7 |
| Hussain et al. | 2018 | DL | Train/test split (80/20%) | No | DSC,SEN,SPEC | DS1: 0.87, DS2: 0.86 | DS1: 90, DS2: 86 |  |
| Iqbal et al. | 2018 | DL | Train/test split (87.5/12.5%) + Cross-validation | Yes | DSC,SEN,SPEC | 0.9 | 86 |  |
| Cui et al. | 2018 | DL | Train/test split (87.5/12.5%) + Cross-validation | No | DSC,PPV,SEN | 0.9 | 87 |  |
| Bonte et al. | 2018 | TML | Cross-validation | Yes | DSC | 0.71 |  |  |
| Zhan et al. | 2018 | TML | NR | No | DSC,PPV,SEN | DS1: 0.91, DS2: 0.84, DS3: 0.88 | DS3: 84 |  |
| Virupakshappa and Basavaraj | 2018 | TML | NR | Yes | DSC,ACC,SEN,SPEC,AUC | 0.54 | 96 | 98 |
| Zhao et al. | 2018 | DL | NR | Yes | DSC,SEN,PPV | DS1: 0.88, DS2: 0.86, DS3: 0.84 | DS1: 86, DS2: 85, DS3: 82 |  |
| Zhao et al. | 2018 | TML | NR | No | DSC,HD,SEN,SPEC | DS1: 0.81, DS2: 0.82 | DS1: 89, DS2: 91.5 |  |
| Jijja and Rai | 2019 | DL | Train/test split (75/25%) | No | ACC,SEN,SPEC |  | 50 | 98.54 |
| Natarajan and Kumarasamy | 2019 | TML | NR | No | ACC,SEN,SPEC,DSC,PPV | 0.91 | 92.07 | 94.87 |
| Chaudhari and Kulkarni | 2019 | TML | NR | No | DSC,SEN,SPEC,PPV,ACC | 0.7 (TC) | 65.1 (TC) | 99 (TC) |
| Hachemi et al. | 2019 | TML | NR | No | SEN,SPEC,DSC,HD | 0.8 | 72 |  |
| Sheela and Suganthi | 2019 | TML | NR | No | DSC,SEN,SPEC,HD | MEN: 0.78, Glioma: 0.59 | MEN: 67, Glioma: 51 |  |
| Zhang et al. | 2019 | TML | NR | No | DSC,SEN,SPEC | 0.93 | 94.6 |  |
| Grøvik et al. | 2019 | DL | Train/test split (95/5%) | Yes | PPV,DSC,AUC,SEN,SPEC | 0.79 | 94 |  |
| Eltayeb et al. | 2019 | TML | NR | No | DSC,ACC,JSC | 0.92 |  | 91.45 |
| Nagarathinam and Ponnuchamy | 2019 | TML | Train/test split (80/20%) | No | SEN,SPEC,DSC,ACC,PPV | DS1: 0.92, DS2: 0.92 | DS1: 95.9, DS2: 96.2 | DS1: 98.4, DS2: 98.7 |
| Wang et al. | 2019 | DL | Train/test split (80/20%) | Yes | DSC,HD | DS1: 0.905, DS2: 0.874, DS3: 0.908, DS4: 0.878 |  |  |
| Li et al. | 2019 | DL | Train/test split (80/20%) + Cross-validation | Yes | DSC,SEN,PPV | DS1: 0.890, DS2: 0.876, DS3: 0.845 | DS1: 89.5, DS3: 88.5 |  |
| Amin et al. | 2019 | TML | Cross-validation | No | DSC | DS1 (SVM): 0.96, DS1 (NB): 0.98, DS1 (Ensemble): 0.96, DS1 (DT): 0.97, DS1 (KNN): 1.00, DS1 (RF): 0.97, DS2 (SVM): 0.98, DS2 (NB): 0.99, DS2 (Ensemble): 0.91, DS2 (DT): 0.98, DS2 (KNN): 0.98, DS2 (RF): 0.98, DS3 (SVM): 0.98, DS3 (NB): 0.93, DS3 (Ensemble): 0.95, DS3 (DT): 0.96, DS3 (KNN): 0.95, DS3 (RF): 0.97 |  |  |
| Sun et al. | 2019 | DL | Train/test split (90/10%) | Yes | DSC,PPV,SEN | 0.84 | 89 |  |
| Chang et al. | 2019 | DL | NR | Yes | DSC,PPV,SEN | 0.8 | 95 |  |
| Tong et al. | 2019 | TML | Train/test split (78/22%) | No | JSC,DSC,SEN,SPEC | 0.89 | 93.07 |  |
| Shapey et al. | 2019 | DL | Train/test split (81/19%) | No | DSC,ASSD,RVE | 0.94 |  |  |
| Zhao et al. | 2019 | TML | Cross-validation | No | DSC,PPV,SEN,SPEC | 0.91 | 89 |  |
| Dogra et al. | 2019 | TML | NR | No | DSC,JSC,PPV,SEN,SPEC | 0.9 | 99 |  |
| Hu et al. | 2019 | DL | NR | Yes | DSC,SEN,PPV,SPEC,HD | DS1: 0.89, DS2: 0.87, DS3: 0.88 | DS1:87, DS2: 88, DS3: 90.7 |  |
| Na et al. | 2019 | TML | Train/test split (77/23%) | No | DSC | 0.79 |  |  |
| Wang et al. | 2019 | DL | NR | No | DSC | 0.72 |  |  |
| Thaha et al. | 2019 | DL | NR | No | ACC,SEN |  | 90 | 92 |
| Razzak et al. | 2019 | DL | NR | No | DSC,PPV,SEN | DS1: 0.89, DS2: 0.89 | DS1: 87.71, DS2: 88.32 |  |
| Sriramakrishnan et al. | 2019 | TML | NR | No | DSC,PPV,SEN | DS1: 0.76, DS2: 0.81 | DS1: 73, DS2: 79 |  |
| Kumar and VijayKumar | 2019 | TML | Train/test split | No | ACC,SEN,PPV,DSC | 0.95 | 95.47 | 91.17 |
| Mlynarski et al. | 2019 | DL | Train/test split (82.5/17.5%) | Yes | DSC | 0.9 |  |  |
| Mallick et al. | 2019 | DL | Cross-validation | No | SEN,SPEC,DSC,ACC | 0.93 | 94 | 93 |
| Sun et al. | 2019 | TML | NR | No | DSC,SEN,SPEC | 0.88 | 92 |  |
| Janardhanaprabhu and Malathi | 2019 | TML | NR | No | ACC,SEN,PPV,DSC,EF | 0.97 | 99.1 | 99.8 |
| Kebir et al. | 2019 | TML | NR | No | DSC,JSC | 0.69 |  |  |
| Meghana S et al. | 2019 | DL | NR | No | ACC |  |  | 95.6 |
| Iqbal et al. | 2019 | DL | Train/test split (80/20%) | Yes | DSC | 0.82 |  |  |
| Alagarsamy et al. | 2019 | TML | NR | No | DSC,JSC,SEN,SPEC,EF,MSE,PSNR | 0.96 | 98.56 |  |
| Shivhare et al. | 2019 | TML | Cross-validation | No | DSC,SEN,PPV | 0.92 | 91 |  |
| Nema et al. | 2019 | DL | Train/test split (10/90%) | Yes | DSC,SEN | DS1: 0.94, DS2: 0.94 | DS1: 88, DS2: 91 |  |
| Peng et al. | 2019 | DL | NR | Yes | SEN,PPV,DSC | 0.85 | 86 |  |
| Kalaiselvi et al. | 2019 | TML | Cross-validation | No | DSC,SEN,PPV,ACC | 0.8 | 98.45 | 83.9 |
| Yang et al. | 2019 | DL | Cross-validation | No | DSC,PPV,SEN | 0.89 | 96 |  |
| Virupakshappa and Amarapur | 2019 | TML | NR | Yes | SEN,PPV,ACC |  | 95.4 | 96 |
| Wu et al. | 2019 | TML | Train/test split (80/20%) + Cross-validation | No | DSC,SEN,SPEC,HD | 0.85 | 81.47 |  |
| Wang et al. | 2019 | DL | Train/test split (83/17%) + Cross-validation | No | DSC,SEN,PPV | DS1: 0.94, DS2: 0.91 | DS1: 92, DS2: 94 |  |
| Rehman et al. | 2019 | TML | Cross-validation | No | DSC,SEN,SPEC,PPV | 0.91 | 93 |  |
| Kharrat and Neji | 2020 | DL | Train/test split (80/20%) + Cross-validation | No | DSC | DS1: 0.92, DS2: 0.9 |  |  |
| Rehman et al. | 2020 | DL | Train/test split (60/40%) + Cross-validation | Yes | ACC |  |  | DS1: 98.32, DS2:96.97, DS3:92.67 |
| Khosravanian et al. | 2020 | TML | NR | No | DSC,HD,SEN,SPEC,MAD,JSC | 0.93 | 91.83 | 99.4 |
| Srinivas and Rao | 2020 | DL | Train/test split (70/30%) | No | DSC,JSC | 0.96 |  |  |
| Yogananda et al. | 2020 | DL | Train/test split (90/10%) + Cross-validation | Yes | DSC,HD | DS1: 0.90, DS2: 0.91, DS3: 0.9, DS4: 0.85 |  |  |
| Zhou et al. | 2020 | DL | Train/test split (92/8%) | Yes | DSC,SEN,PPV,HD | DS1: 0.91, DS2: 0.87, DS3: 0.91 | DS2: 88 |  |
| Kumar et al. | 2020 | TML | Train/test split (80/20%) | No | DSC,SEN,SPEC,ACC,PPV,NPV,MCC | 0.9 | 100 | 96.5 |
| Chen et al. | 2020 | TML | Cross-validation | No | DSC,SEN,PPV | DS1: 0.84, DS2: 0.86 | DS1: 82, DS2: 81 |  |
| Chen et al. | 2020 | DL | NR | Yes | DSC | 0.85 |  |  |
| Tjahyaningtijas et al. | 2020 | DL | Train/test split (83/17%) | No | DSC,SEN,SPEC,JSC,ACC | 0.91 | 96.9 | 99.5 |
| Khan et al. | 2020 | DL | Cross-validation | No | DSC | 0.81 |  |  |
| Zhang et al. | 2020 | DL | Train/test split (80/20%) + Cross-validation | Yes | DSC,HD | DS1: 0.88, DS2: 0.872, DS3: 0.87 |  |  |
| Zhang et al. | 2020 | DL | Train/test split (80/20%) | Yes | DSC | DS1: 0.88, DS2: 0.88 |  |  |
| Xue et al. | 2020 | DL | Cross-validation | Yes | DSC,SEN,SPEC | DS1: 0.85, DS2: 0.84, DS3: 0.83 | DS1: 96, DS2: 95, DS3: 96 |  |
| Sun et al. | 2020 | DL | NR | Yes | DSC,SEN,SPEC | DS1: 0.89, DS2: 0.90 | DS1: 0.88, DS2: 0.90 |  |
| Liu et al. | 2020 | DL | Train/test split (80/20%) | No | DSC,PPV,SEN,HD | 0.89 | 91 |  |
| Chaudhary et al. | 2020 | DL | Train/test split (80/20%) | No | DSC,PPV,SEN | 0.9 | 91 |  |
| Laukamp et al. | 2020 | DL | Train/test split (55/45%) | No | DSC | 0.82 |  |  |
| Thiruvenkadam and Perumal | 2016 | TML | NR | No | WI,DSC,SEN,SPEC,ACC | 0.79 | 86.9 | 97 |
| Gupta et al. | 2020 | TML | NR | No | ACC |  |  | 98 |
| Yepuganti et al. | 2020 | TML | NR | No | ACC,DSC,JSC | 0.99 |  | 99.56 |
| Bousabarah et al. | 2020 | DL | Train/test split (92/8%) + Cross-validation | No | DSC,SEN,PPV | 0.7 | 82 |  |
| Ejaz et al. | 2020 | TML | Cross-validation | No | DSC,JSC,MSE,PSNR | 0.98 |  |  |
| Pennig et al. | 2020 | DL | NR | Yes | DSC | 0.76 |  |  |
| Mohamed et al. | 2020 | TML | NR | No | DSC,SPEC,SEN | 0.84 |  |  |
| Ali et al. | 2020 | DL | NR | Yes | DSC | 0.91 |  |  |
| Katouli and Rahmani | 2020 | TML | NR | No | ACC,SEN,SPEC,DSC,JSC,SI,MSE,PSNR | 0.74 | MEN: 99, Glioma: 93 | MEN: 100, Glioma: 98 |
| Naceur et al. | 2020 | DL | Train/test split (70/30%) | Yes | DSC,HD,SEN,SPEC | 0.86 | 85.3 |  |
| Sharif et al. | 2020 | TML | NR | Yes | DSC,SEN,SPEC,ACC,PPV,JSC | DS1: 0.99, DS2: 0.96, DS3: 0.89, DS4: 0.95 | DS1: 98, DS2: 96, DS3: 88, DS4: 95 | DS1: 99, DS2: 97.2, DS3: 87.6, DS4: 96.5 |
| Aboelenein et al. | 2020 | DL | Train/test split (80/20%) | Yes | DSC,SEN,SPEC,HD | 0.87 | 88.3 |  |
| Zaihani et al. | 2020 | TML | NR | No | ACC |  |  | 80.2 |
| Hassen et al. | 2020 | TML | NR | No | SEN,SPEC,DSC,ACC,PPV,HD | DS2: 0.94, DS3: 0.9 | DS2: 92.4 | DS1: 98.9 |
| Chithra and Dheepa | 2020 | DL | NR | No | DSC,SEN,ACC | DS1: 0.86, DS2: 0.89, DS3: 0.9 | DS1: 86, DS2: 87, DS3: 92 | DS1: 93.3, DS2: 93.6, DS3: 94.2 |
| Kao et al. | 2020 | DL | Cross-validation | Yes | DSC,HD | DS1: 0.9, DS2: 0.91 |  |  |
| Pitchai et al. | 2020 | DL | Train/test split (55/45%) | No | DSC,SEN,SPEC,ACC,JSC | 0.89 | 87 | 91 |
| Zeineldin et al. | 2020 | DL | Train/test split (80/20%) + Cross-validation | Yes | DSC,SEN,SPEC,HD | 0.84 | 86.5 |  |
| Al-qazzaz et al. | 2020 | DL | Train/test split (75/25%) | No | DSC | 0.84 |  |  |
| Al-qazzaz et al. | 2020 | DL | Train/test split (75/25%) + Cross-validation | No | DSC | 0.85 |  |  |
| Sathish and Elango | 2020 | TML | Cross-validation | No | DSC,PPV,SEN | DS1: 0.94, DS2: 0.96 | DS1: 95.9, DS2: 95.2 |  |
| Banerjee and Mitra | 2020 | DL | Train/test split (20/80%) + Cross-validation | Yes | DSC,SEN,SPEC,HD | 0.9 | 91.3 |  |
| Tripathi et al. | 2020 | DL | NR | Yes | JSC,DSC,ACC |  |  | 98.6 |
| Kanniappan et al. | 2020 | TML | NR | No | DSC,JSC | DS1: 0.86, DS2: 0.88 |  |  |
| Debnath et al. | 2020 | TML | NR | No | DSC,SEN,SPEC | 0.95 | 92.14 |  |
| Baid et al. | 2020 | DL | NR | Yes | DSC,SEN,SPEC,HD | DS1: 0.878, DS2: 0.8475, DS3: 0.9235 |  |  |
| Hu et al. | 2020 | DL | NR | Yes | DSC,SPEC,SEN | 0.86 | 87 |  |
| Zhou et al. | 2020 | DL | NR | Yes | DSC | DS1: 0.86, DS2: 0.82, DS3: 0.87 |  |  |
| Zhou et al. | 2020 | DL | NR | Yes | DSC | DS1: 0.87, DS2: 0.84, DS3: 0.83, DS4: 0.86 |  |  |
| Barzegar and jamzad | 2020 | TML | Cross-validation | No | SEN,SPEC,DSC,JSC,HD | DS1: 0.907, DS2: 0.883, DS3: 0.878 | DS1: 88.9, DS2: 88.1, DS3: 89.6 | DS1: 99.7, DS2: 98.3, DS3: 99.7 |
| Gyorfi et al. | 2021 | TML | Leave-one-out cross-validation | No | ACC,SEN,SPEC,DSC | DS1: 0.85, DS2: 0.85 | DS1: 82, DS2: 83.76 | DS1: 98.07, DS2: 98.22 |
| Leva et al. | 2021 | DL | Train/test split (80/20%) | Yes | DSC,SEN,SPEC | DS1: 0.87, DS2: 0.88 | DS2: 90.78 |  |
| Biratu et al. | 2021 | TML | NR | No | EF,SEN,DSC,SPEC,JSC,ACC | 0.86 | 82.6 | 98.6 |
| Rai et al. | 2021 | DL | Train/test split (90/10%) | No | SEN,PPV,ACC,DSC,JSC | 0.96 | 89.7 | 99.7 |
| Mitchell et al. | 2021 | DL | Train/test split (88/12%) | No | DSC,SEN,PPV | 0.87 | 87 |  |
| Abirami et al. | 2021 | DL | Train/test split (80/20%) | No | DSC | 0.84 |  |  |
| Al-Dabagh | 2021 | TML | NR | Yes | DSC,JSC | 0.90 |  |  |
| Sohail et al. | 2021 | DL | Train/test split (88/12%) | No | DSC | DS1:0.78, DS2:0.72 |  |  |
| Sran et al. | 2021 | TML | NR | No | DSC,JSC | 0.91 |  |  |
| Saxena et al. | 2021 | TML | Cross-validation | No | SEN,SPEC,ACC,DSC | 0.92 | 97.87 | 97.89 |
| Takahashi et al. | 2021 | DL | Train/test split (80/20% and 75/25% respectively) | Yes | DSC | DS1: 0.87, DS2: 0.78 |  |  |
| Latif et al. | 2021 | DL | Train/test split (60/40%) | Yes | DSC,PPV,SEN,JSC | DS1: 0.896, DS2: 0.840, DS3: 0.839, DS4: 0.87446 | DS1: 85.3, DS2: 77.1, DS3: 78.1 |  |
| Zhang et al. | 2021 | DL | NR | Yes | DSC,SEN,SPEC,HD | 0.88 | 91 |  |
| Lei et al. | 2021 | TML | NR | Yes | DSC,SEN,PPV | 0.96 | 96 |  |
| Wang et al. | 2021 | DL | NR | Yes | DSC,HD,SEN,SPEC | 0.89 |  |  |
| Zhao et al. | 2021 | DL | Cross-validation | No | DSC,HD | 89.62 |  |  |
| Cao et al. | 2021 | DL | Train/test split (92.5/7.5%) | No | DSC,SEN,PPV | 0.84 |  |  |
| Barzegar and jamzad | 2021 | TML | Train/test split (66.6/33.3%) + Cross-validation | No | SEN,SPEC,DSC,JSC,HD | DS1: 0.9156, DS2: 0.9003, DS3: 0.9014 | DS1: 89.3, DS2: 88.7, DS3: 90.1 | DS1: 99.6, DS2: 99.1, DS3: 99.7 |
| DSC=Dice Score Coefficient; SEN=Sensitivity; ACC=Accuracy; HD=Hausdorff distance; SPEC=Specificity; PPV=Positive Predictive value; NPV=Negative Predictive value; JSC=Jaccard Score Coefficient; WI=William’s Index; SI=Similarity Index; MSE=Mean Square Error; PSNR=Peak signal-to-noise ratio; AD=Average diatance; AUC=Area Under the Curve; KNN=k-nearest neighbour; SVM=Support Vector Machine; DT=Decision Trees; NB=Naïve Bayes | | | | | | | | |

# Supplementary table 5 – Performance evaluation of detection studies

| **Author** | **Year** | **Group** | **Internal validation** | **Out-of-sample external validation** | **Performance metrics** | **SEN** | **SPEC** | **ACC** |
| --- | --- | --- | --- | --- | --- | --- | --- | --- |
| Jayachandran and Dhanasekaran | 2012 | TML | Train/test split (50/50%) | No | SEN,SPEC,ACC | 80 | 100 | 90 |
| Farjam et al. | 2012 | TML | Train/test split (32/68%) | No | SEN,FPR | 93.5 |  |  |
| Jayachandran and Dhanasekaran | 2013 | TML | Cross-validation | No | SEN,SPEC,ACC | 100 | 80 | 93 |
| Dvorák et al. | 2013 | TML | Cross-validation | No | SEN,SPEC | 87.52 | 93.14 |  |
| Dahshan et al. | 2014 | TML | Train/test split (65/35%) | No | SEN,SPEC,ACC | 100 | 92.8 | 99 |
| Bahadure et al. | 2015 | TML | NR | No | ACC,SEN,SPEC | 97.72 | 94.2 | 96.51 |
| Helen and Kamaraj | 2015 | TML | Cross-validation | No | SEN,SPEC | 97.3 | 83.3 |  |
| Thirumurugan et al. | 2016 | TML | NR | No | SEN,SPEC,ACC | DS1: 99.8, DS2: 99.6 | DS1: 99.7, DS2: 98.1 | DS1: 99.8, DS2: 99.1 |
| Banerjee et al. | 2016 | TML | NR | No | AUC |  |  |  |
| Amin et al. | 2017 | TML | Cross-validation | No | ACC,AUC,SEN,SPEC,FNR,FPR | DS1 (linear): 91.9, DS1 (cubic): 93.1, DS1 (Gaussian): 91.1, DS2 (linear): 92.4, DS2 (cubic): 89.2, DS2 (Gaussian): 89.1 | DS1 (linear): 100, DS1 (cubic): 85.6, DS1 (Gaussian): 95.6, DS2 (linear): 100, DS2 (cubic): 78.9, DS2 (Gaussian): 98.2 | DS1 (linear): 95.9, DS1 (cubic): 88.8, DS1 (Gaussian): 92.4, DS2 (linear): 97.1, DS2 (cubic): 83.2, DS2 (Gaussian): 94.5 |
| Gupta and Khanna | 2017 | TML | Cross-validation | No | ACC,SEN,SPEC,PPV | DS1: 100, DS2: 100 | DS1: 97.5, DS2: 95.8 | DS1: 98.9, DS2: 98.1 |
| Anitha and Raja | 2017 | TML | NR | No | SEN,SPEC,FPR,FNR,LPR,LNR | DS1: 96.3, DS2: 95.6 | DS1: 98.1, DS2: 98.1 |  |
| Lahmiri | 2017 | TML | Leave-one-out cross-validation | No | SEN,SPEC,ACC | 100 | 97.95 | 99.2 |
| Deepa and Emmanuel | 2018 | TML | Train/test split (63/37%) | No | PPV,SEN,DSC,ACC,SPEC,JSC | 97.24 | 99.85 | 99.84 |
| Selvapandian and Manivannan | 2018 | TML | Train/test split (50/50%) | No | ACC,SEN,SPEC,PPV,DSC | 93.46 | 96.54 | 97.75 |
| Abd-Ellah et al. | 2018 | DL | Train/test split (35/65%) | No | SEN,SPEC,ACC,BA,PPV,NPV | 99.38 | 100 | 99.55 |
| Arunkumar et al. | 2018 | TML | Cross-validation | No | SEN,SPEC,ACC | 100 | 94 | 98.7 |
| Edalati-rad and Mosleh | 2019 | TML | Cross-validation | No | SEN,SPEC,ACC | 100 | 97.43 | 98.74 |
| Song et al. | 2019 | TML | Leave-one-out cross-validation | No | ACC,SEN,SPEC | 97.3 | 99.17 | 98.36 |
| Johnpeter and Ponnuchamy | 2019 | TML | NR | No | SEN,SPEC,ACC,PPV,NPV | 96.5 | 97.7 | 98.8 |
| Amin et al. | 2019 | TML | Cross-validation | No | ACC,AUC,SEN,SPEC | DS1 (SVM): 100, DS1 (NB): 98, DS1 (Ensemble): 91, DS1 (DT): 100, DS1 (KNN): 100, DS1 (RF): 100, DS2 (SVM): 100, DS2 (NB): 100, DS2 (Ensemble): 85, DS2 (DT): 100, DS2 (KNN): 96, DS2 (RF): 96, DS3 (SVM): 94, DS3 (NB): 100, DS3 (Ensemble): 92, DS3 (DT): 100, DS3 (KNN): 100, DS3 (RF): 95 | DS1 (SVM): 87, DS1 (NB): 70, DS1 (Ensemble): 83, DS1 (DT): 70, DS1 (KNN): 78, DS1 (RF): 71, DS2 (SVM): 91, DS2 (NB): 87, DS2 (Ensemble): 100, DS2 (DT): 80, DS2 (KNN): 100, DS2 (RF): 100, DS3 (SVM): 91, DS3 (NB): 95, DS3 (Ensemble): 90, DS3 (DT): 90, DS3 (KNN): 100, DS3 (RF): 99 | DS1 (SVM): 97, DS1 (NB): 90, DS1 (Ensemble): 90, DS1 (DT): 94, DS1 (KNN): 93, DS1 (RF): 95, DS2 (SVM): 94, DS2 (NB): 97, DS2 (Ensemble): 93, DS2 (DT): 96, DS2 (KNN): 100, DS2 (RF): 96, DS3 (SVM): 97, DS3 (NB): 98, DS3 (Ensemble): 86, DS3 (DT): 97, DS3 (KNN): 97, DS3 (RF): 97 |
| Alam et al. | 2019 | TML | NR | No | ACC,SEN,SPEC | 97.4 | 100 | 97.5 |
| Atici et al. | 2019 | DL | Cross-validation | No | ACC,PPV,SEN | 84.6 |  | 97.1 |
| Sriramakrishnan et al. | 2019 | TML | NR | No | SEN,SPEC,ACC | 95.5 | 97.9 | 97.2 |
| Kebir et al. | 2019 | TML | NR | No | SEN,SPEC,ACC | 73 | 66 | 69 |
| Kalaiselvi et al. | 2019 | TML | Cross-validation | No | ACC,SEN,SPEC | DS1: 91, DS2: 93.8 | DS1: 97, DS2: 100 | DS1: 95, DS2: 97.6 |
| Çinar and Yildirim | 2020 | DL | NR | No | SEN,SPEC,ACC,FPR,FNR,FDR,DSC | 94.7 | 100 | 97.01 |
| Devanathan and Venkatachalapathy | 2020 | DL | NR | No | SEN,SPEC,ACC | 97.94 | 98.08 | 98.02 |
| Dikici et al. | 2020 | DL | Cross-validation | No | SEN | 90 |  |  |
| Dheepa and Chithra | 2020 | DL | NR | No | ACC,SEN,SPEC,PPV | 97 | 97 | 95 |
| Gurunathan and Krishnan | 2020 | DL | Train/test split (60/40%) | No | SEN,SPEC,ACC | 97.2 | 98.9 | 98.5 |
| Wang et al. | 2020 | TML | Leave-one-out cross-validation | No | ACC,SEN,SPEC | 100 | 99.81 | 99.82 |
| Kesav and Rajini | 2020 | TML | Cross-validation | No | SEN,SPEC,PPV,ACC | 98.6 | 97.72 | 98.26 |
| Pennig et al. | 2020 | DL | NR | Yes | SEN | 95.7 |  |  |
| Murali and Meena | 2020 | TML | NR | No | SEN,SPEC,ACC | 97.8 | 100 | 97.3 |
| Sathish and Elango | 2020 | TML | Cross-validation | No | MSE,ACC |  |  | DS1: 89.5, DS2: 87.2 |
| Kaur and Ghandi | 2020 | DL | Cross-validation | No | ACC,AUC,SEN,SPEC,FNR,FPR | DS1: 100, DS2: 100, DS3: 100, DS4: 95 | DS1: 100, DS2: 100, DS3: 100, DS4: 93 | DS1: 100, DS2: 100, DS3: 100, DS4: 94 |
| Thangarajan and Chokkalingam | 2020 | DL | Train/test split (70/30%) | No | ACC,SEN,SPEC,PPV,FPR,FNR,NPV,FDR | 93.9 | 93.3 | 93.7 |
| Kalaiselvi et al. | 2020 | DL | NR | Yes | FA,MA,ACC |  |  | 88.91 |
| Rajinikanth et al. | 2020 | DL | Cross-validation | No | ACC,PPV,SEN,SPEC,NPV | DS1 (DT): 97, DS1 (KNN): 96.8, DS1 (SVM-Linear): 98, DS1 (SVM-RBF): 98.8, DS2 (SVM-RBF): 98.3, DS3 (SVM-RBF): 98.8 | DS1 (DT): 98.2, DS1 (KNN): 98.5, DS1 (SVM-Linear): 98.7, DS1 (SVM-RBF): 99.3, DS2 (SVM-RBF): 97.8, DS3 (SVM-RBF): 97 | DS1 (DT): 97.7, DS1 (KNN): 97.8, DS1 (SVM-Linear): 98.4, DS1 (SVM-RBF): 99.1, DS2 (SVM-RBF): 98, DS3 (SVM-RBF): 98.2 |
| Huang et al. | 2020 | DL | Train/test split (90/10%) | No | ACC,SEN,SPEC,PPV | DS1: 98.8, DS2: 98.6 |  | DS1: 99.2, DS2: 98 |
| Chen et al. | 2021 | TML | Cross-validation | No | SEN,SPEC,ACC | 97 | 95.4 | 98 |
| Rai et al. | 2021 | DL | Train/test split (90/10%) | No | ACC,PPV,SEN | 89.7 |  | 99.7 |
| Patil and hamde | 2021 | TML | Train/test split (70/30%) | No | SEN,SPEC,ACC,PPV | 100 | 100 | 100 |
| Simaiya et al. | 2021 | TML | Train/test split (60/40%) | No | ACC,SEN,SPEC,PPV | 88.21 | 88.22 | 88.9 |
| Tejas P and Padma | 2021 | TML | NR | No | SEN,SPEC,PPV,ACC | 92.5 | 100 | 94 |
| SEN=Sensitivity; ACC=Accuracy; SPEC=Specificity; PPV=Positive Predictive value; NPV=Negative Predictive value; AUC=Area Under the Curve; KNN=k-nearest neighbour; SVM=Support Vector Machine; DT=Decision Trees; NB=Naïve Bayes | | | | | | | | |

# Supplementary table 6: Detailed CLAIM quality reporting assessment for segmentation studies

| **Author** | **Year** | **1** | **2** | **3** | **4** | **5** | **6** | **7** | **8** | **9** | **10** | **11** | **12** | **13** | **14** | **15** | **16** | **17** | **18** | **19** | **20** | **21** | **22** | **23** | **24** | **25** | **26** | **27** | **28** | **29** | **30** | **31** | **32** | **33** | **34** | **35** | **36** | **37** | **38** | **39** | **40** | **41** | **42** |
| --- | --- | --- | --- | --- | --- | --- | --- | --- | --- | --- | --- | --- | --- | --- | --- | --- | --- | --- | --- | --- | --- | --- | --- | --- | --- | --- | --- | --- | --- | --- | --- | --- | --- | --- | --- | --- | --- | --- | --- | --- | --- | --- | --- |
| Fletcher-Heath et al. | 2001 | Y | Y | Y | Y | Y | Y | Y | Y | Y | / | Y | N | N | P | P | P | P | P | N | Y | / | Y | Y | Y | Y | Y | / | Y | N | N | N | Y | N | N | Y | N | N | N | Y | N | N | N |
| Kaus et al. | 2001 | N | N | P | Y | Y | Y | Y | Y | N | / | Y | N | N | Y | Y | Y | Y | Y | N | N | / | N | Y | Y | N | Y | / | Y | Y | N | N | N | N | N | Y | N | Y | N | Y | N | N | N |
| Prastawa et al. | 2004 | Y | P | Y | Y | Y | Y | Y | Y | Y | / | Y | N | N | Y | Y | Y | Y | Y | N | N | / | Y | Y | Y | N | Y | / | Y | N | N | N | N | N | N | Y | N | N | Y | Y | N | N | N |
| Xie et al. | 2005 | Y | Y | Y | Y | Y | Y | P | N | Y | / | Y | N | N | Y | Y | Y | Y | Y | N | N | / | Y | Y | Y | Y | Y | / | Y | Y | N | N | N | N | N | Y | Y | N | N | Y | Y | N | Y |
| Corso et al. | 2008 | Y | P | Y | Y | Y | Y | Y | Y | Y | / | Y | N | N | Y | Y | Y | Y | Y | N | Y | / | Y | Y | Y | Y | Y | / | Y | N | N | N | Y | N | N | Y | N | Y | Y | Y | N | N | N |
| Nie et al. | 2009 | Y | P | Y | Y | Y | Y | Y | Y | Y | / | Y | N | N | Y | Y | Y | Y | Y | N | N | / | Y | Y | Y | Y | Y | / | Y | N | N | N | N | N | N | Y | N | N | N | Y | Y | N | Y |
| Taheri et al. | 2010 | Y | P | Y | Y | Y | Y | Y | N | Y | / | Y | N | N | N | N | N | N | N | N | N | / | Y | Y | Y | Y | Y | / | Y | N | N | N | N | N | N | Y | N | N | N | Y | N | N | N |
| Hsieh et al. | 2011 | Y | Y | Y | Y | Y | Y | Y | Y | Y | / | Y | N | N | P | P | P | P | P | N | N | / | Y | Y | Y | Y | Y | / | Y | Y | N | N | N | N | N | Y | Y | N | N | Y | N | N | N |
| Hamamci et al. | 2012 | Y | Y | Y | Y | Y | Y | Y | Y | Y | / | Y | N | N | Y | Y | Y | Y | Y | N | N | / | Y | Y | Y | Y | Y | / | Y | Y | Y | N | N | N | N | Y | N | N | P | Y | N | N | N |
| Sanjuàn et al. | 2013 | Y | Y | Y | Y | Y | Y | Y | Y | Y | / | Y | N | N | Y | Y | Y | Y | Y | N | N | / | Y | Y | Y | N | Y | / | Y | Y | N | N | N | N | Y | Y | Y | Y | Y | Y | Y | Y | Y |
| Jiang et al. | 2013 | Y | Y | Y | Y | Y | Y | Y | Y | Y | / | Y | N | N | Y | Y | Y | Y | Y | N | N | / | Y | Y | Y | Y | Y | / | Y | Y | N | N | N | N | N | Y | N | N | Y | Y | Y | N | Y |
| Wu et al. | 2013 | Y | Y | Y | Y | Y | Y | Y | Y | Y | / | Y | N | N | P | P | P | P | P | N | Y | / | Y | Y | Y | N | Y | / | Y | Y | Y | N | Y | N | N | Y | Y | N | Y | Y | Y | N | Y |
| Kharrat et al. | 2014 | Y | Y | Y | Y | Y | Y | Y | N | Y | / | Y | N | N | N | N | N | N | N | N | Y | / | Y | Y | Y | Y | Y | / | Y | N | Y | N | Y | N | Y | Y | N | N | N | Y | N | N | N |
| Tustison et al. | 2014 | Y | Y | Y | Y | Y | Y | Y | Y | Y | / | Y | N | N | Y | Y | Y | Y | Y | N | Y | / | Y | Y | Y | Y | Y | Y | Y | N | Y | N | Y | N | N | Y | N | N | N | Y | N | Y | N |
| Wu et al. | 2014 | Y | Y | Y | Y | Y | Y | Y | Y | Y | / | Y | N | N | Y | Y | Y | Y | Y | N | Y | / | Y | Y | Y | Y | Y | / | Y | Y | Y | N | Y | N | N | Y | Y | Y | Y | Y | Y | N | Y |
| Demirhan et al | 2015 | Y | Y | Y | Y | Y | Y | Y | Y | Y | / | Y | N | N | N | N | N | N | N | N | Y | / | Y | Y | Y | Y | Y | / | Y | N | N | N | N | N | N | Y | N | N | N | Y | N | N | N |
| Abdel-Maksoud et al. | 2015 | Y | P | Y | Y | Y | Y | Y | N | Y | / | Y | N | N | Y | Y | Y | Y | Y | N | N | / | Y | Y | Y | N | Y | / | Y | N | Y | N | N | N | N | Y | N | N | N | Y | N | N | N |
| Ali et al. | 2015 | Y | P | Y | Y | Y | Y | Y | N | Y | / | Y | N | N | Y | Y | Y | Y | Y | N | N | / | Y | Y | Y | N | Y | / | Y | N | N | N | N | N | N | Y | N | N | N | Y | N | N | N |
| Njeh et al. | 2015 | Y | Y | Y | Y | Y | Y | Y | Y | Y | / | Y | N | N | Y | Y | Y | Y | Y | N | Y | / | Y | Y | Y | Y | Y | / | Y | N | Y | N | Y | N | N | Y | N | N | N | Y | N | N | N |
| Albarracín et al. | 2015 | Y | Y | Y | Y | Y | Y | Y | Y | Y | / | Y | N | N | Y | Y | Y | Y | Y | N | Y | / | Y | Y | Y | N | Y | / | Y | N | Y | N | Y | N | N | Y | Y | N | N | Y | N | N | N |
| Bahadure et al. | 2015 | Y | Y | Y | Y | Y | Y | Y | N | Y | / | Y | N | N | Y | Y | Y | Y | Y | N | N | / | Y | Y | Y | N | Y | / | Y | N | N | N | N | N | N | Y | N | N | N | Y | N | N | N |
| Nabizadeh and Kubat | 2015 | Y | P | Y | Y | Y | Y | Y | Y | Y | / | Y | N | N | Y | Y | Y | Y | Y | N | Y | / | Y | Y | Y | Y | Y | / | Y | Y | Y | N | Y | N | N | Y | Y | N | Y | Y | N | N | N |
| Dvorák et al. | 2015 | Y | Y | Y | Y | Y | Y | Y | N | Y | / | Y | N | N | Y | Y | Y | Y | Y | N | Y | / | Y | Y | Y | Y | Y | / | Y | Y | Y | N | Y | N | N | Y | Y | N | N | Y | Y | N | Y |
| Szwarc et al. | 2015 | Y | Y | Y | Y | Y | Y | Y | Y | Y | / | Y | N | N | Y | Y | Y | Y | Y | N | Y | / | Y | Y | Y | N | Y | / | Y | Y | N | N | N | N | Y | Y | Y | N | Y | Y | N | N | N |
| Steed et al. | 2015 | Y | Y | Y | Y | Y | Y | Y | Y | Y | / | Y | N | N | Y | Y | Y | Y | Y | N | N | / | Y | Y | Y | Y | Y | / | Y | Y | Y | N | Y | N | N | Y | Y | Y | N | Y | N | N | N |
| Zhan et al. | 2015 | Y | P | Y | Y | Y | Y | Y | N | Y | / | Y | N | N | Y | Y | Y | Y | Y | N | N | / | Y | Y | Y | Y | Y | / | Y | N | N | N | N | N | N | Y | N | N | N | Y | Y | N | Y |
| Akkus et al. | 2015 | Y | Y | Y | Y | Y | Y | Y | Y | Y | / | Y | N | N | Y | Y | Y | Y | Y | N | Y | / | Y | Y | Y | Y | Y | / | Y | Y | Y | N | Y | N | N | Y | Y | N | Y | Y | Y | N | Y |
| Hasan et al. | 2016 | Y | Y | Y | Y | Y | Y | Y | Y | Y | / | Y | N | N | P | P | P | P | P | N | Y | / | Y | Y | Y | Y | Y | / | Y | Y | Y | N | Y | N | N | Y | Y | N | N | Y | Y | N | Y |
| Mbuyamba et al. | 2016 | Y | P | Y | Y | Y | Y | Y | N | Y | / | Y | N | N | Y | Y | Y | Y | Y | N | N | / | Y | Y | Y | N | Y | / | Y | Y | N | N | N | N | N | Y | Y | N | Y | Y | Y | N | Y |
| Vishnuvarthanan et al. | 2016 | Y | P | Y | Y | Y | Y | Y | N | Y | / | Y | N | N | P | P | P | P | N | N | N | / | Y | Y | Y | N | Y | / | Y | N | N | N | N | N | N | Y | N | N | N | Y | N | N | N |
| Thiruvenkadam and Nagarajan | 2020 | Y | Y | Y | Y | Y | Y | Y | Y | Y | / | Y | N | N | Y | Y | Y | Y | Y | N | N | / | Y | Y | Y | N | Y | / | Y | N | N | N | N | N | N | Y | N | N | N | Y | N | N | N |
| Zhao and Jia | 2016 | Y | Y | Y | Y | Y | Y | Y | Y | Y | / | Y | N | N | Y | Y | Y | Y | Y | N | N | / | Y | Y | Y | Y | Y | / | Y | P | N | N | N | N | N | Y | P | N | N | Y | Y | N | Y |
| Cordier et al. | 2016 | Y | P | Y | Y | Y | Y | Y | Y | Y | / | Y | N | N | Y | Y | Y | Y | Y | N | Y | / | Y | Y | Y | Y | Y | / | Y | Y | Y | N | Y | N | N | Y | Y | Y | Y | Y | N | N | N |
| Pereira et al. | 2016 | Y | Y | Y | Y | Y | Y | Y | Y | Y | / | Y | N | N | Y | Y | Y | Y | Y | N | Y | / | Y | Y | Y | Y | Y | / | Y | P | Y | N | Y | N | N | Y | P | N | N | Y | N | N | N |
| Koley et al. | 2016 | Y | Y | Y | Y | Y | Y | Y | Y | Y | / | Y | N | N | N | N | N | N | N | N | Y | / | Y | Y | Y | Y | Y | Y | Y | N | Y | N | Y | N | P | Y | N | N | N | Y | N | N | N |
| Liu et al. | 2016 | Y | Y | Y | Y | Y | Y | Y | Y | Y | / | Y | N | N | Y | Y | Y | Y | Y | N | N | / | Y | Y | Y | Y | Y | / | Y | Y | Y | N | N | N | N | Y | Y | N | N | Y | Y | N | Y |
| Li et al. | 2016 | Y | Y | Y | Y | Y | Y | Y | Y | Y | / | Y | N | N | Y | Y | Y | Y | Y | N | Y | / | Y | Y | Y | Y | Y | / | Y | Y | Y | N | Y | N | N | Y | N | N | N | Y | Y | N | Y |
| Vishnuvarthanan et al. | 2017 | Y | Y | Y | Y | Y | Y | Y | N | Y | / | Y | N | N | Y | Y | Y | Y | Y | N | Y | / | Y | Y | Y | N | Y | / | Y | N | N | N | N | N | P | Y | N | N | N | Y | N | N | N |
| Kamnitsas et al. | 2017 | Y | Y | Y | Y | Y | Y | Y | Y | Y | / | Y | N | N | Y | Y | Y | Y | Y | N | Y | / | Y | Y | Y | Y | Y | Y | Y | P | Y | N | Y | N | N | Y | P | N | P | Y | Y | Y | Y |
| Havaei et al. | 2017 | Y | Y | Y | Y | Y | Y | Y | Y | Y | / | Y | N | N | Y | Y | Y | Y | Y | N | Y | / | Y | Y | Y | Y | Y | / | Y | N | Y | N | Y | N | N | Y | N | N | N | Y | N | Y | N |
| Soltaninejad et al. | 2017 | Y | Y | Y | Y | Y | Y | Y | Y | Y | / | Y | N | N | Y | Y | Y | Y | Y | N | Y | / | Y | Y | Y | Y | Y | Y | Y | Y | Y | N | Y | N | Y | Y | Y | Y | Y | Y | Y | N | Y |
| Gupta and Khanna | 2017 | Y | Y | Y | Y | Y | Y | Y | Y | Y | / | Y | N | N | Y | Y | Y | Y | Y | N | Y | / | Y | Y | Y | Y | Y | / | Y | Y | Y | N | Y | N | N | Y | Y | N | N | Y | N | N | N |
| Anitha and Raja | 2017 | Y | P | Y | Y | Y | Y | Y | Y | Y | / | Y | N | N | Y | Y | Y | Y | Y | N | N | / | Y | Y | Y | P | Y | / | Y | N | N | N | N | N | N | Y | N | N | N | Y | N | N | N |
| Cui et al. | 2017 | Y | Y | Y | Y | Y | Y | Y | Y | Y | / | Y | N | N | Y | Y | Y | Y | Y | N | Y | / | Y | Y | Y | Y | Y | / | Y | N | N | N | Y | N | N | Y | N | N | N | Y | N | N | N |
| Sasikanth and Kumar | 2017 | Y | P | Y | Y | Y | Y | Y | N | Y | / | Y | N | N | Y | Y | Y | Y | Y | N | Y | / | Y | Y | Y | Y | Y | / | Y | N | N | N | Y | N | N | Y | N | N | N | Y | N | N | N |
| Imtiaz et al. | 2017 | Y | P | Y | Y | Y | Y | Y | Y | Y | / | Y | N | N | Y | Y | Y | Y | Y | N | Y | / | Y | Y | Y | Y | Y | Y | Y | N | Y | N | Y | N | N | Y | N | N | N | Y | N | N | N |
| Kaur et al. | 2017 | Y | Y | Y | Y | Y | Y | Y | Y | Y | / | Y | N | N | P | P | P | P | P | N | N | / | Y | Y | Y | N | Y | / | Y | Y | Y | N | N | N | N | Y | Y | N | Y | Y | N | N | Y |
| Rajinikanth et al. | 2017 | Y | P | Y | Y | Y | Y | Y | N | Y | / | Y | N | N | Y | Y | Y | Y | Y | N | N | / | Y | Y | Y | Y | Y | / | Y | N | N | N | Y | N | N | Y | N | N | N | Y | N | N | N |
| Rajinikanth et al. | 2017 | Y | Y | Y | Y | Y | Y | Y | N | Y | / | Y | N | N | Y | Y | Y | Y | Y | N | N | / | Y | Y | Y | Y | Y | / | Y | N | N | N | Y | N | N | Y | N | N | N | Y | N | N | N |
| Liu et al. | 2017 | Y | Y | Y | Y | Y | Y | Y | Y | Y | / | Y | N | N | Y | Y | Y | Y | Y | N | Y | / | Y | Y | Y | Y | Y | / | Y | Y | Y | N | Y | N | N | Y | Y | N | N | Y | N | N | N |
| Zhuge et al. | 2017 | Y | Y | Y | Y | Y | Y | Y | Y | Y | / | Y | N | N | Y | Y | Y | Y | Y | N | Y | / | Y | Y | Y | Y | Y | / | Y | N | Y | N | Y | N | N | Y | N | N | N | Y | N | N | N |
| Li et al. | 2017 | Y | Y | Y | Y | Y | Y | Y | Y | Y | / | Y | N | N | Y | Y | Y | Y | Y | N | Y | / | Y | Y | Y | Y | Y | / | Y | N | Y | N | Y | N | P | Y | N | N | Y | Y | Y | N | Y |
| Selvapandian and Manivannan | 2018 | Y | P | Y | Y | Y | Y | Y | Y | Y | / | Y | N | N | Y | Y | Y | Y | Y | N | N | / | Y | Y | Y | N | Y | / | Y | N | Y | N | Y | N | N | Y | N | N | N | Y | N | N | N |
| Raju et al. | 2018 | Y | Y | Y | Y | Y | Y | Y | N | Y | / | Y | N | N | Y | Y | Y | Y | Y | N | Y | / | Y | Y | Y | Y | Y | / | Y | N | Y | N | Y | N | N | Y | N | N | N | Y | N | N | N |
| Essadike et al. | 2018 | Y | Y | Y | Y | Y | Y | Y | N | Y | / | Y | N | N | Y | Y | Y | Y | Y | N | N | / | Y | Y | Y | Y | Y | / | Y | Y | Y | Y | N | N | N | Y | Y | N | Y | Y | N | N | N |
| Kermi et al. | 2018 | Y | Y | Y | Y | Y | Y | Y | Y | Y | / | Y | N | N | Y | Y | Y | Y | Y | N | N | / | Y | Y | Y | Y | Y | / | Y | Y | Y | N | N | N | N | Y | Y | Y | Y | Y | Y | N | Y |
| Pinto et al. | 2018 | Y | Y | Y | Y | Y | Y | Y | Y | Y | / | Y | N | N | Y | Y | Y | Y | Y | N | Y | / | Y | Y | Y | Y | Y | Y | Y | Y | Y | Y | Y | N | N | Y | Y | Y | N | Y | Y | N | Y |
| Narayanan et al. | 2018 | Y | Y | Y | Y | Y | Y | Y | N | Y | / | Y | N | N | Y | Y | Y | Y | Y | N | N | / | Y | Y | Y | Y | Y | / | Y | N | N | N | N | N | N | Y | N | N | N | Y | N | N | N |
| Ma et al. | 2018 | Y | P | Y | Y | Y | Y | Y | Y | Y | / | Y | N | N | Y | Y | Y | Y | Y | N | Y | / | Y | Y | Y | Y | Y | Y | Y | N | Y | N | Y | N | N | Y | N | Y | Y | Y | N | N | N |
| Amin et al. | 2018 | Y | P | Y | Y | Y | Y | Y | Y | Y | / | Y | N | N | Y | Y | Y | Y | Y | N | N | / | Y | Y | Y | Y | Y | / | Y | N | Y | N | Y | N | N | Y | N | N | N | Y | N | N | N |
| Tong et al. | 2018 | Y | Y | Y | Y | Y | Y | Y | Y | Y | / | Y | N | N | Y | Y | Y | Y | Y | N | Y | / | Y | Y | Y | Y | Y | / | Y | N | Y | N | Y | N | N | Y | N | N | N | Y | N | N | N |
| Laukamp et al. | 2018 | Y | Y | Y | Y | Y | Y | Y | Y | Y | / | Y | N | N | Y | Y | Y | Y | Y | N | Y | / | Y | Y | Y | Y | Y | / | Y | Y | Y | N | Y | Y | Y | Y | Y | Y | Y | Y | N | N | N |
| Lim and Mandava | 2018 | Y | Y | Y | Y | Y | Y | Y | Y | Y | / | Y | N | N | Y | Y | Y | Y | Y | N | N | / | Y | Y | Y | N | Y | / | Y | Y | N | N | N | N | N | Y | Y | N | Y | Y | N | N | N |
| Szilágyi et al. | 2018 | Y | Y | Y | Y | Y | Y | Y | Y | Y | / | Y | N | N | Y | Y | Y | Y | Y | N | N | / | Y | Y | Y | Y | Y | Y | Y | N | Y | N | N | N | N | Y | N | N | N | Y | N | N | Y |
| Abd-Ellah et al. | 2018 | Y | Y | Y | Y | Y | Y | Y | Y | Y | / | Y | N | N | Y | Y | Y | Y | Y | N | Y | / | Y | Y | Y | Y | Y | / | Y | N | N | N | Y | N | N | Y | N | N | N | Y | N | N | N |
| Angulakshmi et al. | 2018 | Y | P | Y | Y | Y | Y | Y | Y | Y | / | Y | N | N | Y | Y | Y | Y | Y | N | N | / | Y | Y | Y | Y | Y | / | Y | N | N | N | N | N | N | Y | N | N | N | Y | N | N | N |
| Perkuhn et al. | 2018 | Y | Y | Y | Y | Y | Y | Y | Y | Y | / | Y | N | N | Y | Y | Y | Y | Y | N | Y | / | Y | Y | Y | Y | Y | / | Y | Y | Y | N | Y | Y | Y | Y | Y | N | Y | Y | N | N | N |
| Soltaninejad et al. | 2018 | Y | Y | Y | Y | Y | Y | Y | Y | Y | / | Y | N | N | Y | Y | Y | Y | Y | N | Y | / | Y | Y | Y | Y | Y | / | Y | Y | Y | N | Y | N | Y | Y | Y | Y | Y | Y | Y | N | N |
| Naceur et al. | 2018 | Y | Y | Y | Y | Y | Y | Y | Y | Y | / | Y | N | N | Y | Y | Y | Y | Y | N | N | / | Y | Y | Y | Y | Y | Y | Y | N | N | N | N | N | N | Y | N | N | N | Y | N | N | N |
| Charron et al. | 2018 | Y | P | Y | Y | Y | Y | Y | Y | Y | / | Y | N | N | Y | Y | Y | Y | Y | N | Y | / | Y | Y | Y | Y | Y | / | Y | Y | Y | N | Y | N | N | Y | Y | N | Y | Y | N | N | Y |
| Li et al. | 2018 | Y | Y | Y | Y | Y | Y | Y | Y | Y | / | Y | N | N | Y | Y | Y | Y | Y | N | Y | / | Y | Y | Y | N | Y | / | Y | Y | Y | N | Y | N | N | Y | Y | N | N | Y | N | N | N |
| Zaouche et al. | 2018 | Y | Y | Y | Y | Y | Y | Y | N | Y | / | Y | N | N | Y | Y | Y | Y | Y | N | N | / | Y | Y | Y | Y | Y | / | Y | Y | N | N | N | N | N | Y | Y | N | N | Y | N | N | N |
| Hussain et al. | 2018 | Y | Y | Y | Y | Y | Y | Y | Y | Y | / | Y | N | N | Y | Y | Y | Y | Y | N | Y | / | Y | Y | Y | Y | Y | / | Y | Y | Y | N | Y | N | N | Y | Y | N | N | Y | N | N | N |
| Iqbal et al. | 2018 | Y | P | Y | Y | Y | Y | Y | Y | Y | / | Y | N | N | Y | Y | Y | Y | Y | N | Y | / | Y | Y | Y | Y | Y | / | Y | N | Y | N | Y | N | N | Y | N | N | N | Y | N | N | Y |
| Cui et al. | 2018 | Y | Y | Y | Y | Y | Y | Y | Y | Y | / | Y | N | N | Y | Y | Y | Y | Y | N | Y | / | Y | Y | Y | Y | Y | / | Y | N | Y | N | Y | N | N | Y | N | N | N | Y | Y | N | Y |
| Bonte et al. | 2018 | Y | Y | Y | Y | Y | Y | Y | Y | Y | / | Y | N | N | Y | Y | Y | Y | Y | N | Y | / | Y | Y | Y | Y | Y | Y | Y | P | Y | N | Y | N | N | Y | P | N | Y | Y | N | N | Y |
| Zhan et al. | 2018 | Y | P | Y | Y | Y | Y | Y | N | Y | / | Y | N | N | Y | Y | Y | Y | Y | N | N | / | Y | Y | Y | Y | Y | / | Y | N | Y | N | Y | N | N | Y | N | N | N | Y | Y | N | Y |
| Virupakshappa and Basavaraj | 2018 | Y | Y | Y | Y | Y | Y | Y | Y | Y | / | Y | N | N | Y | Y | Y | Y | Y | N | N | / | Y | Y | Y | Y | Y | / | Y | N | N | N | Y | N | N | Y | N | N | N | Y | N | N | N |
| Zhao et al. | 2018 | Y | Y | Y | Y | Y | Y | Y | Y | Y | / | Y | N | N | Y | Y | Y | Y | Y | N | N | / | Y | Y | Y | Y | Y | / | Y | N | Y | N | Y | N | N | Y | N | N | N | Y | Y | N | Y |
| Zhao et al. | 2018 | Y | Y | Y | Y | Y | Y | Y | Y | Y | / | Y | N | N | Y | Y | Y | Y | Y | N | N | / | Y | Y | Y | Y | Y | / | Y | Y | Y | N | N | N | N | Y | N | N | N | Y | N | N | N |
| Jijja and Rai | 2019 | Y | P | Y | Y | Y | Y | N | N | Y | / | Y | N | N | N | N | N | N | N | N | Y | / | Y | Y | Y | N | Y | / | Y | N | N | N | N | N | N | Y | N | N | N | Y | N | N | N |
| Natarajan and Kumarasamy | 2019 | Y | Y | Y | Y | Y | Y | P | P | Y | / | Y | N | N | N | N | N | N | N | N | N | / | Y | Y | Y | Y | Y | / | Y | N | N | N | N | N | N | Y | N | N | N | Y | N | N | N |
| Chaudhari and Kulkarni | 2019 | Y | P | Y | Y | Y | Y | Y | N | Y | / | Y | N | N | Y | Y | Y | Y | Y | N | N | / | Y | Y | Y | Y | Y | / | Y | N | N | N | N | N | N | Y | N | N | N | Y | N | N | N |
| Hachemi et al. | 2019 | Y | P | Y | Y | Y | Y | Y | N | Y | / | Y | N | N | Y | Y | Y | Y | Y | N | N | / | Y | Y | Y | Y | Y | / | Y | N | N | N | N | N | N | Y | N | N | N | Y | N | N | N |
| Sheela and Suganthi | 2019 | Y | P | Y | Y | Y | Y | Y | Y | Y | / | Y | N | N | Y | Y | Y | Y | Y | N | N | / | Y | Y | Y | N | Y | / | Y | N | N | N | N | N | N | Y | N | N | N | Y | N | N | N |
| Zhang et al. | 2019 | Y | P | Y | Y | Y | Y | Y | N | Y | / | Y | N | N | Y | Y | Y | Y | Y | N | N | / | Y | Y | Y | N | Y | / | Y | N | N | N | N | N | N | Y | N | N | N | Y | Y | N | Y |
| Grøvik et al. | 2019 | Y | Y | Y | Y | Y | Y | Y | Y | Y | / | Y | N | N | Y | Y | Y | Y | Y | N | Y | / | Y | Y | Y | Y | Y | / | Y | Y | Y | Y | Y | Y | Y | Y | Y | Y | Y | Y | Y | N | Y |
| Eltayeb et al. | 2019 | Y | Y | Y | Y | Y | Y | Y | N | Y | / | Y | N | N | Y | Y | Y | Y | Y | N | N | / | Y | Y | Y | N | Y | / | Y | Y | N | N | N | N | N | Y | Y | N | N | Y | N | N | N |
| Nagarathinam and Ponnuchamy | 2019 | Y | P | Y | Y | Y | Y | Y | N | Y | / | Y | N | N | Y | Y | Y | Y | Y | N | Y | / | Y | Y | Y | Y | Y | / | Y | N | Y | N | Y | N | N | Y | N | N | N | Y | N | N | N |
| Wang et al. | 2019 | Y | Y | Y | Y | Y | Y | Y | Y | Y | / | Y | N | N | Y | Y | Y | Y | Y | N | Y | / | Y | Y | Y | Y | Y | / | Y | Y | Y | N | Y | N | N | Y | Y | N | N | Y | Y | N | Y |
| Li et al. | 2019 | Y | Y | Y | Y | Y | Y | Y | Y | Y | / | Y | N | N | Y | Y | Y | Y | Y | N | Y | / | Y | Y | Y | Y | Y | / | Y | Y | Y | N | Y | N | N | Y | Y | N | N | Y | Y | N | Y |
| Amin et al. | 2019 | Y | Y | Y | Y | Y | Y | Y | Y | Y | / | Y | N | N | Y | Y | Y | Y | Y | N | Y | / | Y | Y | Y | Y | Y | Y | Y | N | Y | N | Y | N | P | Y | N | N | N | Y | N | N | N |
| Sun et al. | 2019 | Y | Y | Y | Y | Y | Y | Y | Y | Y | / | Y | N | N | Y | Y | Y | Y | Y | N | Y | / | Y | Y | Y | Y | Y | / | Y | N | Y | N | Y | N | N | Y | N | N | N | Y | Y | N | Y |
| Chang et al. | 2019 | Y | Y | Y | Y | Y | Y | Y | Y | Y | / | Y | N | N | Y | Y | Y | Y | Y | N | Y | / | Y | Y | Y | Y | Y | / | Y | N | Y | N | Y | N | N | Y | N | N | N | Y | Y | N | Y |
| Tong et al. | 2019 | Y | Y | Y | Y | Y | Y | Y | Y | Y | / | Y | N | N | Y | Y | Y | Y | Y | N | Y | / | Y | Y | Y | Y | Y | / | Y | N | Y | N | Y | N | N | Y | Y | N | N | Y | Y | N | Y |
| Shapey et al. | 2019 | Y | Y | Y | Y | Y | Y | Y | Y | Y | / | Y | N | N | Y | Y | Y | Y | Y | N | Y | / | Y | Y | Y | Y | Y | Y | Y | Y | Y | N | Y | N | P | Y | Y | N | Y | Y | Y | N | Y |
| Zhao et al. | 2019 | Y | Y | Y | Y | Y | Y | Y | Y | Y | / | Y | N | N | Y | Y | Y | Y | Y | N | Y | / | Y | Y | Y | Y | Y | Y | Y | N | Y | N | Y | N | N | Y | N | N | N | Y | N | N | N |
| Dogra et al. | 2019 | Y | Y | Y | Y | Y | Y | Y | N | Y | / | Y | N | N | Y | Y | Y | Y | Y | N | N | / | Y | Y | Y | Y | Y | / | Y | Y | N | N | N | N | N | Y | Y | N | N | Y | N | N | N |
| Hu et al. | 2019 | Y | P | Y | Y | Y | Y | Y | Y | Y | / | Y | N | N | Y | Y | Y | Y | Y | N | Y | / | Y | Y | Y | Y | Y | / | Y | Y | Y | N | Y | N | N | Y | Y | N | N | Y | N | N | N |
| Na et al. | 2019 | Y | Y | Y | Y | Y | Y | Y | Y | Y | / | Y | N | N | Y | Y | Y | Y | Y | N | Y | / | Y | Y | Y | Y | Y | Y | Y | N | Y | N | Y | N | N | Y | N | N | N | Y | N | N | N |
| Wang et al. | 2019 | Y | Y | Y | Y | Y | Y | P | N | Y | / | Y | N | N | Y | Y | Y | Y | Y | N | N | / | Y | Y | Y | Y | Y | / | Y | P | N | N | N | N | N | Y | P | N | Y | Y | Y | N | Y |
| Thaha et al. | 2019 | Y | P | Y | Y | Y | Y | Y | Y | Y | / | Y | N | N | Y | Y | Y | Y | Y | N | N | / | Y | Y | Y | Y | Y | / | Y | N | N | N | N | N | N | Y | N | N | N | Y | N | N | N |
| Razzak et al. | 2019 | Y | P | Y | Y | Y | Y | Y | Y | Y | / | Y | N | N | Y | Y | Y | Y | Y | N | N | / | Y | Y | Y | Y | Y | / | Y | N | N | N | N | N | N | Y | N | N | N | Y | N | N | N |
| Sriramakrishnan et al. | 2019 | Y | P | Y | Y | Y | Y | Y | Y | Y | / | Y | N | N | Y | Y | Y | Y | Y | N | N | / | Y | Y | Y | Y | Y | / | Y | Y | Y | N | N | N | N | Y | Y | N | N | Y | N | N | N |
| Kumar and VijayKumar | 2019 | Y | P | Y | Y | Y | Y | P | N | Y | / | Y | N | N | N | N | N | N | N | N | N | / | Y | Y | Y | Y | Y | Y | Y | N | N | N | N | N | N | Y | N | N | N | Y | N | N | N |
| Mlynarski et al. | 2019 | Y | Y | Y | Y | Y | Y | Y | Y | Y | / | Y | N | Y | Y | Y | Y | Y | Y | N | Y | / | Y | Y | Y | Y | Y | Y | Y | Y | Y | N | Y | N | N | Y | Y | N | Y | Y | N | Y | Y |
| Mallick et al. | 2019 | Y | P | Y | Y | Y | Y | Y | Y | Y | / | Y | N | N | Y | Y | Y | Y | Y | N | Y | / | Y | Y | Y | Y | Y | / | Y | Y | Y | N | Y | N | N | Y | Y | N | N | Y | N | N | N |
| Sun et al. | 2019 | Y | Y | Y | Y | Y | Y | Y | Y | Y | / | Y | N | N | Y | Y | Y | Y | Y | N | N | / | Y | Y | Y | N | Y | / | Y | Y | N | N | N | N | N | Y | Y | N | N | Y | Y | N | Y |
| Janardhanaprabhu and Malathi | 2019 | Y | P | Y | Y | Y | Y | N | N | Y | / | Y | N | N | N | N | N | N | N | N | N | / | Y | Y | Y | Y | Y | / | Y | N | N | N | N | N | N | Y | N | N | N | Y | N | N | N |
| Kebir et al. | 2019 | Y | Y | Y | Y | Y | Y | Y | Y | Y | / | Y | N | N | Y | Y | Y | Y | Y | N | N | / | Y | Y | Y | Y | Y | / | Y | Y | N | N | N | N | N | Y | Y | N | N | Y | N | N | N |
| Meghana S et al. | 2019 | Y | P | Y | Y | Y | Y | N | N | Y | / | Y | N | N | N | N | N | N | N | N | N | / | Y | Y | Y | Y | Y | / | Y | N | N | N | N | N | N | Y | N | N | N | Y | N | N | N |
| Iqbal et al. | 2019 | Y | Y | Y | Y | Y | Y | Y | Y | Y | / | Y | N | N | Y | Y | Y | Y | Y | N | Y | / | Y | Y | Y | Y | Y | / | Y | N | Y | N | Y | N | N | Y | N | N | N | Y | N | N | N |
| Alagarsamy et al. | 2019 | Y | Y | Y | Y | Y | Y | Y | N | Y | / | Y | N | N | Y | Y | Y | Y | Y | N | N | / | Y | Y | Y | N | Y | / | Y | Y | N | N | N | N | N | Y | Y | N | N | Y | N | N | N |
| Shivhare et al. | 2019 | Y | Y | Y | Y | Y | Y | Y | Y | Y | / | Y | N | N | Y | Y | Y | Y | Y | N | Y | / | Y | Y | Y | N | Y | / | Y | Y | N | N | N | N | N | Y | Y | N | Y | Y | N | N | N |
| Nema et al. | 2019 | Y | P | Y | Y | Y | Y | Y | Y | Y | / | Y | N | N | Y | Y | Y | Y | Y | N | Y | / | Y | Y | Y | Y | Y | / | Y | N | Y | N | Y | N | N | Y | N | N | N | Y | N | N | N |
| Peng et al. | 2019 | Y | Y | Y | Y | Y | Y | Y | Y | Y | / | Y | N | N | Y | Y | Y | Y | Y | N | Y | / | Y | Y | Y | Y | Y | / | Y | N | Y | N | Y | N | N | Y | N | N | N | Y | Y | N | Y |
| Kalaiselvi et al. | 2019 | Y | Y | Y | Y | Y | Y | Y | Y | Y | / | Y | N | N | Y | Y | Y | Y | Y | N | Y | / | Y | Y | Y | Y | Y | / | Y | N | Y | N | Y | N | N | Y | N | N | N | Y | N | N | N |
| Yang et al. | 2019 | Y | Y | Y | Y | Y | Y | Y | Y | Y | / | Y | N | N | Y | Y | Y | Y | Y | N | Y | / | Y | Y | Y | Y | Y | Y | Y | P | Y | N | Y | N | N | Y | P | N | N | Y | Y | N | Y |
| Virupakshappa and Amarapur | 2019 | Y | P | Y | Y | Y | Y | Y | Y | Y | / | Y | N | N | Y | Y | Y | Y | Y | N | N | / | Y | Y | Y | Y | Y | / | Y | N | N | N | Y | N | N | Y | N | N | N | Y | N | N | Y |
| Wu et al. | 2019 | Y | Y | Y | Y | Y | Y | Y | Y | Y | / | Y | N | N | Y | Y | Y | Y | Y | N | Y | / | Y | Y | Y | Y | Y | / | Y | Y | Y | N | Y | N | N | Y | Y | N | N | Y | Y | N | Y |
| Wang et al. | 2019 | Y | Y | Y | Y | Y | Y | Y | Y | Y | / | Y | N | N | Y | Y | Y | Y | Y | N | Y | / | Y | Y | Y | Y | Y | / | Y | N | Y | N | Y | N | N | Y | N | N | N | Y | Y | N | Y |
| Rehman et al. | 2019 | Y | P | Y | Y | Y | Y | Y | Y | Y | / | Y | N | N | Y | Y | Y | Y | Y | N | Y | / | Y | Y | Y | Y | Y | / | Y | Y | Y | N | Y | N | N | Y | Y | N | Y | Y | N | N | N |
| Kharrat and Neji | 2020 | Y | P | Y | Y | Y | Y | Y | Y | Y | / | Y | N | N | Y | Y | Y | Y | Y | N | Y | / | Y | Y | Y | Y | Y | / | Y | N | Y | N | Y | N | N | Y | N | N | N | Y | N | N | N |
| Rehman et al. | 2020 | Y | Y | Y | Y | Y | Y | Y | Y | Y | / | Y | N | N | Y | Y | Y | Y | Y | N | Y | / | Y | Y | Y | Y | Y | / | Y | N | Y | N | Y | N | N | Y | N | N | N | Y | N | N | Y |
| Khosravanian et al. | 2020 | Y | Y | Y | Y | Y | Y | Y | N | Y | / | Y | N | N | Y | Y | Y | Y | Y | N | N | / | Y | Y | Y | Y | Y | / | Y | N | Y | N | N | N | N | Y | N | N | N | Y | N | N | Y |
| Srinivas and Rao | 2020 | Y | Y | Y | Y | Y | Y | Y | Y | Y | / | Y | N | N | Y | Y | Y | Y | Y | N | Y | / | Y | Y | Y | Y | Y | / | Y | N | Y | N | Y | N | N | Y | N | N | N | Y | N | N | N |
| Yogananda et al. | 2020 | Y | Y | Y | Y | Y | Y | Y | Y | Y | / | Y | N | N | Y | Y | Y | Y | Y | N | Y | / | Y | Y | Y | Y | Y | / | Y | N | Y | N | Y | N | N | Y | N | N | Y | Y | Y | N | Y |
| Zhou et al. | 2020 | Y | P | Y | Y | Y | Y | Y | Y | Y | / | Y | N | N | Y | Y | Y | Y | Y | N | Y | / | Y | Y | Y | Y | Y | Y | Y | Y | Y | Y | Y | N | N | Y | Y | N | N | Y | N | Y | N |
| Kumar et al. | 2020 | Y | P | Y | Y | Y | Y | Y | N | Y | / | Y | N | N | Y | Y | Y | Y | Y | N | Y | / | Y | Y | Y | Y | Y | / | Y | N | N | N | Y | N | N | Y | N | N | N | Y | N | N | Y |
| Chen et al. | 2020 | Y | Y | Y | Y | Y | Y | Y | Y | Y | / | Y | N | N | Y | Y | Y | Y | Y | N | Y | / | Y | Y | Y | Y | Y | Y | Y | P | Y | N | Y | N | N | Y | P | N | Y | Y | Y | N | Y |
| Chen et al. | 2020 | Y | Y | Y | Y | Y | Y | Y | Y | Y | / | Y | N | N | Y | Y | Y | Y | Y | N | N | / | Y | Y | Y | Y | Y | / | Y | Y | Y | N | Y | N | N | Y | Y | N | N | Y | Y | N | Y |
| Tjahyaningtijas et al. | 2020 | Y | Y | Y | Y | Y | Y | Y | Y | Y | / | Y | N | N | Y | Y | Y | Y | Y | N | Y | / | Y | Y | Y | Y | Y | Y | Y | N | N | N | Y | N | N | Y | N | N | N | Y | N | N | Y |
| Khan et al. | 2020 | Y | Y | Y | Y | Y | Y | Y | Y | Y | / | Y | N | N | Y | Y | Y | Y | Y | N | Y | / | Y | Y | Y | Y | Y | Y | Y | Y | Y | N | Y | N | N | Y | Y | N | N | Y | Y | N | Y |
| Zhang et al. | 2020 | Y | P | Y | Y | Y | Y | Y | Y | Y | / | Y | N | N | Y | Y | Y | Y | Y | N | Y | / | Y | Y | Y | Y | Y | / | Y | N | Y | N | Y | N | N | Y | N | N | N | Y | Y | N | Y |
| Zhang et al. | 2020 | Y | P | Y | Y | Y | Y | Y | Y | Y | / | Y | N | N | Y | Y | Y | Y | Y | N | Y | / | Y | Y | Y | Y | Y | / | Y | N | Y | N | Y | N | N | Y | N | N | Y | Y | Y | N | Y |
| Xue et al. | 2020 | Y | Y | Y | Y | Y | Y | Y | Y | Y | / | Y | N | N | Y | Y | Y | Y | Y | N | Y | / | Y | Y | Y | Y | Y | / | Y | Y | Y | N | Y | Y | Y | Y | Y | Y | Y | Y | Y | N | Y |
| Sun et al. | 2020 | Y | Y | Y | Y | Y | Y | Y | Y | Y | / | Y | N | N | Y | Y | Y | Y | Y | N | N | / | Y | Y | Y | Y | Y | / | Y | P | Y | N | Y | N | N | Y | P | N | N | Y | Y | Y | Y |
| Liu et al. | 2020 | Y | Y | Y | Y | Y | Y | Y | Y | Y | / | Y | N | N | Y | Y | Y | Y | Y | N | Y | / | Y | Y | Y | Y | Y | / | Y | N | Y | N | Y | N | N | Y | N | N | Y | Y | Y | N | Y |
| Chaudhary et al. | 2020 | Y | Y | Y | Y | Y | Y | Y | Y | Y | / | Y | N | N | Y | Y | Y | Y | Y | N | Y | / | Y | Y | Y | Y | Y | / | Y | N | N | N | Y | N | N | Y | N | N | N | Y | N | N | N |
| Laukamp et al. | 2020 | Y | Y | Y | Y | Y | Y | Y | Y | Y | / | Y | N | N | Y | Y | Y | Y | Y | N | Y | / | Y | Y | Y | Y | Y | / | Y | Y | Y | N | Y | Y | Y | Y | Y | Y | Y | Y | N | N | N |
| Thiruvenkadam and Perumal | 2016 | Y | Y | Y | Y | Y | Y | Y | Y | Y | / | Y | N | N | Y | Y | Y | Y | Y | N | N | / | Y | Y | Y | N | Y | / | Y | Y | N | N | N | N | N | Y | Y | N | N | Y | N | N | N |
| Gupta et al. | 2020 | Y | P | Y | Y | Y | Y | Y | N | Y | / | Y | N | N | N | N | N | N | N | N | N | / | Y | Y | Y | N | Y | / | Y | N | N | N | N | N | N | Y | N | N | N | Y | N | N | N |
| Yepuganti et al. | 2020 | Y | P | Y | Y | Y | Y | Y | N | N | / | Y | N | N | N | N | N | N | N | N | N | / | Y | Y | Y | N | Y | / | Y | N | N | N | N | N | N | Y | N | N | N | Y | N | N | N |
| Bousabarah et al. | 2020 | Y | Y | Y | Y | Y | Y | Y | Y | Y | / | Y | N | N | P | P | P | P | P | N | Y | / | Y | Y | Y | Y | Y | Y | Y | Y | Y | N | Y | N | N | Y | Y | N | Y | Y | N | N | Y |
| Ejaz et al. | 2020 | Y | Y | Y | Y | Y | Y | Y | Y | Y | / | Y | N | N | Y | Y | Y | Y | Y | N | Y | / | Y | Y | Y | Y | Y | / | Y | N | N | N | Y | N | N | Y | N | N | N | Y | N | N | N |
| Pennig et al. | 2020 | Y | Y | Y | Y | Y | Y | Y | Y | Y | / | Y | N | N | Y | Y | Y | Y | Y | N | Y | / | Y | Y | Y | Y | Y | / | Y | Y | Y | N | Y | N | Y | Y | Y | Y | Y | Y | N | N | N |
| Mohamed et al. | 2020 | Y | Y | Y | Y | Y | Y | Y | N | Y | / | Y | N | N | Y | Y | Y | Y | Y | N | N | / | Y | Y | Y | Y | Y | / | Y | N | N | N | N | N | N | Y | N | N | N | Y | N | N | N |
| Ali et al. | 2020 | Y | Y | Y | Y | Y | Y | Y | Y | Y | / | Y | N | N | Y | Y | Y | Y | Y | N | Y | / | Y | Y | Y | Y | Y | Y | Y | N | Y | N | Y | N | N | Y | N | N | N | Y | N | N | N |
| Katouli and Rahmani | 2020 | Y | P | Y | Y | Y | Y | Y | Y | Y | / | Y | N | N | Y | Y | Y | Y | Y | N | N | / | Y | Y | Y | Y | Y | / | Y | N | Y | N | N | N | N | Y | N | N | N | Y | N | N | N |
| Naceur et al. | 2020 | Y | Y | Y | Y | Y | Y | Y | Y | Y | / | Y | N | N | Y | Y | Y | Y | Y | N | Y | / | Y | Y | Y | Y | Y | / | Y | Y | Y | N | Y | N | N | Y | Y | Y | Y | Y | N | Y | N |
| Sharif et al. | 2020 | Y | P | Y | Y | Y | Y | Y | Y | Y | / | Y | N | N | Y | Y | Y | Y | Y | N | N | / | Y | Y | Y | Y | Y | / | Y | N | Y | N | Y | N | N | Y | N | N | N | Y | N | N | N |
| Aboelenein et al. | 2020 | Y | Y | Y | Y | Y | Y | Y | Y | Y | / | Y | N | N | Y | Y | Y | Y | Y | N | Y | / | Y | Y | Y | Y | Y | / | Y | Y | Y | N | Y | N | N | Y | Y | N | N | Y | N | N | N |
| Zaihani et al. | 2020 | Y | Y | Y | Y | Y | Y | Y | N | Y | / | Y | N | N | Y | Y | Y | Y | Y | N | N | / | Y | Y | Y | N | Y | / | Y | N | N | N | N | N | N | Y | N | N | N | Y | Y | N | Y |
| Hassen et al. | 2020 | Y | Y | Y | Y | Y | Y | Y | Y | Y | / | Y | N | N | Y | Y | Y | Y | Y | N | N | / | Y | Y | Y | Y | Y | / | Y | N | Y | N | N | N | N | Y | N | N | N | Y | N | N | Y |
| Chithra and Dheepa | 2020 | Y | Y | Y | Y | Y | Y | Y | Y | Y | / | Y | N | N | Y | Y | Y | Y | Y | N | N | / | Y | Y | Y | Y | Y | / | Y | N | Y | N | N | N | N | Y | N | N | Y | Y | N | N | N |
| Kao et al. | 2020 | Y | P | Y | Y | Y | Y | Y | Y | Y | / | Y | N | N | Y | Y | Y | Y | Y | N | Y | / | Y | Y | Y | Y | Y | Y | Y | Y | Y | N | Y | N | N | Y | Y | N | N | Y | Y | N | Y |
| Pitchai et al. | 2020 | Y | Y | Y | Y | Y | Y | Y | Y | Y | / | Y | N | N | Y | Y | Y | Y | Y | N | Y | / | Y | Y | Y | Y | Y | / | Y | N | Y | N | Y | N | N | Y | N | N | N | Y | N | N | N |
| Zeineldin et al. | 2020 | Y | Y | Y | Y | Y | Y | Y | Y | Y | / | Y | N | N | Y | Y | Y | Y | Y | N | N | / | Y | Y | Y | Y | Y | / | Y | N | Y | N | Y | N | N | Y | N | N | N | Y | Y | Y | Y |
| Al-qazzaz et al. | 2020 | Y | P | Y | Y | Y | Y | Y | Y | Y | / | Y | N | N | Y | Y | Y | Y | Y | N | Y | / | Y | Y | Y | Y | Y | / | Y | N | Y | N | Y | N | N | Y | N | N | N | Y | N | N | Y |
| Al-qazzaz et al. | 2020 | Y | Y | Y | Y | Y | Y | Y | Y | Y | / | Y | N | N | Y | Y | Y | Y | Y | N | Y | / | Y | Y | Y | Y | Y | Y | Y | N | Y | N | Y | N | N | Y | N | N | Y | Y | N | N | N |
| Sathish and Elango | 2020 | Y | Y | Y | Y | Y | Y | Y | Y | Y | / | Y | N | N | Y | Y | Y | Y | Y | N | Y | / | Y | Y | Y | Y | Y | / | Y | N | Y | N | Y | N | N | Y | N | N | N | Y | N | N | N |
| Banerjee and Mitra | 2020 | Y | Y | Y | Y | Y | Y | Y | Y | Y | / | Y | N | N | Y | Y | Y | Y | Y | N | Y | / | Y | Y | Y | Y | Y | / | Y | N | Y | N | Y | N | N | Y | N | N | N | Y | N | N | N |
| Tripathi et al. | 2020 | Y | Y | Y | Y | Y | Y | Y | Y | Y | / | Y | N | N | Y | Y | Y | Y | Y | N | N | / | Y | Y | Y | Y | Y | / | Y | N | Y | N | Y | N | N | Y | N | N | N | Y | N | N | N |
| Kanniappan et al. | 2020 | Y | Y | Y | Y | Y | Y | Y | N | Y | / | Y | N | N | P | P | P | P | P | N | N | / | Y | Y | Y | N | Y | / | Y | N | Y | N | N | N | N | Y | N | N | N | Y | N | N | Y |
| Debnath et al. | 2020 | Y | Y | Y | Y | Y | Y | Y | Y | Y | / | Y | N | N | Y | Y | Y | Y | Y | N | N | / | Y | Y | Y | N | Y | / | Y | N | Y | N | N | N | N | Y | N | N | N | Y | N | N | N |
| Baid et al. | 2020 | Y | Y | Y | Y | Y | Y | Y | Y | Y | / | Y | N | N | Y | Y | Y | Y | Y | N | Y | / | Y | Y | Y | Y | Y | / | Y | Y | Y | N | Y | N | N | Y | Y | N | Y | Y | Y | N | Y |
| Hu et al. | 2020 | Y | Y | Y | Y | Y | Y | Y | Y | Y | / | Y | N | N | Y | Y | Y | Y | Y | N | Y | / | Y | Y | Y | Y | Y | / | Y | N | Y | N | Y | N | N | Y | N | N | N | Y | N | N | Y |
| Zhou et al. | 2020 | Y | P | Y | Y | Y | Y | Y | Y | Y | / | Y | N | N | Y | Y | Y | Y | Y | N | Y | / | Y | Y | Y | Y | Y | / | Y | P | Y | N | Y | N | N | Y | P | N | N | Y | Y | N | Y |
| Zhou et al. | 2020 | Y | Y | Y | Y | Y | Y | Y | Y | Y | / | Y | N | N | Y | Y | Y | Y | Y | N | Y | / | Y | Y | Y | Y | Y | / | Y | N | Y | N | Y | N | N | Y | N | N | N | Y | Y | N | Y |
| Barzegar and jamzad | 2020 | Y | P | Y | Y | Y | Y | Y | Y | Y | / | Y | N | N | Y | Y | Y | Y | Y | N | Y | / | Y | Y | Y | Y | Y | Y | Y | N | Y | N | Y | N | N | Y | N | N | N | Y | N | N | N |
| Gyorfi et al. | 2021 | Y | Y | Y | Y | Y | Y | Y | Y | Y | / | Y | N | Y | Y | Y | Y | Y | Y | N | Y | / | Y | Y | Y | Y | Y | Y | Y | N | Y | N | Y | N | N | Y | N | Y | Y | Y | Y | N | Y |
| Leva et al. | 2021 | Y | Y | Y | Y | Y | Y | Y | Y | Y | / | Y | N | Y | Y | Y | Y | Y | Y | N | Y | / | Y | Y | Y | Y | Y | / | Y | N | Y | Y | Y | N | N | Y | N | Y | Y | Y | Y | Y | Y |
| Biratu et al. | 2021 | Y | Y | Y | Y | Y | Y | Y | N | Y | / | Y | N | N | Y | Y | Y | Y | Y | N | N | / | Y | Y | Y | Y | Y | / | Y | N | N | N | N | N | N | Y | N | N | N | Y | N | N | Y |
| Rai et al. | 2021 | Y | Y | Y | Y | Y | Y | Y | Y | Y | / | Y | N | N | Y | Y | Y | Y | Y | N | Y | / | Y | Y | Y | Y | Y | / | Y | N | N | N | Y | N | N | Y | N | N | N | Y | N | N | N |
| Mitchell et al. | 2021 | Y | Y | Y | Y | Y | Y | Y | Y | Y | / | Y | N | N | Y | Y | Y | Y | Y | N | Y | / | Y | Y | Y | Y | Y | Y | Y | Y | Y | N | Y | N | Y | Y | Y | N | Y | Y | N | N | N |
| Abirami et al. | 2021 | Y | Y | Y | Y | Y | Y | Y | Y | Y | / | Y | N | N | Y | Y | Y | Y | Y | N | Y | / | Y | Y | Y | Y | Y | / | Y | P | Y | N | Y | N | N | Y | N | N | N | Y | N | N | N |
| Al-Dabagh | 2021 | Y | Y | Y | Y | Y | Y | Y | Y | Y | / | Y | N | N | Y | Y | Y | Y | Y | N | N | / | Y | Y | Y | N | Y | / | Y | N | N | N | N | N | N | Y | N | N | N | Y | N | N | N |
| Sohail et al. | 2021 | Y | P | Y | Y | Y | Y | Y | Y | Y | / | Y | N | N | Y | Y | Y | Y | Y | N | Y | / | Y | Y | Y | Y | Y | / | Y | N | Y | N | Y | N | N | Y | N | N | N | Y | N | N | N |
| Sran et al. | 2021 | Y | Y | Y | Y | Y | Y | Y | N | Y | / | Y | N | N | Y | Y | Y | Y | Y | N | N | / | Y | Y | Y | N | Y | / | Y | Y | N | N | N | N | N | Y | Y | N | N | Y | N | N | N |
| Saxena et al. | 2021 | Y | P | Y | Y | Y | Y | Y | Y | Y | / | Y | N | N | Y | Y | Y | Y | Y | N | Y | / | Y | Y | Y | Y | Y | / | Y | N | Y | N | Y | N | N | Y | N | N | N | Y | N | N | N |
| Takahashi et al. | 2021 | Y | Y | Y | Y | Y | Y | Y | Y | Y | / | Y | N | N | Y | Y | Y | Y | Y | N | Y | / | Y | Y | Y | Y | Y | / | Y | Y | Y | N | Y | Y | Y | Y | Y | Y | Y | Y | Y | N | Y |
| Latif et al. | 2021 | Y | Y | Y | Y | Y | Y | Y | Y | Y | / | Y | N | N | Y | Y | Y | Y | Y | N | Y | / | Y | Y | Y | Y | Y | / | Y | P | Y | N | Y | N | N | Y | P | N | N | Y | Y | N | Y |
| Zhang et al. | 2021 | Y | Y | Y | Y | Y | Y | Y | Y | Y | / | Y | N | N | Y | Y | Y | Y | Y | N | Y | / | Y | Y | Y | Y | Y | / | Y | P | Y | N | Y | N | N | Y | P | N | Y | Y | Y | N | Y |
| Lei et al. | 2021 | Y | Y | Y | Y | Y | Y | Y | Y | Y | / | Y | N | N | Y | Y | Y | Y | Y | N | N | / | Y | Y | Y | Y | Y | / | Y | N | Y | N | N | N | N | Y | N | N | N | Y | N | N | Y |
| Wang et al. | 2021 | Y | Y | Y | Y | Y | Y | Y | Y | Y | / | Y | N | N | Y | Y | Y | Y | Y | N | N | / | Y | Y | Y | Y | Y | Y | Y | P | Y | N | Y | N | N | Y | P | N | N | Y | N | N | Y |
| Zhao et al. | 2021 | Y | Y | Y | Y | Y | Y | Y | Y | Y | / | Y | N | N | Y | Y | Y | Y | Y | N | Y | / | Y | Y | Y | Y | Y | / | Y | N | Y | N | Y | N | N | Y | N | N | N | Y | N | N | Y |
| Cao et al. | 2021 | Y | Y | Y | Y | Y | Y | Y | Y | Y | / | Y | N | N | Y | Y | Y | Y | Y | N | Y | / | Y | Y | Y | Y | Y | / | Y | N | Y | N | Y | N | N | Y | N | N | N | Y | N | N | N |
| Barzegar and jamzad | 2021 | Y | P | Y | Y | Y | Y | Y | Y | Y | / | Y | N | N | Y | Y | Y | Y | Y | N | Y | / | Y | Y | Y | Y | Y | / | Y | N | Y | N | Y | N | N | Y | N | N | N | Y | N | N | N |
| Y=Yes; N=No; “/”=Not Applicable; 1=Identification as a study of AI methodology, specifying the category of technology used (e.g., deep learning); 2=Structured summary of study design, methods, results, and conclusions; 3=Scientific and clinical background, including the intended use and clinical role of the AI approach; 4=Study objectives and hypotheses; 5=Prospective or retrospective study; 6=Study goal, such as model creation, exploratory study, feasibility study, non-inferiority trial; 7=Data sources; 8=Eligibility criteria: how, where, and when potentially eligible participants or studies were identified; 9=Data pre-processing steps ; 10=Selection of data subsets, if applicable; 11=Definitions of data elements, with references to Common Data Elements; 12=De-identification methods; 13=How missing data were handled; 14=Definition of ground truth reference standard, in sufficient detail to allow replication; 15=Rationale for choosing the reference standard (if alternatives exist); 16=Source of ground-truth annotations; qualifications and preparation of annotators; 17=Annotation tools; 18=Measurement of inter- and intrarater variability; methods to mitigate variability and/or resolve discrepancies; 19=Intended sample size and how it was determined; 20=How data were assigned to partitions; specify proportions; 21=Level at which partitions are disjoint (e.g., image, study, patient, institution); 22=Detailed description of model, including inputs, outputs, all intermediate layers and connections; 23=Software libraries, frameworks, and packages; 24=Initialization of model parameters (e.g., randomization, transfer learning); 25=Details of training approach, including data augmentation, hyperparameters, number of models trained; 26=Method of selecting the final model; 27=Ensembling techniques, if applicable; 28=Metrics of model performance; 29=Statistical measures of significance and uncertainty (e.g., confidence intervals); 30=Robustness or sensitivity analysis; 31=Methods for explainability or interpretability (e.g., saliency maps), and how they were validated; 32=Validation or testing on external data; 33=Flow of participants or cases, using a diagram to indicate inclusion and exclusion; 34=Demographic and clinical characteristics of cases in each partition; 35=Performance metrics for optimal model(s) on all data partitions; 36=Estimates of diagnostic accuracy and their precision (such as 95% confidence intervals); 37=Failure analysis of incorrectly classified cases; 38=Study limitations, including potential bias, statistical uncertainty, and generalizability; 39=Implications for practice, including the intended use and/or clinical role; 40=Registration number and name of registry; 41=Where the full study protocol can be accessed; 42=Sources of funding and other support; role of funders | | | | | | | | | | | | | | | | | | | | | | | | | | | | | | | | | | | | | | | | | | | |

# Supplementary table 7: Detailed CLAIM quality reporting assessment for detection studies

| **Author** | **Year** | **1** | **2** | **3** | **4** | **5** | **6** | **7** | **8** | **9** | **10** | **11** | **12** | **13** | **14** | **15** | **16** | **17** | **18** | **19** | **20** | **21** | **22** | **23** | **24** | **25** | **26** | **27** | **28** | **29** | **30** | **31** | **32** | **33** | **34** | **35** | **36** | **37** | **38** | **39** | **40** | **41** | **42** |
| --- | --- | --- | --- | --- | --- | --- | --- | --- | --- | --- | --- | --- | --- | --- | --- | --- | --- | --- | --- | --- | --- | --- | --- | --- | --- | --- | --- | --- | --- | --- | --- | --- | --- | --- | --- | --- | --- | --- | --- | --- | --- | --- | --- |
| Jayachandran and Dhanasekaran | 2012 | Y | Y | Y | Y | Y | Y | Y | Y | Y | / | Y | N | N | N | N | N | N | N | N | Y | / | Y | Y | Y | Y | Y | / | Y | N | N | N | Y | N | N | Y | N | N | N | Y | N | N | N |
| Farjam et al. | 2012 | Y | Y | Y | Y | Y | Y | Y | Y | Y | / | Y | N | N | Y | Y | Y | Y | Y | N | Y | / | Y | Y | Y | Y | Y | / | Y | N | Y | N | Y | N | N | Y | N | Y | Y | Y | Y | N | Y |
| Jayachandran and Dhanasekaran | 2013 | Y | Y | Y | Y | Y | Y | N | Y | Y | / | Y | N | N | Y | Y | Y | Y | Y | N | Y | / | Y | Y | Y | Y | Y | / | Y | N | N | N | Y | N | N | Y | N | N | N | Y | N | N | N |
| Dvorák et al. | 2013 | Y | Y | Y | Y | Y | Y | Y | N | Y | / | Y | N | N | N | N | N | N | N | N | Y | / | Y | Y | Y | Y | Y | / | Y | N | Y | N | Y | N | N | Y | N | Y | N | Y | N | N | Y |
| Dahshan et al. | 2014 | Y | Y | Y | Y | Y | Y | Y | Y | Y | / | Y | N | N | Y | Y | Y | Y | Y | N | Y | / | Y | Y | Y | Y | Y | / | Y | N | N | N | Y | N | N | Y | N | N | Y | Y | N | N | N |
| Bahadure et al. | 2015 | Y | Y | Y | Y | Y | Y | Y | N | Y | / | Y | N | N | Y | Y | Y | Y | Y | N | N | / | Y | Y | Y | N | Y | / | Y | N | N | N | N | N | N | Y | N | N | N | Y | N | N | N |
| Helen and Kamaraj | 2015 | Y | Y | Y | Y | Y | Y | Y | N | N | / | Y | N | N | N | N | N | N | N | N | Y | / | Y | Y | Y | Y | Y | / | Y | Y | N | N | N | N | N | Y | N | N | N | Y | N | N | N |
| Thirumurugan et al. | 2016 | Y | Y | Y | Y | Y | Y | Y | Y | Y | / | Y | N | N | Y | Y | Y | Y | Y | N | N | / | Y | Y | Y | N | Y | / | Y | N | Y | N | Y | N | N | Y | N | N | N | Y | N | N | N |
| Banerjee et al. | 2016 | Y | Y | Y | Y | Y | Y | Y | Y | Y | / | Y | N | N | Y | Y | Y | Y | Y | N | Y | / | Y | Y | Y | Y | Y | / | Y | Y | Y | N | N | N | N | Y | Y | N | N | Y | N | N | N |
| Amin et al. | 2017 | Y | Y | Y | Y | Y | Y | Y | Y | Y | / | Y | N | N | Y | Y | Y | Y | Y | N | Y | / | Y | Y | Y | Y | Y | / | Y | N | Y | N | Y | N | P | Y | N | N | N | Y | N | N | N |
| Gupta and Khanna | 2017 | Y | Y | Y | Y | Y | Y | Y | Y | Y | / | Y | N | N | Y | Y | Y | Y | Y | N | Y | / | Y | Y | Y | Y | Y | / | Y | Y | Y | N | Y | N | N | Y | Y | N | N | Y | N | N | N |
| Anitha and Raja | 2017 | Y | P | Y | Y | Y | Y | Y | N | Y | / | Y | N | N | Y | Y | Y | Y | Y | N | N | / | Y | Y | Y | N | Y | Y | Y | N | Y | N | Y | N | N | Y | N | N | N | Y | N | N | N |
| Lahmiri | 2017 | Y | Y | Y | Y | Y | Y | Y | Y | Y | / | Y | N | N | Y | Y | Y | Y | Y | N | Y | / | Y | Y | Y | Y | Y | / | Y | Y | Y | N | Y | N | N | Y | Y | N | N | Y | N | N | N |
| Deepa and Emmanuel | 2018 | Y | P | Y | Y | Y | Y | Y | Y | Y | / | Y | N | N | Y | Y | Y | Y | Y | N | Y | / | Y | Y | Y | Y | Y | / | Y | N | Y | N | Y | N | N | Y | N | N | N | Y | N | N | N |
| Selvapandian and Manivannan | 2018 | Y | Y | Y | Y | Y | Y | Y | N | Y | / | Y | N | N | Y | Y | Y | Y | Y | N | Y | / | Y | Y | Y | Y | Y | / | Y | N | Y | N | Y | N | N | Y | N | N | N | Y | N | N | N |
| Abd-Ellah et al. | 2018 | Y | Y | Y | Y | Y | Y | Y | Y | Y | / | Y | N | N | Y | Y | Y | Y | Y | N | Y | / | Y | Y | Y | Y | Y | / | Y | N | N | N | Y | N | N | Y | N | N | N | Y | N | N | N |
| Arunkumar et al. | 2018 | Y | Y | Y | Y | Y | Y | N | N | Y | / | Y | N | N | N | N | N | N | N | N | Y | / | Y | Y | Y | Y | Y | / | Y | N | Y | N | Y | N | N | Y | N | N | N | Y | N | N | N |
| Edalati-rad and Mosleh | 2019 | Y | Y | Y | Y | Y | Y | Y | Y | Y | / | Y | N | N | Y | Y | Y | Y | Y | N | Y | / | Y | Y | Y | Y | Y | / | Y | N | N | N | Y | N | N | Y | N | N | N | Y | N | N | N |
| Song et al. | 2019 | Y | Y | Y | Y | Y | Y | Y | Y | Y | / | Y | N | N | N | N | N | N | N | N | Y | / | Y | Y | Y | Y | Y | / | Y | Y | Y | N | Y | N | N | Y | N | N | Y | Y | N | N | N |
| Johnpeter and Ponnuchamy | 2019 | Y | Y | Y | Y | Y | Y | Y | Y | Y | / | Y | N | N | Y | Y | Y | Y | Y | N | N | / | Y | Y | Y | Y | Y | / | Y | N | N | N | N | N | N | Y | N | N | N | Y | N | N | N |
| Amin et al. | 2019 | Y | Y | Y | Y | Y | Y | Y | Y | Y | / | Y | N | N | Y | Y | Y | Y | Y | N | Y | / | Y | Y | Y | Y | Y | Y | Y | N | Y | N | Y | N | P | Y | N | N | N | Y | N | N | N |
| Alam et al. | 2019 | Y | P | Y | Y | Y | Y | N | N | N | / | Y | N | N | N | N | N | N | N | N | N | / | Y | Y | Y | N | Y | / | Y | N | N | N | N | N | N | Y | N | N | N | Y | N | N | Y |
| Atici et al. | 2019 | Y | Y | Y | Y | Y | Y | Y | Y | Y | / | Y | N | N | Y | Y | Y | Y | Y | N | Y | / | Y | Y | Y | Y | Y | / | Y | N | Y | N | Y | N | N | Y | N | N | Y | Y | N | N | N |
| Sriramakrishnan et al. | 2019 | Y | P | Y | Y | Y | Y | Y | Y | Y | / | Y | N | N | Y | Y | Y | Y | Y | N | N | / | Y | Y | Y | Y | Y | / | Y | Y | Y | N | Y | N | N | Y | Y | N | N | Y | N | N | N |
| Kebir et al. | 2019 | Y | Y | Y | Y | Y | Y | Y | Y | Y | / | Y | N | N | Y | Y | Y | Y | Y | N | N | / | Y | Y | Y | Y | Y | / | Y | Y | N | N | N | N | N | Y | Y | N | N | Y | N | N | N |
| Kalaiselvi et al. | 2019 | Y | Y | Y | Y | Y | Y | Y | Y | Y | / | Y | N | N | Y | Y | Y | Y | Y | N | Y | / | Y | Y | Y | Y | Y | / | Y | N | Y | N | Y | N | N | Y | N | N | N | Y | N | N | N |
| Çinar and Yildirim | 2020 | Y | Y | Y | Y | Y | Y | Y | Y | Y | / | Y | N | N | N | N | N | N | N | N | N | / | Y | Y | Y | Y | Y | / | Y | N | N | N | N | N | N | Y | N | N | N | Y | N | N | Y |
| Devanathan and Venkatachalapathy | 2020 | Y | Y | Y | Y | Y | Y | Y | Y | Y | / | Y | N | N | N | N | N | N | N | N | N | / | Y | Y | Y | Y | Y | / | Y | N | N | N | N | N | N | Y | N | N | N | Y | N | N | Y |
| Dikici et al. | 2020 | Y | Y | Y | Y | Y | Y | Y | Y | Y | / | Y | N | N | Y | Y | Y | Y | Y | N | Y | / | Y | Y | Y | Y | Y | / | Y | Y | Y | N | Y | N | N | Y | Y | N | N | Y | N | N | N |
| Dheepa and Chithra | 2020 | Y | Y | Y | Y | Y | Y | Y | N | Y | / | Y | N | N | Y | Y | Y | Y | Y | N | N | / | Y | Y | Y | Y | Y | / | Y | N | N | N | N | N | N | Y | N | N | N | Y | N | N | N |
| Gurunathan and Krishnan | 2020 | Y | P | Y | Y | Y | Y | Y | N | Y | / | Y | N | N | Y | Y | Y | Y | Y | N | Y | / | Y | Y | Y | Y | Y | / | Y | N | Y | N | Y | N | N | Y | N | N | N | Y | N | N | N |
| Wang et al. | 2020 | Y | Y | Y | Y | Y | Y | Y | Y | Y | / | Y | N | N | Y | Y | Y | Y | Y | N | Y | / | Y | Y | Y | Y | Y | / | Y | Y | Y | N | Y | N | N | Y | Y | N | N | Y | N | N | Y |
| Kesav and Rajini | 2020 | Y | Y | Y | Y | Y | Y | Y | Y | Y | / | Y | N | N | Y | Y | Y | Y | Y | N | Y | / | Y | Y | Y | Y | Y | Y | Y | N | Y | N | Y | N | N | Y | N | N | N | Y | N | N | N |
| Pennig et al. | 2020 | Y | Y | Y | Y | Y | Y | Y | Y | Y | / | Y | N | N | Y | Y | Y | Y | Y | N | N | / | Y | Y | Y | Y | Y | / | Y | Y | Y | N | Y | N | Y | Y | Y | Y | Y | Y | N | N | N |
| Murali and Meena | 2020 | Y | P | Y | Y | Y | Y | N | N | Y | / | Y | N | N | N | N | N | N | N | N | N | / | Y | Y | Y | N | Y | / | Y | N | N | N | N | N | N | Y | N | Y | N | Y | N | N | N |
| Sathish and Elango | 2020 | Y | Y | Y | Y | Y | Y | Y | Y | Y | / | Y | N | N | Y | Y | Y | Y | Y | N | Y | / | Y | Y | Y | Y | Y | / | Y | N | Y | N | Y | N | N | Y | N | N | N | Y | N | N | N |
| Kaur and Ghandi | 2020 | Y | Y | Y | Y | Y | Y | Y | Y | Y | / | Y | N | N | Y | Y | Y | Y | Y | N | Y | / | Y | Y | Y | Y | Y | / | Y | Y | Y | N | Y | N | N | Y | Y | N | Y | Y | N | N | N |
| Thangarajan and Chokkalingam | 2020 | Y | P | Y | Y | Y | Y | Y | N | Y | / | Y | N | N | N | N | N | N | N | N | Y | / | Y | Y | Y | Y | Y | / | Y | N | Y | N | Y | N | N | Y | N | N | N | Y | N | N | N |
| Kalaiselvi et al. | 2020 | Y | N | Y | Y | Y | Y | Y | N | Y | / | Y | N | N | Y | Y | Y | Y | Y | N | N | / | Y | Y | Y | Y | Y | / | Y | N | Y | N | Y | N | N | Y | N | N | N | Y | N | N | N |
| Rajinikanth et al. | 2020 | Y | Y | Y | Y | Y | Y | Y | Y | Y | / | Y | N | N | Y | Y | Y | Y | Y | N | Y | / | Y | Y | Y | Y | Y | / | Y | N | Y | N | Y | N | N | Y | N | N | Y | Y | Y | N | Y |
| Huang et al. | 2020 | Y | Y | Y | Y | Y | Y | Y | Y | Y | / | Y | N | N | Y | Y | Y | Y | Y | N | Y | / | Y | Y | Y | Y | Y | / | Y | N | Y | N | Y | N | N | Y | N | Y | N | Y | Y | N | Y |
| Chen et al. | 2021 | Y | Y | Y | Y | Y | Y | Y | Y | Y | / | Y | N | N | Y | Y | Y | Y | Y | N | Y | / | Y | Y | Y | Y | Y | / | Y | N | Y | N | Y | N | N | Y | N | N | Y | Y | Y | N | Y |
| Rai et al. | 2021 | Y | Y | Y | Y | Y | Y | Y | Y | Y | / | Y | N | N | Y | Y | Y | Y | Y | N | Y | / | Y | Y | Y | Y | Y | / | Y | N | Y | N | Y | N | N | Y | N | N | N | Y | N | N | N |
| Patil and hamde | 2021 | Y | P | Y | Y | Y | Y | Y | Y | Y | / | Y | N | N | Y | Y | Y | Y | Y | N | Y | / | Y | Y | Y | Y | Y | / | Y | N | Y | N | Y | N | N | Y | N | N | N | Y | N | N | N |
| Simaiya et al. | 2021 | Y | P | Y | Y | Y | Y | Y | N | Y | / | Y | N | N | N | N | N | N | N | N | Y | / | Y | Y | Y | Y | Y | / | Y | N | Y | N | Y | N | N | Y | N | N | N | Y | N | N | N |
| Tejas P and Padma | 2021 | Y | P | Y | Y | Y | Y | Y | N | Y | / | Y | N | N | N | N | N | N | N | N | N | / | Y | Y | Y | Y | Y | / | Y | N | N | N | N | N | N | Y | N | N | N | Y | N | N | N |
| Y=Yes; N=No; “/”=Not Applicable; 1=Identification as a study of AI methodology, specifying the category of technology used (e.g., deep learning); 2=Structured summary of study design, methods, results, and conclusions; 3=Scientific and clinical background, including the intended use and clinical role of the AI approach; 4=Study objectives and hypotheses; 5=Prospective or retrospective study; 6=Study goal, such as model creation, exploratory study, feasibility study, non-inferiority trial; 7=Data sources; 8=Eligibility criteria: how, where, and when potentially eligible participants or studies were identified; 9=Data pre-processing steps ; 10=Selection of data subsets, if applicable; 11=Definitions of data elements, with references to Common Data Elements; 12=De-identification methods; 13=How missing data were handled; 14=Definition of ground truth reference standard, in sufficient detail to allow replication; 15=Rationale for choosing the reference standard (if alternatives exist); 16=Source of ground-truth annotations; qualifications and preparation of annotators; 17=Annotation tools; 18=Measurement of inter- and intrarater variability; methods to mitigate variability and/or resolve discrepancies; 19=Intended sample size and how it was determined; 20=How data were assigned to partitions; specify proportions; 21=Level at which partitions are disjoint (e.g., image, study, patient, institution); 22=Detailed description of model, including inputs, outputs, all intermediate layers and connections; 23=Software libraries, frameworks, and packages; 24=Initialization of model parameters (e.g., randomization, transfer learning); 25=Details of training approach, including data augmentation, hyperparameters, number of models trained; 26=Method of selecting the final model; 27=Ensembling techniques, if applicable; 28=Metrics of model performance; 29=Statistical measures of significance and uncertainty (e.g., confidence intervals); 30=Robustness or sensitivity analysis; 31=Methods for explainability or interpretability (e.g., saliency maps), and how they were validated; 32=Validation or testing on external data; 33=Flow of participants or cases, using a diagram to indicate inclusion and exclusion; 34=Demographic and clinical characteristics of cases in each partition; 35=Performance metrics for optimal model(s) on all data partitions; 36=Estimates of diagnostic accuracy and their precision (such as 95% confidence intervals); 37=Failure analysis of incorrectly classified cases; 38=Study limitations, including potential bias, statistical uncertainty, and generalizability; 39=Implications for practice, including the intended use and/or clinical role; 40=Registration number and name of registry; 41=Where the full study protocol can be accessed; 42=Sources of funding and other support; role of funders | | | | | | | | | | | | | | | | | | | | | | | | | | | | | | | | | | | | | | | | | | | |

# Supplementary table 8: Available source codes for methods used in included studies

| **Author** | **Link** |
| --- | --- |
| Sanjuàn et al. | <https://www.fil.ion.ucl.ac.uk/spm/ext/> |
| Leva et al. | <https://github.com/IAmSuyogJadhav/3d-mri-brain-tumor-segmentation-using-autoencoder-regularization> |
| Zhou et al. | <https://github.com/chenhong-zhou/OM-Net> |
| Sun et al. | <https://github.com/JalexDooo/BrainstormTS> |
| Kamnitsas et al. | <https://github.com/deepmedic/deepmedic> |
| Havaei et al. | <https://bitbucket.org/vitalab/vitalabai_public/src/44a2443772267c39d2cb8f79500d6c49163af234/VITALabAI/model/brats/?at=master> |
| Naceur et al. | <https://github.com/MostefaBen> |
| Tustison et al. | <https://github.com/ntustison/BRATS2013> |
| Mlynarski et al. | <https://github.com/PawelMlynarski> |
| Zeineldin et al. | <https://github.com/razeineldin/DeepSeg/> |

# Supplementary table 9: Detailed QUADAS-2 Risk of Bias and Applicability for segmentation studies

| **Author** | **Year** | **Overall domain assessement bias - patient selection** | **Overall domain assessement applicability - patient selection** | **Overall domain assessement bias - index test** | **Overall domain assessement applicability - index test** | **Overall domain assessement bias - reference standard** | **Overall domain assessement applicability - reference standard** | **Overall domain assessement bias - flow and timing** | **Overall judgement of risk of bias** | **Overall judgement of applicability** |
| --- | --- | --- | --- | --- | --- | --- | --- | --- | --- | --- |
| Fletcher-Heath et al. | 2001 | Low | Low | Low | Low | Unclear | Low | Low | Unclear | Low |
| Kaus et al. | 2001 | Low | Low | Low | High | Low | Low | Low | Low | High |
| Prastawa et al. | 2004 | Low | Low | Low | High | Low | Low | Low | Low | High |
| Xie et al. | 2005 | Unclear | Low | Low | High | Low | Low | Low | Unclear | High |
| Corso et al. | 2008 | Low | Low | Low | Low | Low | Low | Low | Low | Low |
| Nie et al. | 2009 | Low | Low | Low | High | Low | Low | Low | Low | High |
| Taheri et al. | 2010 | Unclear | Low | Low | High | High | Low | Low | High | High |
| Hsieh et al. | 2011 | Low | Low | Low | High | Unclear | Low | Low | Unclear | High |
| Hamamci et al. | 2012 | Low | Low | Low | High | Low | Low | Low | Low | High |
| Sanjuàn et al. | 2013 | Low | Low | Low | High | Low | Low | Low | Low | High |
| Jiang et al. | 2013 | Low | Low | Low | High | Low | Low | Low | Low | High |
| Wu et al. | 2013 | Low | Low | Low | Low | Unclear | Low | Low | Unclear | Low |
| Kharrat et al. | 2014 | Unclear | Low | Low | Low | High | Low | Low | High | Low |
| Tustison et al. | 2014 | Low | Low | Low | Low | Low | Low | Low | Low | Low |
| Wu et al. | 2014 | Low | Low | Low | Low | Low | Low | Low | Low | Low |
| Demirhan et al | 2015 | Low | Low | Low | High | High | Low | Low | High | High |
| Abdel-Maksoud et al. | 2015 | Unclear | Low | Low | High | Low | Low | Low | Unclear | High |
| Ali et al. | 2015 | Unclear | Low | Low | High | Low | Low | Low | Unclear | High |
| Njeh et al. | 2015 | Low | Low | Low | Low | Low | Low | Low | Low | Low |
| Albarracín et al. | 2015 | Low | Low | Low | Low | Low | Low | Low | Low | Low |
| Bahadure et al. | 2015 | Low | Low | Low | High | Low | Low | Low | Low | High |
| Nabizadeh and Kubat | 2015 | Low | Low | Low | Low | Low | Low | Low | Low | Low |
| Dvorák et al. | 2015 | Unclear | Low | Low | Low | Low | Low | Low | Unclear | Low |
| Szwarc et al. | 2015 | Low | Low | Low | High | Low | Low | Low | Low | High |
| Steed et al. | 2015 | Low | Low | Low | Low | Low | Low | Low | Low | Low |
| Zhan et al. | 2015 | Unclear | Low | Low | High | Low | Low | Low | Unclear | High |
| Akkus et al. | 2015 | Low | Low | Low | Low | Low | Low | Low | Low | Low |
| Hasan et al. | 2016 | Low | Low | Low | Low | Unclear | Low | Low | Unclear | Low |
| Mbuyamba et al. | 2016 | Unclear | Low | Low | High | Low | Low | Low | Unclear | High |
| Vishnuvarthanan et al. | 2016 | Unclear | Low | Low | High | Unclear | Low | Low | Unclear | High |
| Thiruvenkadam and Nagarajan | 2020 | Low | Low | Low | High | Low | Low | Low | Low | High |
| Zhao and Jia | 2016 | Low | Low | Low | High | Low | Low | Low | Low | High |
| Cordier et al. | 2016 | Low | Low | Low | Low | Low | Low | Low | Low | Low |
| Pereira et al. | 2016 | Low | Low | Low | Low | Low | Low | Low | Low | Low |
| Koley et al. | 2016 | Low | Low | Low | Low | High | Low | Low | High | Low |
| Liu et al. | 2016 | Low | Low | Low | High | Low | Low | Low | Low | High |
| Li et al. | 2016 | Low | Low | Low | Low | Low | Low | Low | Low | Low |
| Vishnuvarthanan et al. | 2017 | Unclear | Low | Low | High | Low | Low | Low | Unclear | High |
| Kamnitsas et al. | 2017 | Low | Low | Low | Low | Low | Low | Low | Low | Low |
| Havaei et al. | 2017 | Low | Low | Low | Low | Low | Low | Low | Low | Low |
| Soltaninejad et al. | 2017 | Low | Low | Low | Low | Low | Low | Low | Low | Low |
| Gupta and Khanna | 2017 | Low | Low | Low | Low | Low | Low | Low | Low | Low |
| Anitha and Raja | 2017 | Low | Low | Low | High | Low | Low | Low | Low | High |
| Cui et al. | 2017 | Low | Low | Low | Low | Low | Low | Low | Low | Low |
| Sasikanth and Kumar | 2017 | Unclear | Low | Low | Low | Low | Low | Low | Unclear | Low |
| Imtiaz et al. | 2017 | Low | Low | Low | Low | Low | Low | Low | Low | Low |
| Kaur et al. | 2017 | Low | Low | Low | Low | Unclear | Low | Low | Unclear | Low |
| Rajinikanth et al. | 2017 | High | Low | Low | Low | Low | Low | Low | High | Low |
| Rajinikanth et al. | 2017 | High | Low | Low | Low | Low | Low | Low | Low | Low |
| Liu et al. | 2017 | Low | Low | Low | Low | Low | Low | Low | Low | Low |
| Zhuge et al. | 2017 | Low | Low | Low | Low | Low | Low | Low | Low | Low |
| Li et al. | 2017 | Low | Low | Low | Low | Low | Low | Low | Low | Low |
| Selvapandian and Manivannan | 2018 | Low | Low | Low | Low | Low | Low | Low | Low | Low |
| Raju et al. | 2018 | Unclear | Low | Low | Low | Low | Low | Low | Unclear | Low |
| Essadike et al. | 2018 | Unclear | Low | Low | High | Low | Low | Low | Unclear | High |
| Kermi et al. | 2018 | Low | Low | Low | High | Low | Low | Low | Low | High |
| Pinto et al. | 2018 | Low | Low | Low | Low | Low | Low | Low | Low | Low |
| Narayanan et al. | 2018 | Unclear | Low | Low | High | Low | Low | Low | Unclear | High |
| Ma et al. | 2018 | Low | Low | Low | Low | Low | Low | Low | Low | Low |
| Amin et al. | 2018 | Low | Low | Low | Low | Low | Low | Low | Low | Low |
| Tong et al. | 2018 | Low | Low | Low | Low | Low | Low | Low | Low | Low |
| Laukamp et al. | 2018 | Low | Low | Low | Low | Low | Low | Low | Low | Low |
| Lim and Mandava | 2018 | Low | Low | Low | High | Low | Low | Low | Low | High |
| Szilágyi et al. | 2018 | Low | Low | Low | High | Low | Low | Low | Low | High |
| Abd-Ellah et al. | 2018 | Low | Low | Low | Low | Low | Low | Low | Low | Low |
| Angulakshmi et al. | 2018 | Low | Low | Low | High | Low | Low | Low | Low | High |
| Perkuhn et al. | 2018 | Low | Low | Low | Low | Low | Low | Low | Low | Low |
| Soltaninejad et al. | 2018 | Low | Low | Low | Low | Low | Low | Low | Low | Low |
| Naceur et al. | 2018 | Low | Low | Low | High | Low | Low | Low | Low | High |
| Charron et al. | 2018 | Low | Low | Low | Low | Low | Low | Low | Low | Low |
| Li et al. | 2018 | Low | Low | Low | Low | Low | Low | Low | Low | Low |
| Zaouche et al. | 2018 | Unclear | Low | Low | High | Low | Low | Low | Unclear | High |
| Hussain et al. | 2018 | Low | Low | Low | Low | Low | Low | Low | Low | Low |
| Iqbal et al. | 2018 | Low | Low | Low | Low | Low | Low | Low | Low | Low |
| Cui et al. | 2018 | Low | Low | Low | Low | Low | Low | Low | Low | Low |
| Bonte et al. | 2018 | Low | Low | Low | Low | Low | Low | Low | Low | Low |
| Zhan et al. | 2018 | Low | Low | Low | High | Low | Low | Low | Low | High |
| Virupakshappa and Basavaraj | 2018 | Low | Low | Low | Low | Low | Low | Low | Low | Low |
| Zhao et al. | 2018 | Low | Low | Low | Low | Low | Low | Low | Low | Low |
| Zhao et al. | 2018 | Low | Low | Low | High | Low | Low | Low | Low | High |
| Jijja and Rai | 2019 | Unclear | Low | Low | High | High | Low | Low | High | High |
| Natarajan and Kumarasamy | 2019 | Unclear | Low | Low | High | High | Low | Low | High | High |
| Chaudhari and Kulkarni | 2019 | Unclear | Low | Low | Low | Low | Low | Low | Unclear | Low |
| Hachemi et al. | 2019 | Unclear | Low | Low | High | Low | Low | Low | Unclear | High |
| Sheela and Suganthi | 2019 | Low | Low | Low | High | Low | Low | Low | Low | High |
| Zhang et al. | 2019 | Unclear | Low | Low | High | Low | Low | Low | Unclear | High |
| Grøvik et al. | 2019 | Low | Low | Low | Low | Low | Low | Low | Low | Low |
| Eltayeb et al. | 2019 | Unclear | Low | Low | High | Low | Low | Low | Unclear | High |
| Nagarathinam and Ponnuchamy | 2019 | Unclear | Low | Low | Low | Low | Low | Low | Unclear | Low |
| Wang et al. | 2019 | Low | Low | Low | Low | Low | Low | Low | Low | Low |
| Li et al. | 2019 | Low | Low | Low | Low | Low | Low | Low | Low | Low |
| Amin et al. | 2019 | Low | Low | Low | Low | Low | Low | Low | Low | Low |
| Sun et al. | 2019 | Low | Low | Low | Low | Low | Low | Low | Low | Low |
| Chang et al. | 2019 | Low | Low | Low | Low | Low | Low | Low | Low | Low |
| Tong et al. | 2019 | Low | Low | Low | Low | Low | Low | Low | Low | Low |
| Shapey et al. | 2019 | Low | Low | Low | Low | Low | Low | Low | Low | Low |
| Zhao et al. | 2019 | Low | Low | Low | Low | Low | Low | Low | Low | Low |
| Dogra et al. | 2019 | Unclear | Low | Low | High | Low | Low | Low | Unclear | High |
| Hu et al. | 2019 | Low | Low | Low | Low | Low | Low | Low | Low | Low |
| Na et al. | 2019 | Low | Low | Low | Low | Low | Low | Low | Low | Low |
| Wang et al. | 2019 | High | Low | Low | High | Low | Low | Low | High | High |
| Thaha et al. | 2019 | Low | Low | Low | High | Low | Low | Low | Low | High |
| Razzak et al. | 2019 | Low | Low | Low | High | Low | Low | Low | Low | High |
| Sriramakrishnan et al. | 2019 | Low | Low | Low | High | Low | Low | Low | Low | High |
| Kumar and VijayKumar | 2019 | Unclear | Low | Low | High | High | Low | Low | High | High |
| Mlynarski et al. | 2019 | Low | Low | Low | Low | Low | Low | Low | Low | Low |
| Mallick et al. | 2019 | Low | Low | Low | Low | Low | Low | Low | Low | Low |
| Sun et al. | 2019 | Low | Low | Low | High | Low | Low | Low | Low | High |
| Janardhanaprabhu and Malathi | 2019 | Unclear | Low | Low | High | High | Low | Low | High | High |
| Kebir et al. | 2019 | Low | Low | Low | High | Low | Low | Low | Low | High |
| Meghana S et al. | 2019 | Unclear | Low | Low | High | High | Low | Low | High | High |
| Iqbal et al. | 2019 | Low | Low | Low | Low | Low | Low | Low | Low | Low |
| Alagarsamy et al. | 2019 | Unclear | Low | Low | High | Low | Low | Low | Unclear | High |
| Shivhare et al. | 2019 | Low | Low | Low | Low | Low | Low | Low | Low | Low |
| Nema et al. | 2019 | Low | Low | Low | Low | Low | Low | Low | Low | Low |
| Peng et al. | 2019 | Low | Low | Low | Low | Low | Low | Low | Low | Low |
| Kalaiselvi et al. | 2019 | Low | Low | Low | Low | Low | Low | Low | Low | Low |
| Yang et al. | 2019 | Low | Low | Low | Low | Low | Low | Low | Low | Low |
| Virupakshappa and Amarapur | 2019 | Low | Low | Low | Low | Low | Low | Low | Low | Low |
| Wu et al. | 2019 | Low | Low | Low | Low | Low | Low | Low | Low | Low |
| Wang et al. | 2019 | Low | Low | Low | Low | Low | Low | Low | Low | Low |
| Rehman et al. | 2019 | Low | Low | Low | Low | Low | Low | Low | Low | Low |
| Kharrat and Neji | 2020 | Low | Low | Low | Low | Low | Low | Low | Low | Low |
| Rehman et al. | 2020 | Low | Low | Low | Low | Low | Low | Low | Low | Low |
| Khosravanian et al. | 2020 | Unclear | Low | Low | High | Low | Low | Low | Unclear | High |
| Srinivas and Rao | 2020 | Low | Low | Low | Low | Low | Low | Low | Low | Low |
| Yogananda et al. | 2020 | Low | Low | Low | Low | Low | Low | Low | Low | Low |
| Zhou et al. | 2020 | Low | Low | Low | Low | Low | Low | Low | Low | Low |
| Kumar et al. | 2020 | Unclear | Low | Low | Low | Low | Low | Low | Unclear | Low |
| Chen et al. | 2020 | Low | Low | Low | Low | Low | Low | Low | Low | Low |
| Chen et al. | 2020 | Low | Low | Low | Low | Low | Low | Low | Low | Low |
| Tjahyaningtijas et al. | 2020 | Low | Low | Low | Low | Low | Low | Low | Low | Low |
| Khan et al. | 2020 | Low | Low | Low | Low | Low | Low | Low | Low | Low |
| Zhang et al. | 2020 | Low | Low | Low | Low | Low | Low | Low | Low | Low |
| Zhang et al. | 2020 | Low | Low | Low | Low | Low | Low | Low | Low | Low |
| Xue et al. | 2020 | Low | Low | Low | Low | Low | Low | Low | Low | Low |
| Sun et al. | 2020 | Low | Low | Low | Low | Low | Low | Low | Low | Low |
| Liu et al. | 2020 | Low | Low | Low | Low | Low | Low | Low | Low | Low |
| Chaudhary et al. | 2020 | Low | Low | Low | Low | Low | Low | Low | Low | Low |
| Laukamp et al. | 2020 | Low | Low | Low | Low | Low | Low | Low | Low | Low |
| Thiruvenkadam and Perumal | 2016 | Low | Low | Low | High | Low | Low | Low | Low | High |
| Gupta et al. | 2020 | Unclear | Low | Low | High | High | Low | Low | High | High |
| Yepuganti et al. | 2020 | Unclear | Low | Low | High | High | Low | Low | High | High |
| Bousabarah et al. | 2020 | Low | Low | Low | Low | Unclear | Low | Low | Unclear | Low |
| Ejaz et al. | 2020 | Low | Low | Low | Low | Low | Low | Low | Low | Low |
| Pennig et al. | 2020 | Low | Low | Low | Low | Low | Low | Low | Low | Low |
| Mohamed et al. | 2020 | Unclear | Low | Low | High | Low | Low | Low | Unclear | High |
| Ali et al. | 2020 | Low | Low | Low | Low | Low | Low | Low | Low | Low |
| Katouli and Rahmani | 2020 | Low | Low | Low | High | Low | Low | Low | Low | High |
| Naceur et al. | 2020 | Low | Low | Low | Low | Low | Low | Low | Low | Low |
| Sharif et al. | 2020 | Low | Low | Low | Low | Low | Low | Low | Low | Low |
| Aboelenein et al. | 2020 | Low | Low | Low | Low | Low | Low | Low | Low | Low |
| Zaihani et al. | 2020 | Unclear | Low | Low | High | Low | Low | Low | Unclear | High |
| Hassen et al. | 2020 | Low | Low | Low | High | Low | Low | Low | Low | High |
| Chithra and Dheepa | 2020 | Low | Low | Low | High | Low | Low | Low | Low | High |
| Kao et al. | 2020 | Low | Low | Low | Low | Low | Low | Low | Low | Low |
| Pitchai et al. | 2020 | Low | Low | Low | Low | Low | Low | Low | Low | Low |
| Zeineldin et al. | 2020 | Low | Low | Low | Low | Low | Low | Low | Low | Low |
| Al-qazzaz et al. | 2020 | Low | Low | Low | Low | Low | Low | Low | Low | Low |
| Al-qazzaz et al. | 2020 | Low | Low | Low | Low | Low | Low | Low | Low | Low |
| Sathish and Elango | 2020 | Low | Low | Low | Low | Low | Low | Low | Low | Low |
| Banerjee and Mitra | 2020 | Low | Low | Low | Low | Low | Low | Low | Low | Low |
| Tripathi et al. | 2020 | Low | Low | Low | Low | Low | Low | Low | Low | Low |
| Kanniappan et al. | 2020 | Low | Low | Low | High | Unclear | Low | Low | Unclear | High |
| Debnath et al. | 2020 | Low | Low | Low | High | Low | Low | Low | Low | High |
| Baid et al. | 2020 | Low | Low | Low | Low | Low | Low | Low | Low | Low |
| Hu et al. | 2020 | Low | Low | Low | Low | Low | Low | Low | Low | Low |
| Zhou et al. | 2020 | Low | Low | Low | Low | Low | Low | Low | Low | Low |
| Zhou et al. | 2020 | Low | Low | Low | Low | Low | Low | Low | Low | Low |
| Barzegar and jamzad | 2020 | Low | Low | Low | Low | Low | Low | Low | Low | Low |
| Gyorfi et al. | 2021 | Low | Low | Low | Low | Low | Low | Low | Low | Low |
| Leva et al. | 2021 | Low | Low | Low | Low | Low | Low | Low | Low | Low |
| Biratu et al. | 2021 | Unclear | Low | Low | High | Low | Low | Low | Unclear | High |
| Rai et al. | 2021 | Low | Low | Low | Low | Low | Low | Low | Low | Low |
| Mitchell et al. | 2021 | Low | Low | Low | Low | Low | Low | Low | Low | Low |
| Abirami et al. | 2021 | Low | Low | Low | Low | Low | Low | Low | Low | Low |
| Al-Dabagh | 2021 | Low | Low | Low | High | Low | Low | Low | Low | High |
| Sohail et al. | 2021 | Low | Low | Low | Low | Low | Low | Low | Low | Low |
| Sran et al. | 2021 | Unclear | Low | Low | High | Low | Low | Low | Unclear | High |
| Saxena et al. | 2021 | Low | Low | Low | Low | Low | Low | Low | Low | Low |
| Takahashi et al. | 2021 | Low | Low | Low | Low | Low | Low | Low | Low | Low |
| Latif et al. | 2021 | Low | Low | Low | Low | Low | Low | Low | Low | Low |
| Zhang et al. | 2021 | Low | Low | Low | Low | Low | Low | Low | Low | Low |
| Lei et al. | 2021 | Low | Low | Low | High | Low | Low | Low | Low | High |
| Wang et al. | 2021 | Low | Low | Low | Low | Low | Low | Low | Low | Low |
| Zhao et al. | 2021 | Low | Low | Low | Low | Low | Low | Low | Low | Low |
| Cao et al. | 2021 | Low | Low | Low | Low | Low | Low | Low | Low | Low |
| Barzegar and jamzad | 2021 | Low | Low | Low | Low | Low | Low | Low | Low | Low |

# Supplementary table 10: Detailed QUADAS-2 Risk of Bias and Applicability for detection studies

| **Author** | **Year** | **Overall domain assessement bias - patient selection** | **Overall domain assessement applicability - patient selection** | **Overall domain assessement bias - index test** | **Overall domain assessement applicability - index test** | **Overall domain assessement bias - reference standard** | **Overall domain assessement applicability - reference standard** | **Overall domain assessement bias - flow and timing** | **Overall judgement of risk of bias** | **Overall judgement of applicability** |
| --- | --- | --- | --- | --- | --- | --- | --- | --- | --- | --- |
| Jayachandran and Dhanasekaran | 2012 | Low | Low | Low | Low | High | Low | Low | High | Low |
| Farjam et al. | 2012 | Low | Low | Low | Low | Low | Low | Low | Low | Low |
| Jayachandran and Dhanasekaran | 2013 | Low | Low | Low | Low | Low | Low | Low | Low | Low |
| Dvorák et al. | 2013 | Low | Low | Low | Low | High | Low | Low | High | Low |
| Dahshan et al. | 2014 | Low | Low | Low | Low | Low | Low | Low | Low | Low |
| Bahadure et al. | 2015 | Low | Low | Low | High | Low | Low | Low | Low | High |
| Helen and Kamaraj | 2015 | Unclear | Low | Low | High | High | Low | Low | High | High |
| Thirumurugan et al. | 2016 | Low | Low | Low | Low | Low | Low | Low | Low | Low |
| Banerjee et al. | 2016 | Low | Low | Low | High | Low | Low | Low | Low | High |
| Amin et al. | 2017 | Low | Low | Low | Low | Low | Low | Low | Low | Low |
| Gupta and Khanna | 2017 | Low | Low | Low | Low | Low | Low | Low | Low | Low |
| Anitha and Raja | 2017 | Unclear | Low | Low | Low | Low | Low | Low | Unclear | Low |
| Lahmiri | 2017 | Low | Low | Low | Low | Low | Low | Low | Low | Low |
| Deepa and Emmanuel | 2018 | Low | Low | Low | Low | Low | Low | Low | Low | Low |
| Selvapandian and Manivannan | 2018 | Unclear | Low | Low | Low | Low | Low | Low | Unclear | Low |
| Abd-Ellah et al. | 2018 | Low | Low | Low | Low | Low | Low | Low | Low | Low |
| Arunkumar et al. | 2018 | Unclear | Unclear | Low | Low | High | Low | Low | High | Unclear |
| Edalati-rad and Mosleh | 2019 | Low | Low | Low | Low | Low | Low | Low | Low | Low |
| Song et al. | 2019 | Low | Low | Low | Low | High | Low | Low | High | Low |
| Johnpeter and Ponnuchamy | 2019 | Low | Low | Low | High | Low | Low | Low | Low | High |
| Amin et al. | 2019 | Low | Low | Low | Low | Low | Low | Low | Low | Low |
| Alam et al. | 2019 | Unclear | Unclear | Low | High | High | Low | Low | High | High |
| Atici et al. | 2019 | Low | Low | Low | Low | Low | Low | Low | Low | Low |
| Sriramakrishnan et al. | 2019 | Low | Low | Low | Low | Low | Low | Low | Low | Low |
| Kebir et al. | 2019 | Low | Low | Low | Low | Low | Low | Low | Low | Low |
| Kalaiselvi et al. | 2019 | Low | Low | Low | Low | Low | Low | Low | Low | Low |
| Çinar and Yildirim | 2020 | Low | Low | Low | High | High | Low | Low | High | High |
| Devanathan and Venkatachalapathy | 2020 | Low | Low | Low | High | High | Low | Low | High | High |
| Dikici et al. | 2020 | Low | Low | Low | Low | Low | Low | Low | Low | Low |
| Dheepa and Chithra | 2020 | Unclear | Low | Low | High | Low | Low | Low | Unclear | High |
| Gurunathan and Krishnan | 2020 | Unclear | Low | Low | Low | Low | Low | Low | Unclear | Low |
| Wang et al. | 2020 | Low | Low | Low | Low | Low | Low | Low | Low | Low |
| Kesav and Rajini | 2020 | Low | Low | Low | Low | Low | Low | Low | Low | Low |
| Pennig et al. | 2020 | Low | Low | Low | Low | Low | Low | Low | Low | Low |
| Murali and Meena | 2020 | Unclear | Unclear | Low | High | High | Low | Low | High | High |
| Sathish and Elango | 2020 | Low | Low | Low | Low | Low | Low | Low | Low | Low |
| Kaur and Ghandi | 2020 | Low | Low | Low | Low | Low | Low | Low | Low | Low |
| Thangarajan and Chokkalingam | 2020 | Unclear | Low | Low | Low | High | Low | Low | High | Low |
| Kalaiselvi et al. | 2020 | Unclear | Low | Low | Low | Low | Low | Low | Unclear | Low |
| Rajinikanth et al. | 2020 | Low | Low | Low | Low | Low | Low | Low | Low | Low |
| Huang et al. | 2020 | Low | Low | Low | Low | Low | Low | Low | Low | Low |
| Chen et al. | 2021 | Low | Low | Low | Low | Low | Low | Low | Low | Low |
| Rai et al. | 2021 | Low | Low | Low | Low | Low | Low | Low | Low | Low |
| Patil and hamde | 2021 | Low | Low | Low | Low | Low | Low | Low | Low | Low |
| Simaiya et al. | 2021 | Unclear | Low | Low | Low | High | Low | Low | High | Low |
| Tejas P and Padma | 2021 | Unclear | Low | Low | High | High | Low | Low | High | High |

# Supplementary table 11: Segmentation meta-analysis for all studies for TML and DL methods

| **Segmentation type** | **Reference** | **Number of MRI slices** | **Mean DSC (95% CI)** | **Method** | **p** |
| --- | --- | --- | --- | --- | --- |
| **WT Segmentation** |  |  |  |  |  |
|  | Zhou et al. (2020)^52^ | 7130 | 0.9 [0.8981; 0.9019] | DL |  |
|  | Zhou et al. (2020)^52^ | 29605 | 0.91 [0.9092; 0.9108] | DL |  |
|  | Grøvik et al. (2019)^36^ | 8160 | 0.79 [0.7874; 0.7926] | DL |  |
|  | Wang et al. (2019)^38^ | 7130 | 0.9 [0.8984; 0.9016] | DL |  |
|  | Wang et al. (2019)^38^ | 22630 | 0.87 [0.8683; 0.8717] | DL |  |
|  | Wang et al. (2019)^38^ | 10230 | 0.91 [0.909; 0.911] | DL |  |
|  | Wang et al. (2019)^38^ | 29605 | 0.88 [0.8786; 0.8814] | DL |  |
|  | Li et al. (2019)^39^ | 42470 | 0.89 [0.8894; 0.8906] | DL |  |
|  | Li et al. (2019)^39^ | 44175 | 0.88 [0.8792; 0.8808] | DL |  |
|  | Li et al. (2019)^39^ | 17050 | 0.84 [0.8379; 0.8421] | DL |  |
|  | Khan et al. (2020)^53^ | 42470 | 0.81 [0.8095; 0.8105] | DL |  |
|  | Xue et al. (2020)^54^ | 144120 | 0.85 [0.8496; 0.8504] | DL |  |
|  | Xue et al. (2020)^54^ | 27720 | 0.84 [0.8392; 0.8408] | DL |  |
|  | Xue et al. (2020)^54^ | 26400 | 0.83 [0.8293; 0.8307] | DL |  |
|  | Mitchell et al. (2021)^63^ | 75045 | 0.87 [0.8699; 0.8701] | DL |  |
|  | Naceur et al. (2020)^56^ | 10230 | 0.87 [0.8675; 0.8725] | DL |  |
|  | Aboelenein et al. (2020)^57^ | 31000 | 0.85 [0.8488; 0.8512] | DL |  |
|  | Aboelenein et al. (2020)^57^ | 9300 | 0.86 [0.858; 0.862] | DL |  |
|  | Charron et al. (2018)^34^ | 412 | 0.79 [0.7697; 0.8103] | DL |  |
|  | Mlynarski et al. (2019)^44^ | 7130 | 0.9 [0.8981; 0.9019] | DL |  |
|  | Kao et al. (2020)^59^ | 10230 | 0.91 [0.9088; 0.9112] | DL |  |
|  | Mallick et al. (2019)^45^ | 349 | 0.93 [0.9143; 0.9457] | DL |  |
|  | Takahashi et al. (2021)^65^ | 58960 | 0.87 [0.8692; 0.8708] | DL |  |
|  | Takahashi et al. (2021)^65^ | 95744 | 0.78 [0.7791; 0.7809] | DL |  |
|  | Baid et al. (2020)^61^ | 10230 | 0.88 [0.8775; 0.8825] | DL |  |
|  | Baid et al. (2020)^61^ | 29605 | 0.85 [0.8481; 0.8519] | DL |  |
|  | Baid et al. (2020)^61^ | 44175 | 0.93 [0.929; 0.931] | DL |  |
|  | Liu et al. (2017)^29^ | 225 | 0.67 [0.6661; 0.6739] | DL |  |
|  | Total DL (I2 = 99.99%, p<0.001) |  | 0.86 [0.8381; 0.8806] |  |  |
|  | Essadike et al. (2018)^30^ | 1550 | 0.97 [0.9695; 0.9705] | TML |  |
|  | Essadike et al. (2018)^30^ | 1550 | 0.95 [0.949; 0.951] | TML |  |
|  | Pinto et al. (2018)^31^ | 3255 | 0.85 [0.8469; 0.8531] | TML |  |
|  | Pinto et al. (2018)^31^ | 620 | 0.78 [0.769; 0.791] | TML |  |
|  | Pinto et al. (2018)^31^ | 1550 | 0.85 [0.849; 0.851] | TML |  |
|  | Hasan et al. (2016)^20^ | 165 | 0.89 [0.8824; 0.8976] | TML |  |
|  | Hasan et al. (2016)^20^ | 3875 | 0.89 [0.8887; 0.8913] | TML |  |
|  | Sanjuàn et al. (2013)^15^ | 3168 | 0.72 [0.7165; 0.7235] | TML |  |
|  | Mbuyamba et al. (2016)^21^ | 312 | 0.91 [0.9033; 0.9167] | TML |  |
|  | Eltayeb et al. (2019)^37^ | 4320 | 0.9 [0.8985; 0.9015] | TML |  |
|  | Eltayeb et al. (2019)^37^ | 8000 | 0.93 [0.9289; 0.9311] | TML |  |
|  | Tong et al. (2019)^41^ | 7750 | 0.89 [0.8893; 0.8907] | TML |  |
|  | Dogra et al. (2019)^42^ | 3875 | 0.87 [0.8681; 0.8719] | TML |  |
|  | Dogra et al. (2019)^42^ | 3875 | 0.92 [0.9187; 0.9213] | TML |  |
|  | Thiruvenkadam and Perumal (2016)^22^ | 4650 | 0.8 [0.7966; 0.8034] | TML |  |
|  | Thiruvenkadam and Nagarajan (2020)^55^ | 3100 | 0.72 [0.7147; 0.7253] | TML |  |
|  | Thiruvenkadam and Nagarajan (2020)^55^ | 1550 | 0.75 [0.743; 0.757] | TML |  |
|  | Soltaninejad et al. (2017)^25^ | 4650 | 0.88 [0.8786; 0.8814] | TML |  |
|  | Soltaninejad et al. (2018)^33^ | 4650 | 0.89 [0.8889; 0.8911] | TML |  |
|  | Hassen et al. (2020)^58^ | 52080 | 0.94 [0.9397; 0.9403] | TML |  |
|  | Hassen et al. (2020)^58^ | 4650 | 0.9 [0.8989; 0.9011] | TML |  |
|  | Dvorák et al. (2015)^39^ | 357 | 0.85 [0.8386; 0.8614] | TML |  |
|  | Dvorák et al. (2015)^18^ | 443 | 0.82 [0.8032; 0.8368] | TML |  |
|  | Sriramakrishnan et al. (2019)^43^ | 4650 | 0.76 [0.7574; 0.7626] | TML |  |
|  | Sriramakrishnan et al. (2019)^43^ | 42470 | 0.81 [0.8095; 0.8105] | TML |  |
|  | Sran et al. (2021)^64^ | 1550 | 0.91 [0.909; 0.911] | TML |  |
|  | Li et al. (2018)^35^ | 42470 | 0.86 [0.8587; 0.8613] | TML |  |
|  | Kebir et al. (2019)^46^ | 3728 | 0.69 [0.6865; 0.6935] | TML |  |
|  | Alagarsamy et al. (2019)^47^ | 245 | 0.96 [0.9575; 0.9625] | TML |  |
|  | Shivhare et al. (2019)^48^ | 3410 | 0.92 [0.919; 0.921] | TML |  |
|  | Debnath et al. (2020)^60^ | 4650 | 0.95 [0.9494; 0.9506] | TML |  |
|  | Debnath et al. (2020)^60^ | 775 | 0.94 [0.9372; 0.9428] | TML |  |
|  | Kalaiselvi et al. (2019)^49^ | 4650 | 0.8 [0.798; 0.802] | TML |  |
|  | Imtiaz et al. (2017)^27^ | 3100 | 0.86 [0.8586; 0.8614] | TML |  |
|  | Kaur et al. (2017)^28^ | 4525 | 0.82 [0.8191; 0.8209] | TML |  |
|  | Kaur et al. (2017)^28^ | 1448 | 0.79 [0.7874; 0.7926] | TML |  |
|  | Wu et al. (2014)^16^ | 3100 | 0.61 [0.6037; 0.6163] | TML |  |
|  | Wu et al. (2014)^16^ | 1550 | 0.43 [0.4195; 0.4405] | TML |  |
|  | Liu et al. (2016)^23^ | 3255 | 0.89 [0.8873; 0.8927] | TML |  |
|  | Liu et al. (2016)^23^ | 2640 | 0.86 [0.8566; 0.8634] | TML |  |
|  | Wu et al. (2019)^50^ | 44175 | 0.85 [0.8493; 0.8507] | TML |  |
|  | Li et al. (2016)^24^ | 1550 | 0.85 [0.8495; 0.8505] | TML |  |
|  | Li et al. (2016)^24^ | 3255 | 0.73 [0.7273; 0.7327] | TML |  |
|  | Li et al. (2016)^24^ | 620 | 0.48 [0.4477; 0.5123] | TML |  |
|  | Rehman et al. (2019)^51^ | 3255 | 0.89 [0.8886; 0.8914] | TML |  |
|  | Total TML (I2 = 99.99%, p<0.001) |  | 0.83 [0.8001; 0.8683] |  |  |
|  | Total (I2 = 99.99%, p<0.001) |  | 0.84 [0.8216; 0.8662] |  | 0.21 |
| **TC Segmentation** |  |  |  |  |  |
|  | Zhou et al. (2020)^52^ | 7130 | 0.83 [0.8263; 0.8337] | DL |  |
|  | Zhou et al. (2020)^52^ | 29605 | 0.85 [0.8484; 0.8516] | DL |  |
|  | Wang et al. (2019)^38^ | 7130 | 0.84 [0.8363; 0.8437] | DL |  |
|  | Wang et al. (2019)^38^ | 22630 | 0.78 [0.7765; 0.7835] | DL |  |
|  | Wang et al. (2019)^38^ | 10230 | 0.87 [0.8675; 0.8725] | DL |  |
|  | Wang et al. (2019)^38^ | 29605 | 0.8 [0.7972; 0.8028] | DL |  |
|  | Li et al. (2019)^39^ | 42470 | 0.73 [0.7279; 0.7321] | DL |  |
|  | Li et al. (2019)^39^ | 44175 | 0.76 [0.7588; 0.7612] | DL |  |
|  | Li et al. (2019)^39^ | 17050 | 0.7 [0.6968; 0.7032] | DL |  |
|  | Khan et al. (2020)^53^ | 42470 | 0.76 [0.759; 0.761] | DL |  |
|  | Naceur et al. (2020)^56^ | 10230 | 0.75 [0.7453; 0.7547] | DL |  |
|  | Aboelenein et al. (2020)^57^ | 31000 | 0.81 [0.8072; 0.8128] | DL |  |
|  | Aboelenein et al. (2020)^57^ | 9300 | 0.81 [0.8055; 0.8145] | DL |  |
|  | Mlynarski et al. (2019)^44^ | 7130 | 0.81 [0.8056; 0.8144] | DL |  |
|  | Kao et al. (2020)^59^ | 10230 | 0.82 [0.8159; 0.8241] | DL |  |
|  | Baid et al. (2020)^61^ | 10230 | 0.83 [0.8265; 0.8335] | DL |  |
|  | Baid et al. (2020)^61^ | 29605 | 0.77 [0.7668; 0.7732] | DL |  |
|  | Baid et al. (2020)^61^ | 44175 | 0.92 [0.9188; 0.9212] | DL |  |
|  | Liu et al. (2017)^29^ | 265 | 0.75 [0.7416; 0.7584] | DL |  |
|  | Total DL (I2 = 99.97%, p<0.001) |  | 0.8 [0.774; 0.825] |  |  |
|  | Pinto et al. (2018)^31^ | 3255 | 0.71 [0.7007; 0.7193] | TML |  |
|  | Pinto et al. (2018)^31^ | 620 | 0.51 [0.4825; 0.5375] | TML |  |
|  | Pinto et al. (2018)^31^ | 1550 | 0.78 [0.775; 0.785] | TML |  |
|  | Thiruvenkadam and Perumal (2016)^22^ | 4650 | 0.59 [0.5837; 0.5963] | TML |  |
|  | Thiruvenkadam and Nagarajan (2020)^55^ | 3100 | 0.5 [0.4933; 0.5067] | TML |  |
|  | Thiruvenkadam and Nagarajan (2020)^55^ | 1550 | 0.6 [0.5881; 0.6119] | TML |  |
|  | Soltaninejad et al. (2018)^33^ | 4650 | 0.8 [0.7974; 0.8026] | TML |  |
|  | Hassen et al. (2020)^58^ | 52080 | 0.85 [0.8497; 0.8503] | TML |  |
|  | Hassen et al. (2020)^58^ | 4650 | 0.85 [0.8483; 0.8517] | TML |  |
|  | Sriramakrishnan et al. (2019)^43^ | 4650 | 0.53 [0.5245; 0.5355] | TML |  |
|  | Sriramakrishnan et al. (2019)^43^ | 42470 | 0.49 [0.4883; 0.4917] | TML |  |
|  | Sriramakrishnan et al. (2019)^43^ | 42470 | 0.47 [0.4681; 0.4719] | TML |  |
|  | Shivhare et al. (2019)^48^ | 3410 | 0.81 [0.8046; 0.8154] | TML |  |
|  | Wu et al. (2014)^16^ | 3100 | 0.62 [0.6158; 0.6242] | TML |  |
|  | Wu et al. (2014)^16^ | 1550 | 0.59 [0.582; 0.598] | TML |  |
|  | Li et al. (2016)^24^ | 1550 | 0.75 [0.746; 0.754] | TML |  |
|  | Li et al. (2016)^24^ | 3255 | 0.56 [0.5528; 0.5672] | TML |  |
|  | Li et al. (2016)^24^ | 620 | 0.38 [0.3438; 0.4162] | TML |  |
|  | Total TML (I2 = 100%, p<0.001) |  | 0.63 [0.5608; 0.7053] |  |  |
|  | Total (I2 = 99.99%, p<0.001) |  | 0.72 [0.6733; 0.7639] |  | <0.0001 |
| **ET Segmentation** |  |  |  |  |  |
|  | Zhou et al. (2020)^52^ | 7130 | 0.77 [0.7637; 0.7763] | DL |  |
|  | Zhou et al. (2020)^52^ | 29605 | 0.8 [0.7974; 0.8026] | DL |  |
|  | Wang et al. (2019)^38^ | 7130 | 0.79 [0.7847; 0.7953] | DL |  |
|  | Wang et al. (2019)^38^ | 22630 | 0.78 [0.7771; 0.7829] | DL |  |
|  | Wang et al. (2019)^38^ | 10230 | 0.81 [0.8057; 0.8143] | DL |  |
|  | Wang et al. (2019)^38^ | 29605 | 0.75 [0.747; 0.753] | DL |  |
|  | Li et al. (2019)^39^ | 42470 | 0.73 [0.7271; 0.7329] | DL |  |
|  | Li et al. (2019)^39^ | 44175 | 0.64 [0.6374; 0.6426] | DL |  |
|  | Li et al. (2019)^39^ | 17050 | 0.6 [0.5952; 0.6048] | DL |  |
|  | Khan et al. (2020)^53^ | 42470 | 0.73 [0.7291; 0.7309] | DL |  |
|  | Naceur et al. (2020)^56^ | 10230 | 0.75 [0.745; 0.755] | DL |  |
|  | Aboelenein et al. (2020)^57^ | 31000 | 0.74 [0.7369; 0.7431] | DL |  |
|  | Aboelenein et al. (2020)^57^ | 9300 | 0.74 [0.7357; 0.7443] | DL |  |
|  | Mlynarski et al. (2019)^44^ | 7130 | 0.77 [0.7644; 0.7756] | DL |  |
|  | Kao et al. (2020)^59^ | 10230 | 0.78 [0.775; 0.785] | DL |  |
|  | Baid et al. (2020)^61^ | 10230 | 0.75 [0.7448; 0.7552] | DL |  |
|  | Baid et al. (2020)^61^ | 29605 | 0.67 [0.6665; 0.6735] | DL |  |
|  | Baid et al. (2020)^61^ | 44175 | 0.82 [0.8175; 0.8225] | DL |  |
|  | Liu et al. (2017)^29^ | 265 | 0.81 [0.8052; 0.8148] | DL |  |
|  | Total DL (I2 = 99.91%, p<0.001) |  | 0.75 [0.7209; 0.7769] |  |  |
|  | Pinto et al. (2018)^31^ | 3255 | 0.71 [0.7021; 0.7179] | TML |  |
|  | Pinto et al. (2018)^31^ | 1550 | 0.75 [0.746; 0.754] | TML |  |
|  | Thiruvenkadam and Perumal (2016)^22^ | 3100 | 0.6 [0.5926; 0.6074] | TML |  |
|  | Thiruvenkadam and Nagarajan (2020)^55^ | 3100 | 0.5 [0.4908; 0.5092] | TML |  |
|  | Hassen et al. (2020)^58^ | 52080 | 0.76 [0.7593; 0.7607] | TML |  |
|  | Sriramakrishnan et al. (2019)^43^ | 3100 | 0.58 [0.5705; 0.5895] | TML |  |
|  | Shivhare et al. (2019)^48^ | 3410 | 0.83 [0.8256; 0.8344] | TML |  |
|  | Steed et al. (2015)^19^ | 1933 | 0.92 [0.9187; 0.9213] | TML |  |
|  | Li et al. (2016)^24^ | 1550 | 0.69 [0.6895; 0.6905] | TML |  |
|  | Li et al. (2016)^24^ | 3255 | 0.54 [0.5386; 0.5414] | TML |  |
|  | Total TML (I2 = 100%, p<0.001) |  | 0.69 [0.5926; 0.7835] |  |  |
|  | Total (I2 = 99.99%, p<0.001) |  | 0.73 [0.6924; 0.7636] |  | 0.17 |
| WT=Whole tumour; TC=Tumour core; ET=Enhancing tumour; DSC=Dice Score Coefficient; DL=Deep learning; TML=Traditional machine learning | | | | | |

# Supplementary table 12: Segmentation meta-analysis for out-of-sample-externally validated studies for TML and DL methods

| **Segmentation type** | **Reference** | **Number of images** | **Mean DSC (95% CI)** | **Method** | **p** |
| --- | --- | --- | --- | --- | --- |
| **WT segmentation** |  |  |  |  |  |
|  | Zhou et al. (2020) | 7130 | 0.9 [0.8981; 0.9019] | DL |  |
|  | Zhou et al. (2020) | 29605 | 0.91 [0.9092; 0.9108] | DL |  |
|  | Grøvik et al. (2019) | 8160 | 0.79 [0.7874; 0.7926] | DL |  |
|  | Wang et al. (2019) | 7130 | 0.9 [0.8984; 0.9016] | DL |  |
|  | Wang et al. (2019) | 22630 | 0.87 [0.8683; 0.8717] | DL |  |
|  | Wang et al. (2019) | 10230 | 0.91 [0.909; 0.911] | DL |  |
|  | Wang et al. (2019) | 29605 | 0.88 [0.8786; 0.8814] | DL |  |
|  | Li et al. (2019) | 42470 | 0.89 [0.8894; 0.8906] | DL |  |
|  | Li et al. (2019) | 44175 | 0.88 [0.8792; 0.8808] | DL |  |
|  | Li et al. (2019) | 17050 | 0.84 [0.8379; 0.8421] | DL |  |
|  | Xue et al. (2020) | 144120 | 0.85 [0.8496; 0.8504] | DL |  |
|  | Xue et al. (2020) | 27720 | 0.84 [0.8392; 0.8408] | DL |  |
|  | Xue et al. (2020) | 26400 | 0.83 [0.8293; 0.8307] | DL |  |
|  | Naceur et al. (2020) | 10230 | 0.87 [0.8675; 0.8725] | DL |  |
|  | Aboelenein et al. (2020) | 9300 | 0.86 [0.858; 0.862] | DL |  |
|  | Charron et al. (2018) | 412 | 0.79 [0.7697; 0.8103] | DL |  |
|  | Mlynarski et al. (2019) | 7130 | 0.9 [0.8981; 0.9019] | DL |  |
|  | Kao et al. (2020) | 10230 | 0.91 [0.9088; 0.9112] | DL |  |
|  | Takahashi et al. (2021) | 58960 | 0.87 [0.8692; 0.8708] | DL |  |
|  | Takahashi et al. (2021) | 95744 | 0.78 [0.7791; 0.7809] | DL |  |
|  | Baid et al. (2020) | 10230 | 0.88 [0.8775; 0.8825] | DL |  |
|  | Baid et al. (2020) | 29605 | 0.85 [0.8481; 0.8519] | DL |  |
|  | Baid et al. (2020) | 44175 | 0.93 [0.929; 0.931] | DL |  |
|  | Total DL (I2 = 99.98%, p<0.001) |  | 0.87 [0.8491; 0.8843] |  |  |
|  | Pinto et al. (2018) | 3255 | 0.85 [0.8469; 0.8531] | TML |  |
|  | Pinto et al. (2018) | 620 | 0.78 [0.769; 0.791] | TML |  |
|  | Pinto et al. (2018) | 1550 | 0.85 [0.849; 0.851] | TML |  |
|  | Ali M. Hasan (2016) | 3875 | 0.89 [0.8887; 0.8913] | TML |  |
|  | Soltaninejad et al. (2017) | 4650 | 0.88 [0.8786; 0.8814] | TML |  |
|  | Soltaninejad et al. (2018) | 4650 | 0.89 [0.8889; 0.8911] | TML |  |
|  | Dvorák et al. (2015) | 357 | 0.85 [0.8386; 0.8614] | TML |  |
|  | Dvorák et al. (2015) | 443 | 0.82 [0.8032; 0.8368] | TML |  |
|  | Li et al. (2016) | 1550 | 0.85 [0.8495; 0.8505] | TML |  |
|  | Li et al. (2016) | 3255 | 0.73 [0.7273; 0.7327] | TML |  |
|  | Li et al. (2016) | 620 | 0.48 [0.4477; 0.5123] | TML |  |
|  | Total TML (I2 = 99.94%, p<0.001) |  | 0.81 [0.7279; 0.886] |  |  |
|  | Total (I2 = 99.97%, p<0.001) |  | 0.85 [0.8205; 0.8746] |  | 0.1 |
| **TC segmentation** |  |  |  |  |  |
|  | Zhou et al. (2020) | 7130 | 0.83 [0.8263; 0.8337] | DL |  |
|  | Zhou et al. (2020) | 29605 | 0.85 [0.8484; 0.8516] | DL |  |
|  | Wang et al. (2019) | 7130 | 0.84 [0.8363; 0.8437] | DL |  |
|  | Wang et al. (2019) | 22630 | 0.78 [0.7765; 0.7835] | DL |  |
|  | Wang et al. (2019) | 10230 | 0.87 [0.8675; 0.8725] | DL |  |
|  | Wang et al. (2019) | 29605 | 0.8 [0.7972; 0.8028] | DL |  |
|  | Li et al. (2019) | 42470 | 0.73 [0.7279; 0.7321] | DL |  |
|  | Li et al. (2019) | 44175 | 0.76 [0.7588; 0.7612] | DL |  |
|  | Li et al. (2019) | 17050 | 0.7 [0.6968; 0.7032] | DL |  |
|  | Naceur et al. (2020) | 10230 | 0.75 [0.7453; 0.7547] | DL |  |
|  | Aboelenein et al. (2020) | 9300 | 0.81 [0.8055; 0.8145] | DL |  |
|  | Mlynarski et al. (2019) | 7130 | 0.81 [0.8056; 0.8144] | DL |  |
|  | Kao et al. (2020) | 10230 | 0.82 [0.8159; 0.8241] | DL |  |
|  | Baid et al. (2020) | 10230 | 0.83 [0.8265; 0.8335] | DL |  |
|  | Baid et al. (2020) | 29605 | 0.77 [0.7668; 0.7732] | DL |  |
|  | Baid et al. (2020) | 44175 | 0.92 [0.9188; 0.9212] | DL |  |
|  | Total DL (I2 = 99.97%, p<0.001) |  | 0.8 [0.7749; 0.8338] |  |  |
|  | Pinto et al. (2018) | 3255 | 0.71 [0.7007; 0.7193] | TML |  |
|  | Pinto et al. (2018) | 620 | 0.51 [0.4825; 0.5375] | TML |  |
|  | Pinto et al. (2018) | 1550 | 0.78 [0.775; 0.785] | TML |  |
|  | Soltaninejad et al. (2018) | 4650 | 0.8 [0.7974; 0.8026] | TML |  |
|  | Li et al. (2016) | 1550 | 0.75 [0.746; 0.754] | TML |  |
|  | Li et al. (2016) | 3255 | 0.56 [0.5528; 0.5672] | TML |  |
|  | Li et al. (2016) | 620 | 0.38 [0.3438; 0.4162] | TML |  |
|  | Total TML (I2 = 99.87%, p<0.001) |  | 0.64 [0.4946; 0.7895] |  |  |
|  | Total (I2 = 99.96%, p<0.001) |  | 0.76 [0.7028; 0.8078] |  | 0.0087 |
| WT=Whole tumour; TC=Tumour core; ET=Enhancing tumour; DSC=Dice Score Coefficient; DL=Deep learning; TML=Traditional machine learning | | | | | |

# Supplementary table 13: Subgroup segmentation meta-analysis by tumour type

| **Segmentation type** | **Reference** | **Number of MRI slices** | **Mean DSC (95% CI)** | **Tumour** | **p** |
| --- | --- | --- | --- | --- | --- |
| **WT segmentation** |  |  |  |  |  |
|  | Pinto et al. (2018) | 3255 | 0.85 [0.8469; 0.8531] | HGG |  |
|  | Pinto et al. (2018) | 1550 | 0.85 [0.849; 0.851] | HGG |  |
|  | Sanjuàn et al. (2013) | 1408 | 0.7 [0.6932; 0.7068] | HGG |  |
|  | Eltayeb et al. (2019) | 3040 | 0.9 [0.8979; 0.9021] | HGG |  |
|  | Eltayeb et al. (2019) | 4000 | 0.92 [0.9181; 0.9219] | HGG |  |
|  | Khan et al. (2020) | 34100 | 0.83 [0.8296; 0.8304] | HGG |  |
|  | Jyotsna Dogra (2019) | 3875 | 0.87 [0.8681; 0.8719] | HGG |  |
|  | Thiruvenkadam and Perumal (2016) | 3100 | 0.8 [0.7961; 0.8039] | HGG |  |
|  | Thiruvenkadam and Nagarajan (2020) | 3100 | 0.72 [0.7147; 0.7253] | HGG |  |
|  | Soltaninejad et al. (2017) | 3100 | 0.88 [0.8782; 0.8818] | HGG |  |
|  | Dvorák et al. (2015) | 357 | 0.86 [0.8507; 0.8693] | HGG |  |
|  | Dvorák et al. (2015) | 443 | 0.86 [0.8488; 0.8712] | HGG |  |
|  | Sriramakrishnan et al. (2019) | 3100 | 0.76 [0.7568; 0.7632] | HGG |  |
|  | Sriramakrishnan et al. (2019) | 34100 | 0.82 [0.8195; 0.8205] | HGG |  |
|  | Mallick et al. (2019) | 349 | 0.93 [0.9143; 0.9457] | HGG |  |
|  | Li et al. (2018) | 34100 | 0.86 [0.8585; 0.8615] | HGG |  |
|  | Kebir et al. (2019) | 3728 | 0.69 [0.6865; 0.6935] | HGG |  |
|  | Debnath et al. (2020) | 4650 | 0.95 [0.9494; 0.9506] | HGG |  |
|  | Kalaiselvi et al. (2019) | 3100 | 0.8 [0.7975; 0.8025] | HGG |  |
|  | Imtiaz et al. (2017) | 3100 | 0.86 [0.8586; 0.8614] | HGG |  |
|  | Wu et al. (2014) | 3100 | 0.61 [0.6037; 0.6163] | HGG |  |
|  | Wu et al. (2019) | 32550 | 0.85 [0.8491; 0.8509] | HGG |  |
|  | Li et al. (2016) | 1550 | 0.85 [0.8495; 0.8505] | HGG |  |
|  | Li et al. (2016) | 3255 | 0.73 [0.7273; 0.7327] | HGG |  |
|  | Rehman et al. (2019) | 2480 | 0.88 [0.878; 0.882] | HGG |  |
|  | Total HGG (I2 = 99.99%, p<0.001) |  | 0.83 [0.7912; 0.8591] |  |  |
|  | Pinto et al. (2018) | 620 | 0.78 [0.769; 0.791] | LGG |  |
|  | Sanjuàn et al. (2013) | 1584 | 0.72 [0.7166; 0.7234] | LGG |  |
|  | Eltayeb et al. (2019) | 1280 | 0.9 [0.8973; 0.9027] | LGG |  |
|  | Eltayeb et al. (2019) | 4000 | 0.94 [0.9391; 0.9409] | LGG |  |
|  | Khan et al. (2020) | 8370 | 0.79 [0.7885; 0.7915] | LGG |  |
|  | Jyotsna Dogra (2019) | 3875 | 0.92 [0.9187; 0.9213] | LGG |  |
|  | Thiruvenkadam and Perumal (2016) | 1550 | 0.79 [0.7835; 0.7965] | LGG |  |
|  | Thiruvenkadam and Nagarajan (2020) | 1550 | 0.75 [0.743; 0.757] | LGG |  |
|  | Soltaninejad et al. (2017) | 1550 | 0.89 [0.888; 0.892] | LGG |  |
|  | Dvorák et al. (2015) | 357 | 0.85 [0.8376; 0.8624] | LGG |  |
|  | Dvorák et al. (2015) | 443 | 0.79 [0.7695; 0.8105] | LGG |  |
|  | Sriramakrishnan et al. (2019) | 1550 | 0.75 [0.745; 0.755] | LGG |  |
|  | Sriramakrishnan et al. (2019) | 8370 | 0.79 [0.7889; 0.7911] | LGG |  |
|  | Li et al. (2018) | 8370 | 0.87 [0.8674; 0.8726] | LGG |  |
|  | Debnath et al. (2020) | 775 | 0.94 [0.9372; 0.9428] | LGG |  |
|  | Kalaiselvi et al. (2019) | 1550 | 0.81 [0.807; 0.813] | LGG |  |
|  | Kaur et al. (2017) | 4525 | 0.82 [0.8191; 0.8209] | LGG |  |
|  | Kaur et al. (2017) | 1448 | 0.79 [0.7874; 0.7926] | LGG |  |
|  | Wu et al. (2014) | 1550 | 0.43 [0.4195; 0.4405] | LGG |  |
|  | Wu et al. (2019) | 11625 | 0.86 [0.8587; 0.8613] | LGG |  |
|  | Li et al. (2016) | 620 | 0.48 [0.4477; 0.5123] | LGG |  |
|  | Rehman et al. (2019) | 775 | 0.92 [0.9179; 0.9221] | LGG |  |
|  | Total LGG (I2 = 99.98%, p<0.001) |  | 0.8 [0.7423; 0.8564] |  |  |
|  | Grøvik et al. (2019) | 8160 | 0.79 [0.7874; 0.7926] | MET |  |
|  | Xue et al. (2020) | 144120 | 0.85 [0.8496; 0.8504] | MET |  |
|  | Xue et al. (2020) | 27720 | 0.84 [0.8392; 0.8408] | MET |  |
|  | Xue et al. (2020) | 26400 | 0.83 [0.8293; 0.8307] | MET |  |
|  | Charron et al. (2018) | 412 | 0.79 [0.7697; 0.8103] | MET |  |
|  | Liu et al. (2017) | 225 | 0.67 [0.6661; 0.6739] | MET |  |
|  | Liu et al. (2016) | 2640 | 0.86 [0.8566; 0.8634] | MET |  |
|  | Total MET (I2 = 99.95%, p<0.001) |  | 0.8 [0.7439; 0.8648] |  |  |
|  | Total (I2 = 99.98%, p<0.001) |  | 0.81 [0.7844; 0.8397] |  | 0.64 |
| **TC segmentation** |  |  |  |  |  |
|  | Pinto et al. (2018) | 3255 | 0.71 [0.7007; 0.7193] | HGG |  |
|  | Pinto et al. (2018) | 1550 | 0.78 [0.775; 0.785] | HGG |  |
|  | Khan et al. (2020) | 34100 | 0.78 [0.7789; 0.7811] | HGG |  |
|  | Thiruvenkadam and Nagarajan (2020) | 3100 | 0.71 [0.7051; 0.7149] | HGG |  |
|  | Thiruvenkadam and Perumal (2016) | 3100 | 0.5 [0.4933; 0.5067] | HGG |  |
|  | Sriramakrishnan et al. (2019) | 3100 | 0.65 [0.6426; 0.6574] | HGG |  |
|  | Sriramakrishnan et al. (2019) | 34100 | 0.61 [0.6082; 0.6118] | HGG |  |
|  | Wu et al. (2014) | 3100 | 0.62 [0.6158; 0.6242] | HGG |  |
|  | Li et al. (2016) | 1550 | 0.75 [0.746; 0.754] | HGG |  |
|  | Li et al. (2016) | 3255 | 0.56 [0.5528; 0.5672] | HGG |  |
|  | Total HGG (I2 = 99.97%, p<0.001) |  | 0.67 [0.5991; 0.735] |  |  |
|  | Pinto et al. (2018) | 620 | 0.51 [0.4825; 0.5375] | LGG |  |
|  | Khan et al. (2020) | 8370 | 0.74 [0.7374; 0.7426] | LGG |  |
|  | Thiruvenkadam and Nagarajan (2020) | 1550 | 0.35 [0.3415; 0.3585] | LGG |  |
|  | Thiruvenkadam and Perumal (2016) | 1550 | 0.6 [0.5881; 0.6119] | LGG |  |
|  | Sriramakrishnan et al. (2019) | 1550 | 0.4 [0.3915; 0.4085] | LGG |  |
|  | Sriramakrishnan et al. (2019) | 8370 | 0.36 [0.3559; 0.3641] | LGG |  |
|  | Wu et al. (2014) | 1550 | 0.59 [0.582; 0.598] | LGG |  |
|  | Li et al. (2016) | 620 | 0.38 [0.3438; 0.4162] | LGG |  |
|  | Total LGG (I2 = 99.98%, p<0.001) |  | 0.49 [0.3724; 0.6105] |  |  |
|  | Total (I2 = 99.98%, p<0.001) |  | 0.59 [0.5168; 0.6615] |  | 0.0027 |
| **ET segmentation** |  |  |  |  |  |
|  | Pinto et al. (2018) | 3255 | 0.71 [0.7021; 0.7179] | HGG |  |
|  | Pinto et al. (2018) | 1550 | 0.75 [0.746; 0.754] | HGG |  |
|  | Khan et al. (2020) | 34100 | 0.74 [0.7392; 0.7408] | HGG |  |
|  | Thiruvenkadam and Perumal (2016) | 3100 | 0.6 [0.5926; 0.6074] | HGG |  |
|  | Thiruvenkadam and Nagarajan (2020) | 3100 | 0.5 [0.4908; 0.5092] | HGG |  |
|  | Padmanaban Sriramakrishnan (2019) | 3100 | 0.58 [0.5705; 0.5895] | HGG |  |
|  | Padmanaban Sriramakrishnan (2019) | 34100 | 0.65 [0.6479; 0.6521] | HGG |  |
|  | Steed et al. (2015) | 1933 | 0.92 [0.9187; 0.9213] | HGG |  |
|  | Liu et al. (2016) | 1550 | 0.69 [0.6895; 0.6905] | HGG |  |
|  | Liu et al. (2016) | 3255 | 0.54 [0.5386; 0.5414] | HGG |  |
|  | Total (I2 = 99.99%, p<0.001) |  | 0.67 [0.5804; 0.7556] |  |  |
| WT=Whole tumour; TC=Tumour core; ET=Enhancing tumour; DSC=Dice Score Coefficient; LGG=Low Grade Glioma; HGG=High Grade Glioma; MET=Metastatic brain tumour | | | | | |

# Supplementary table 14: Automated vs human operator segmentation meta-analysis

| **Segmentation type** | **Reference** | | **Ground Truth** | **Number of MRI slices** | **Mean DSC (95% CI)** | **Method** | **p** |
| --- | --- | --- | --- | --- | --- | --- | --- |
| WT segmentation |  | |  |  |  |  |  |
|  | Sanjuàn et al. (2013) | | Two independent experienced observers | 3168 | 0.72 [0.7165; 0.7235] | Automated |  |
|  | Eltayeb et al. (2019) | | Four independent experienced observers from the BRATS challenge | 4320 | 0.9 [0.8985; 0.9015] | Automated |  |
|  | Thiruvenkadam and Perumal (2016) | | Four independent experienced observers from the BRATS challenge | 4650 | 0.8 [0.7966; 0.8034] | Automated |  |
|  | Thiruvenkadam and Nagarajan (2020) | | Four independent experienced observers from the BRATS challenge | 3100 | 0.72 [0.7147; 0.7253] | Automated |  |
|  | Thiruvenkadam and Nagarajan (2020) | | Four independent experienced observers from the BRATS challenge | 1550 | 0.75 [0.743; 0.757] | Automated |  |
|  | Soltaninejad et al. (2017) | | Four independent experienced observers from the BRATS challenge | 4650 | 0.88 [0.8786; 0.8814] | Automated |  |
|  | Soltaninejad et al. (2018) | | Four independent experienced observers from the BRATS challenge | 4650 | 0.89 [0.8889; 0.8911] | Automated |  |
|  | Hassen et al. (2020) | | Four independent experienced observers from the BRATS challenge | 4650 | 0.9 [0.8989; 0.9011] | Automated |  |
|  | Sriramakrishnan et al. (2019) | | Four independent experienced observers from the BRATS challenge | 4650 | 0.76 [0.7574; 0.7626] | Automated |  |
|  | Debnath et al. (2020) | | Four independent experienced observers from the BRATS challenge | 4650 | 0.95 [0.9494; 0.9506] | Automated |  |
|  | Debnath et al. (2020) | | Four independent experienced observers from the BRATS challenge | 775 | 0.94 [0.9372; 0.9428] | Automated |  |
|  | Kalaiselvi et al. (2019) | | Four independent experienced observers from the BRATS challenge | 4650 | 0.8 [0.798; 0.802] | Automated |  |
|  | Imtiaz et al. (2017) | | Four independent experienced observers from the BRATS challenge | 3100 | 0.86 [0.8586; 0.8614] | Automated |  |
|  | Wu et al. (2014) | | Four independent experienced observers from the BRATS challenge | 3100 | 0.61 [0.6037; 0.6163] | Automated |  |
|  | Wu et al. (2014) | | Four independent experienced observers from the BRATS challenge | 1550 | 0.43 [0.4195; 0.4405] | Automated |  |
|  | Rehman et al. (2019) | | Four independent experienced observers from the BRATS challenge | 3255 | 0.89 [0.8886; 0.8914] | Automated |  |
|  | Total Automated (I2 = 99.98%, p<0.001) | |  |  | 0.8 [0.7275; 0.8726] |  |  |
|  | Sanjuàn et al. (2013) | | Two independent experienced observers | 3168 | 0.84 [0.8362; 0.8438] | Human |  |
|  | Eltayeb et al. (2019) | | Four independent experienced observers from the BRATS challenge | 4320 | 0.85 [0.8476; 0.8524] | Human |  |
|  | Thiruvenkadam and Perumal (2016) | | Four independent experienced observers from the BRATS challenge | 4650 | 0.85 [0.8477; 0.8523] | Human |  |
|  | Thiruvenkadam and Nagarajan (2020) | | Four independent experienced observers from the BRATS challenge | 3100 | 0.88 [0.8793; 0.8807] | Human |  |
|  | Thiruvenkadam and Nagarajan (2020) | | Four independent experienced observers from the BRATS challenge | 1550 | 0.84 [0.839; 0.841] | Human |  |
|  | Soltaninejad et al. (2017) | | Four independent experienced observers from the BRATS challenge | 4650 | 0.85 [0.8477; 0.8523] | Human |  |
|  | Soltaninejad et al. (2018) | | Four independent experienced observers from the BRATS challenge | 4650 | 0.85 [0.8477; 0.8523] | Human |  |
|  | Hassen et al. (2020) | | Four independent experienced observers from the BRATS challenge | 4650 | 0.85 [0.8477; 0.8523] | Human |  |
|  | Sriramakrishnan et al. (2019) | | Four independent experienced observers from the BRATS challenge | 4650 | 0.85 [0.8477; 0.8523] | Human |  |
|  | Debnath et al. (2020) | | Four independent experienced observers from the BRATS challenge | 4650 | 0.88 [0.8794; 0.8806] | Human |  |
|  | Debnath et al. (2020) | | Four independent experienced observers from the BRATS challenge | 775 | 0.84 [0.8386; 0.8414] | Human |  |
|  | Kalaiselvi et al. (2019) | | Four independent experienced observers from the BRATS challenge | 4650 | 0.85 [0.8477; 0.8523] | Human |  |
|  | Imtiaz et al. (2017) | | Four independent experienced observers from the BRATS challenge | 3100 | 0.88 [0.8793; 0.8807] | Human |  |
|  | Wu et al. (2014) | | Four independent experienced observers from the BRATS challenge | 3100 | 0.88 [0.8793; 0.8807] | Human |  |
|  | Wu et al. (2014) | | Four independent experienced observers from the BRATS challenge | 1550 | 0.84 [0.839; 0.841] | Human |  |
|  | Rehman et al. (2019) | | Four independent experienced observers from the BRATS challenge | 3255 | 0.85 [0.8473; 0.8527] | Human |  |
|  | Total Human (I2 = 99.9%, p<0.001) | |  |  | 0.86 [0.8468; 0.8633] |  |  |
|  | Total (I2 = 99.98%, p<0.001) | |  |  | 0.83 [0.7918; 0.8633] |  | 0.11 |
| TC segmentation |  | |  |  |  |  |  |
|  | Thiruvenkadam and Perumal (2016) | | Four independent experienced observers from the BRATS challenge | 4650 | 0.59 [0.5837; 0.5963] | Automated |  |
|  | Thiruvenkadam and Nagarajan (2020) | | Four independent experienced observers from the BRATS challenge | 3100 | 0.5 [0.4933; 0.5067] | Automated |  |
|  | Thiruvenkadam and Nagarajan (2020) | | Four independent experienced observers from the BRATS challenge | 1550 | 0.6 [0.5881; 0.6119] | Automated |  |
|  | Soltaninejad et al. (2018) | | Four independent experienced observers from the BRATS challenge | 4650 | 0.8 [0.7974; 0.8026] | Automated |  |
|  | Hassen et al. (2020) | | Four independent experienced observers from the BRATS challenge | 4650 | 0.85 [0.8483; 0.8517] | Automated |  |
|  | Sriramakrishnan et al. (2019) | | Four independent experienced observers from the BRATS challenge | 4650 | 0.53 [0.5245; 0.5355] | Automated |  |
|  | Wu et al. (2014) | | Four independent experienced observers from the BRATS challenge | 3100 | 0.62 [0.6158; 0.6242] | Automated |  |
|  | Wu et al. (2014) | | Four independent experienced observers from the BRATS challenge | 1550 | 0.59 [0.582; 0.598] | Automated |  |
|  | Total Automated (I2 = 99.98%, p<0.001) | |  |  | 0.64 [0.5311; 0.739] |  |  |
|  | Thiruvenkadam and Perumal (2016) | | Four independent experienced observers from the BRATS challenge | 4650 | 0.75 [0.7431; 0.7569] | Human |  |
|  | Thiruvenkadam and Nagarajan (2020) | | Four independent experienced observers from the BRATS challenge | 3100 | 0.93 [0.9289; 0.9311] | Human |  |
|  | Thiruvenkadam and Nagarajan (2020) | | Four independent experienced observers from the BRATS challenge | 1550 | 0.67 [0.6561; 0.6839] | Human |  |
|  | Soltaninejad et al. (2018) | | Four independent experienced observers from the BRATS challenge | 4650 | 0.75 [0.7431; 0.7569] | Human |  |
|  | Hassen et al. (2020) | | Four independent experienced observers from the BRATS challenge | 4650 | 0.75 [0.7431; 0.7569] | Human |  |
|  | Sriramakrishnan et al. (2019) | | Four independent experienced observers from the BRATS challenge | 4650 | 0.75 [0.7431; 0.7569] | Human |  |
|  | Wu et al. (2014) | | Four independent experienced observers from the BRATS challenge | 3100 | 0.93 [0.9289; 0.9311] | Human |  |
|  | Wu et al. (2014) | | Four independent experienced observers from the BRATS challenge | 1550 | 0.67 [0.6561; 0.6839] | Human |  |
|  | Total Human (I2 = 99.94%, p<0.001) | |  |  | 0.78 [0.69; 0.8603] |  |  |
|  | Total (I2 = 99.98%, p<0.001) | |  |  | 0.71 [0.635; 0.7751] |  | 0.014 |
| WT segmentation for HGG tumours |  | |  |  |  |  |  |
|  | Sanjuàn et al. (2013) | | Two independent experienced observers | 1408 | 0.7 [0.6932; 0.7068] | Automated |  |
|  | Eltayeb et al. (2019) | | Four independent experienced observers from the BRATS challenge | 3040 | 0.9 [0.8979; 0.9021] | Automated |  |
|  | Thiruvenkadam and Perumal (2016) | | Four independent experienced observers from the BRATS challenge | 3100 | 0.8 [0.7961; 0.8039] | Automated |  |
|  | Thiruvenkadam and Nagarajan (2020) | | Four independent experienced observers from the BRATS challenge | 3100 | 0.72 [0.7147; 0.7253] | Automated |  |
|  | Soltaninejad et al. (2020) | | Four independent experienced observers from the BRATS challenge | 3100 | 0.88 [0.8782; 0.8818] | Automated |  |
|  | Sriramakrishnan et al. (2019) | | Four independent experienced observers from the BRATS challenge | 3100 | 0.76 [0.7568; 0.7632] | Automated |  |
|  | Debnath et al. (2020) | | Four independent experienced observers from the BRATS challenge | 4650 | 0.95 [0.9494; 0.9506] | Automated |  |
|  | Kalaiselvi et al. (2019) | | Four independent experienced observers from the BRATS challenge | 3100 | 0.8 [0.7975; 0.8025] | Automated |  |
|  | Imtiaz et al. (2017) | | Four independent experienced observers from the BRATS challenge | 3100 | 0.86 [0.8586; 0.8614] | Automated |  |
|  | Wu et al. (2014) | | Four independent experienced observers from the BRATS challenge | 3100 | 0.61 [0.6037; 0.6163] | Automated |  |
|  | Rehman et al. (2019) | | Four independent experienced observers from the BRATS challenge | 2480 | 0.88 [0.878; 0.882] | Automated |  |
|  | Total Automated (I2 = 99.98%, p<0.001) | |  |  | 0.81 [0.7374; 0.8736] |  |  |
|  | Sanjuàn et al. (2013) | | Two independent experienced observers | 1408 | 0.87 [0.8648; 0.8752] | Human |  |
|  | Eltayeb et al. (2019) | | Four independent experienced observers from the BRATS challenge | 3040 | 0.88 [0.8793; 0.8807] | Human |  |
|  | Thiruvenkadam and Perumal (2016) | | Four independent experienced observers from the BRATS challenge | 3100 | 0.88 [0.8793; 0.8807] | Human |  |
|  | Thiruvenkadam and Nagarajan (2020) | | Four independent experienced observers from the BRATS challenge | 3100 | 0.88 [0.8793; 0.8807] | Human |  |
|  | Soltaninejad et al. (2017) | | Four independent experienced observers from the BRATS challenge | 3100 | 0.88 [0.8793; 0.8807] | Human |  |
|  | Sriramakrishnan et al. (2019) | | Four independent experienced observers from the BRATS challenge | 3100 | 0.88 [0.8793; 0.8807] | Human |  |
|  | Debnath et al. (2020) | | Four independent experienced observers from the BRATS challenge | 4650 | 0.88 [0.8794; 0.8806] | Human |  |
|  | Kalaiselvi et al. (2019) | | Four independent experienced observers from the BRATS challenge | 3100 | 0.88 [0.8793; 0.8807] | Human |  |
|  | Imtiaz et al. (2017) | | Four independent experienced observers from the BRATS challenge | 3100 | 0.88 [0.8793; 0.8807] | Human |  |
|  | Wu et al. (2014) | | Four independent experienced observers from the BRATS challenge | 3100 | 0.88 [0.8793; 0.8807] | Human |  |
|  | Rehman et al. (2019) | | Four independent experienced observers from the BRATS challenge | 2480 | 0.88 [0.8792; 0.8808] | Human |  |
|  | Total Human (I2 = 28.85%, p=0.17) | |  |  | 0.88 [0.8785; 0.8809] |  |  |
|  | Total (I2 = 99.97%, p<0.001) | |  |  | 0.84 [0.8071; 0.8775] |  | 0.015 |
| WT segmentation for LGG tumours |  | |  |  |  |  |  |
|  | Sanjuàn et al. (2013) | | Two independent experienced observers | 1584 | 0.72 [0.7166; 0.7234] | Automated |  |
|  | Eltayeb et al. (2019) | | Four independent experienced observers from the BRATS challenge | 1280 | 0.9 [0.8973; 0.9027] | Automated |  |
|  | Thiruvenkadam and Perumal (2016) | | Four independent experienced observers from the BRATS challenge | 1550 | 0.79 [0.7835; 0.7965] | Automated |  |
|  | Thiruvenkadam and Nagarajan (2020) | | Four independent experienced observers from the BRATS challenge | 1550 | 0.75 [0.743; 0.757] | Automated |  |
|  | Soltaninejad et al. (2017) | | Four independent experienced observers from the BRATS challenge | 1550 | 0.89 [0.888; 0.892] | Automated |  |
|  | Sriramakrishnan et al. (2019) | | Four independent experienced observers from the BRATS challenge | 1550 | 0.75 [0.745; 0.755] | Automated |  |
|  | Debnath et al. (2020) | | Four independent experienced observers from the BRATS challenge | 775 | 0.94 [0.9372; 0.9428] | Automated |  |
|  | Kalaiselvi et al. (2019) | | Four independent experienced observers from the BRATS challenge | 1550 | 0.81 [0.807; 0.813] | Automated |  |
|  | Wu et al. (2014) | | Four independent experienced observers from the BRATS challenge | 1550 | 0.43 [0.4195; 0.4405] | Automated |  |
|  | Rehman et al. (2019) | | Four independent experienced observers from the BRATS challenge | 775 | 0.92 [0.9179; 0.9221] | Automated |  |
|  | Total Automated (I2 = 99.96%, p<0.001) | |  |  | 0.79 [0.6835; 0.8966] |  |  |
|  | Sanjuàn et al. (2013) | | Two independent experienced observers | 1584 | 0.8 [0.7946; 0.8054] | Human |  |
|  | Eltayeb et al. (2019) | | Four independent experienced observers from the BRATS challenge | 1280 | 0.84 [0.8389; 0.8411] | Human |  |
|  | Thiruvenkadam and Perumal (2016) | | Four independent experienced observers from the BRATS challenge | 1550 | 0.84 [0.839; 0.841] | Human |  |
|  | Thiruvenkadam and Nagarajan (2020) | | Four independent experienced observers from the BRATS challenge | 1550 | 0.84 [0.839; 0.841] | Human |  |
|  | Soltaninejad et al. (2017) | | Four independent experienced observers from the BRATS challenge | 1550 | 0.84 [0.839; 0.841] | Human |  |
|  | Sriramakrishnan et al. (2019) | | Four independent experienced observers from the BRATS challenge | 1550 | 0.84 [0.839; 0.841] | Human |  |
|  | Debnath et al. (2020) | | Four independent experienced observers from the BRATS challenge | 775 | 0.84 [0.8386; 0.8414] | Human |  |
|  | Kalaiselvi et al. (2019) | | Four independent experienced observers from the BRATS challenge | 1550 | 0.84 [0.839; 0.841] | Human |  |
|  | Wu et al. (2014) | | Four independent experienced observers from the BRATS challenge | 1550 | 0.84 [0.839; 0.841] | Human |  |
|  | Rehman et al. (2019) | | Four independent experienced observers from the BRATS challenge | 775 | 0.84 [0.8386; 0.8414] | Human |  |
|  | Total Human (I2 = 95.68%, p<0.001) | |  |  | 0.84 [0.8273; 0.845] |  |  |
|  | Total (I2 = 99.93%, p<0.001) | |  |  | 0.81 [0.7637; 0.8624] |  | 0.33 |
| WT=Whole tumour; TC=Tumour core; ET=Enhancing tumour; DSC=Dice Score Coefficient; LGG=Low Grade Glioma; HGG=High Grade Glioma | |  |  |  |  |  |  |

# Supplementary Figure 1: Cumulative number of Traditional Machine Learning (TML) and Deep Learning (DL) studies from 2000-2021 included in this review


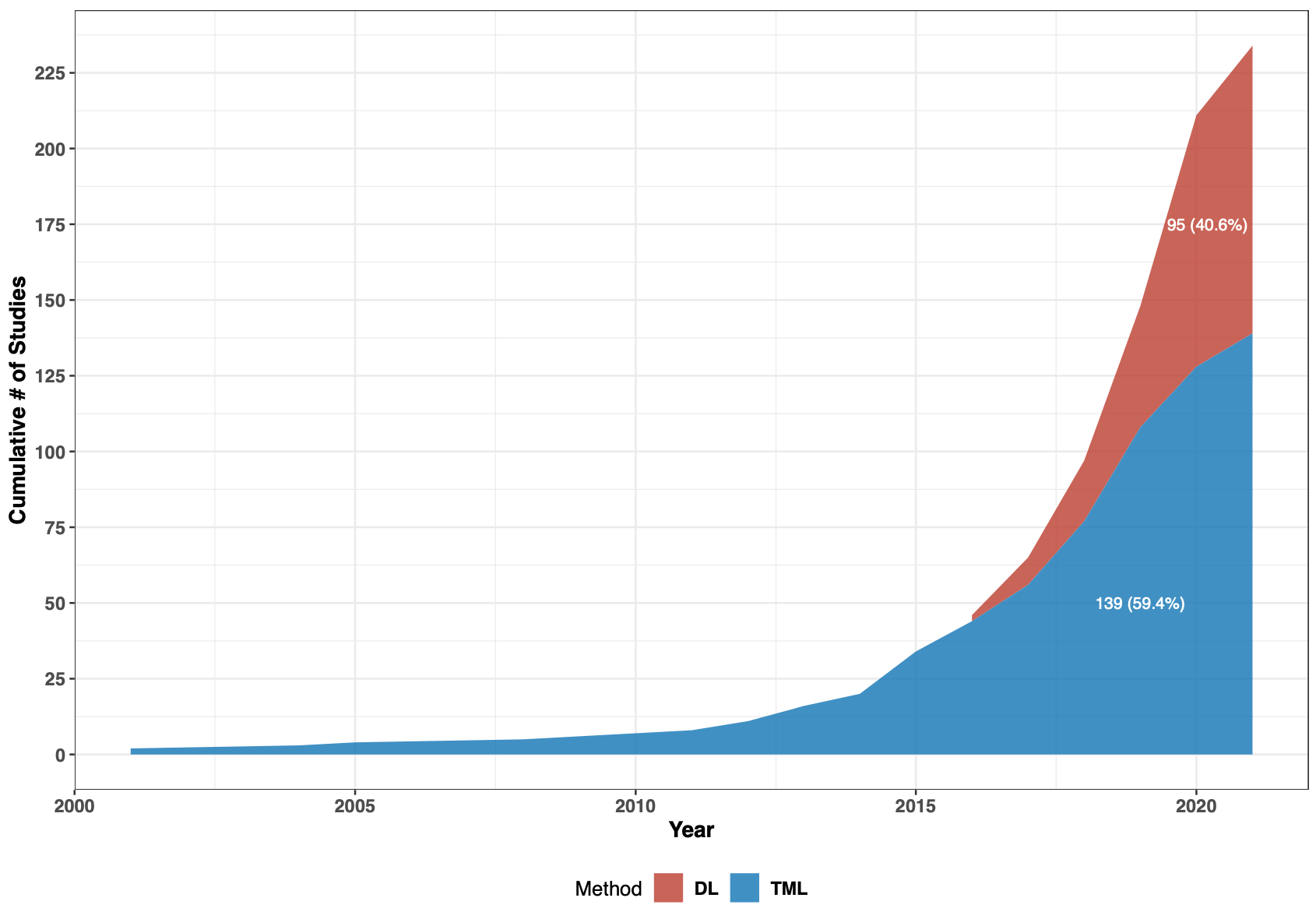


# Supplementary Figure 2: Venn Diagram of datasets utilised in included studies


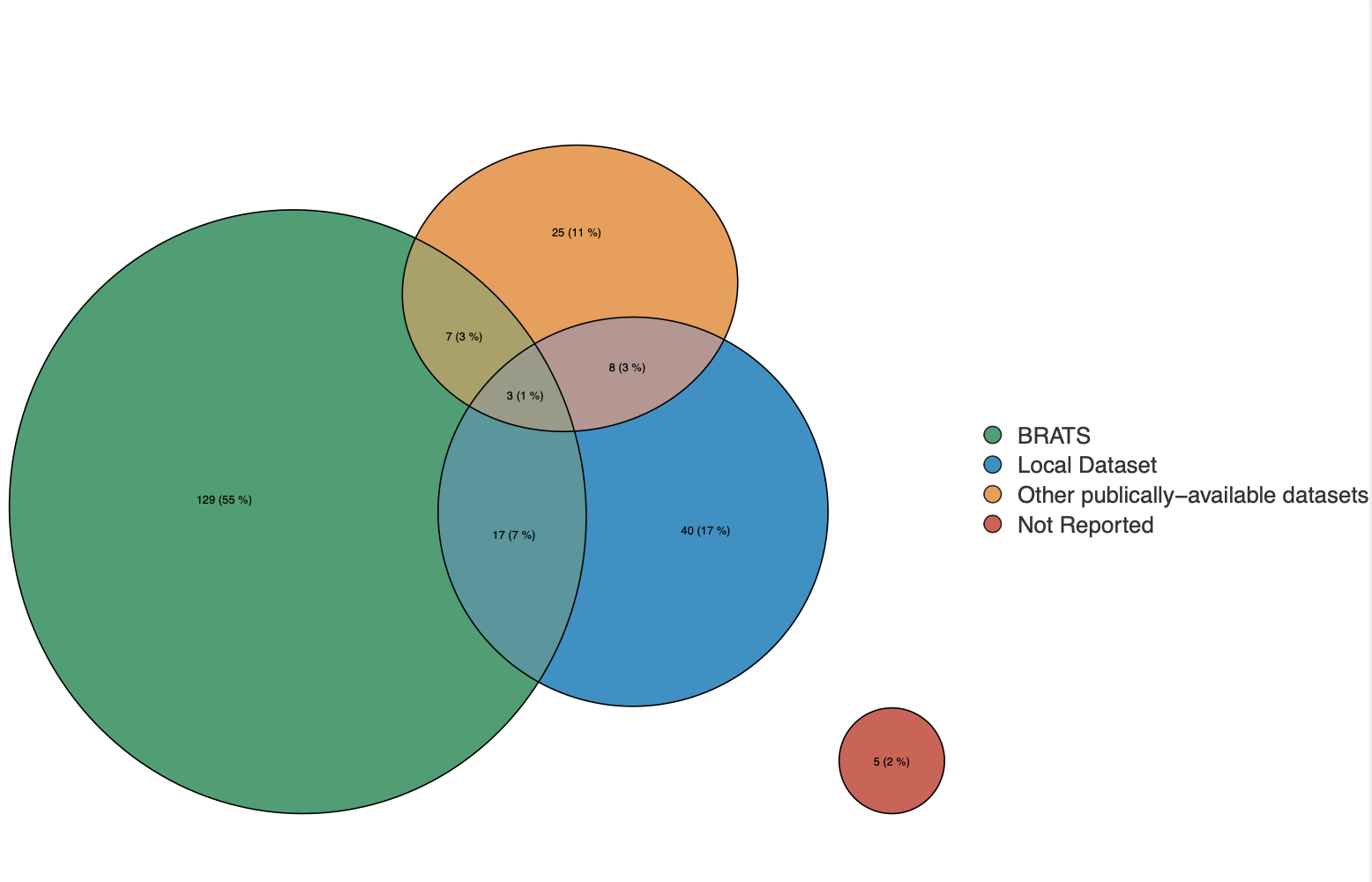


# Supplementary Figure 3: UpSet plot of interactions between the different types of tumours across included studies

# Supplementary Figure 4: UpSet plot of interactions between the different types of MRI modalities across included studies

# Supplementary Figure 5: UpSet plot of interactions between the different types of performance metrics utilised across included studies

# Supplementary Figure 6: UpSet plot of interactions between the different types of internal validation techniques utilised across included studies

# Supplementary Figure 7: Out-of-sample external validation for Traditional Machine Learning (TML) and Deep Learning (DL) of studies included


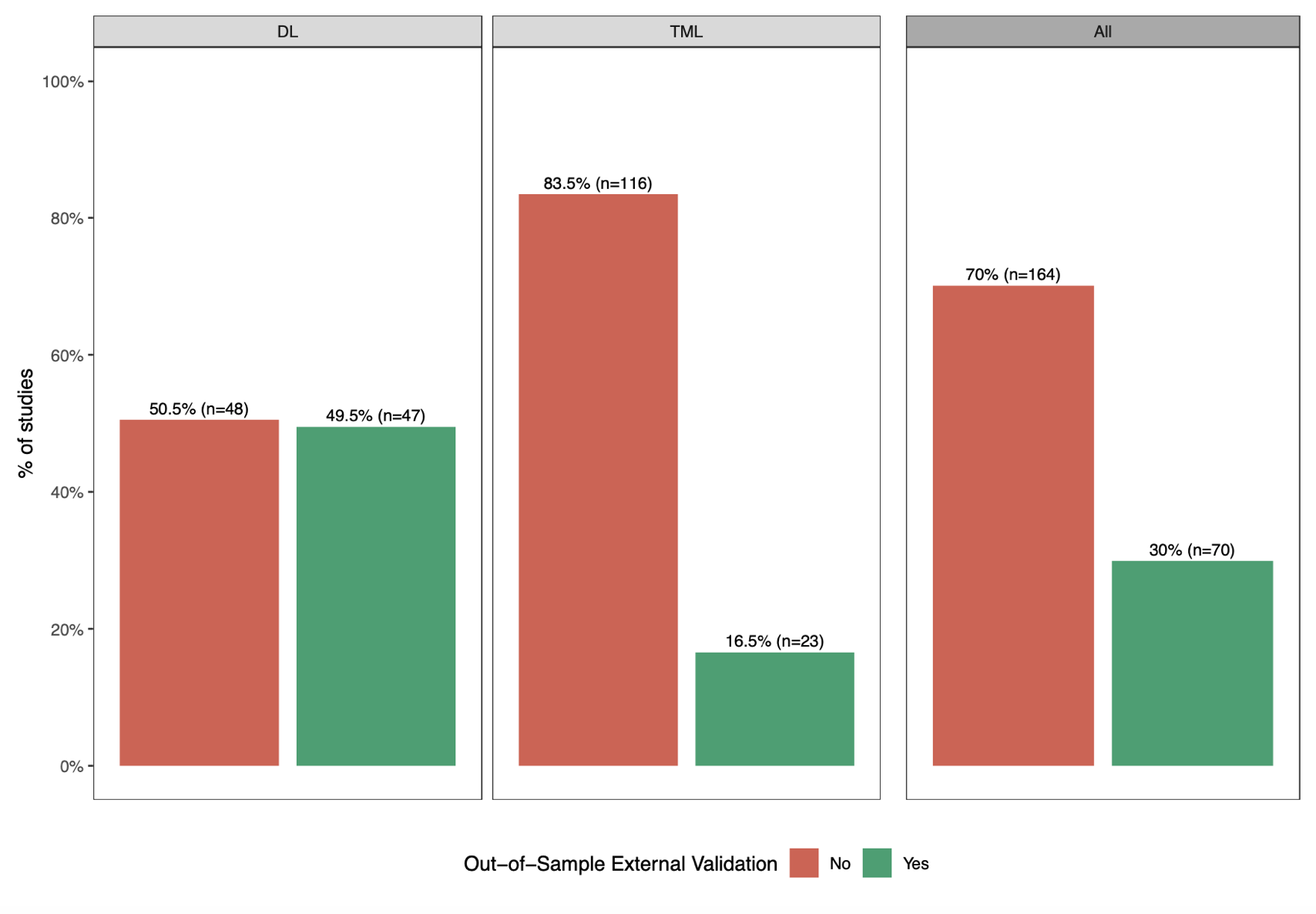


# Supplementary Figure 8: Computational (inference) time (second/MRI slice) for Traditional Machine Learning (TML) and Deep Learning (DL) segmentation for both semi-automated and fully automated techniques


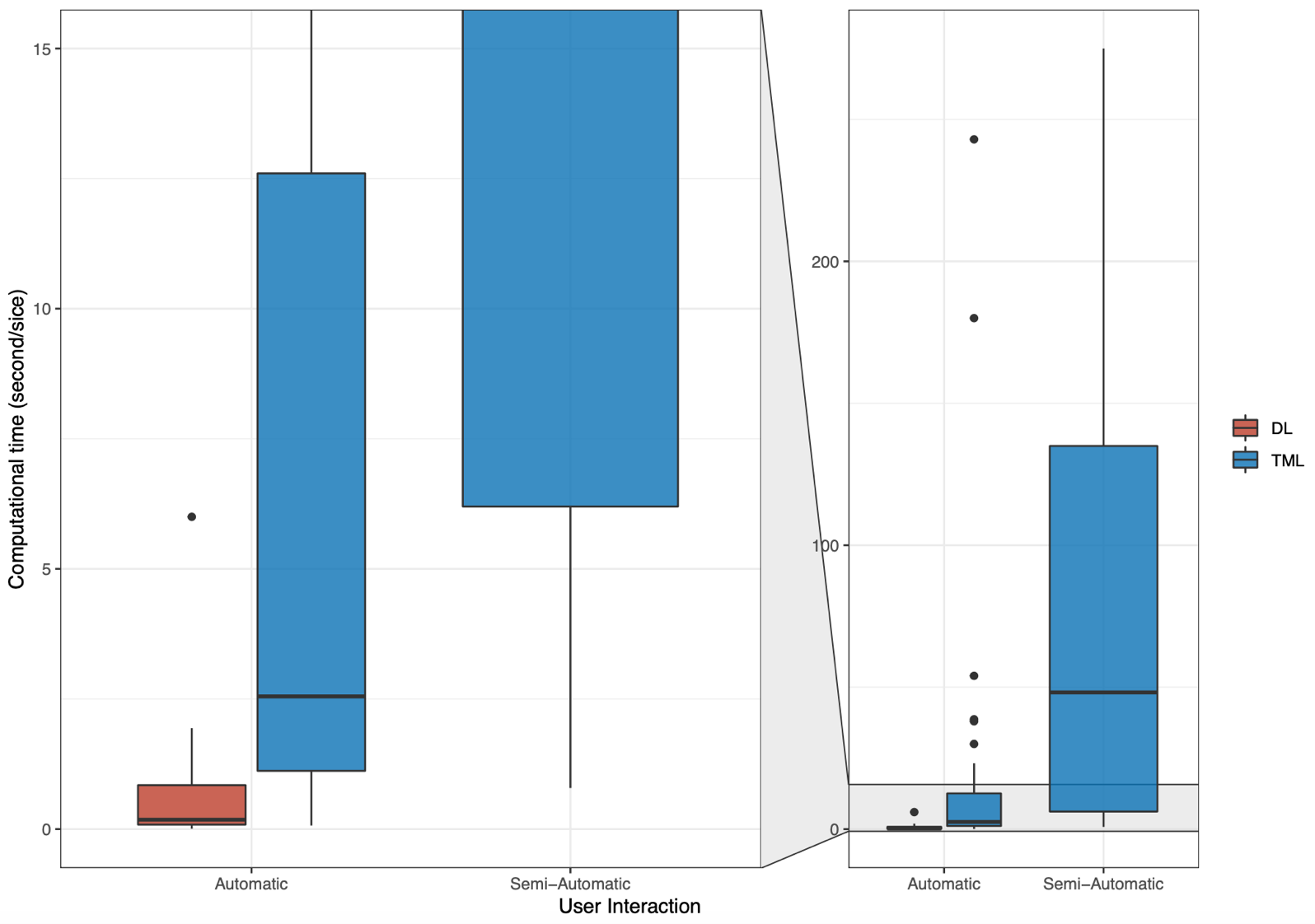


# Supplementary Figure 9: Summary of CLAIM reporting quality across included studies


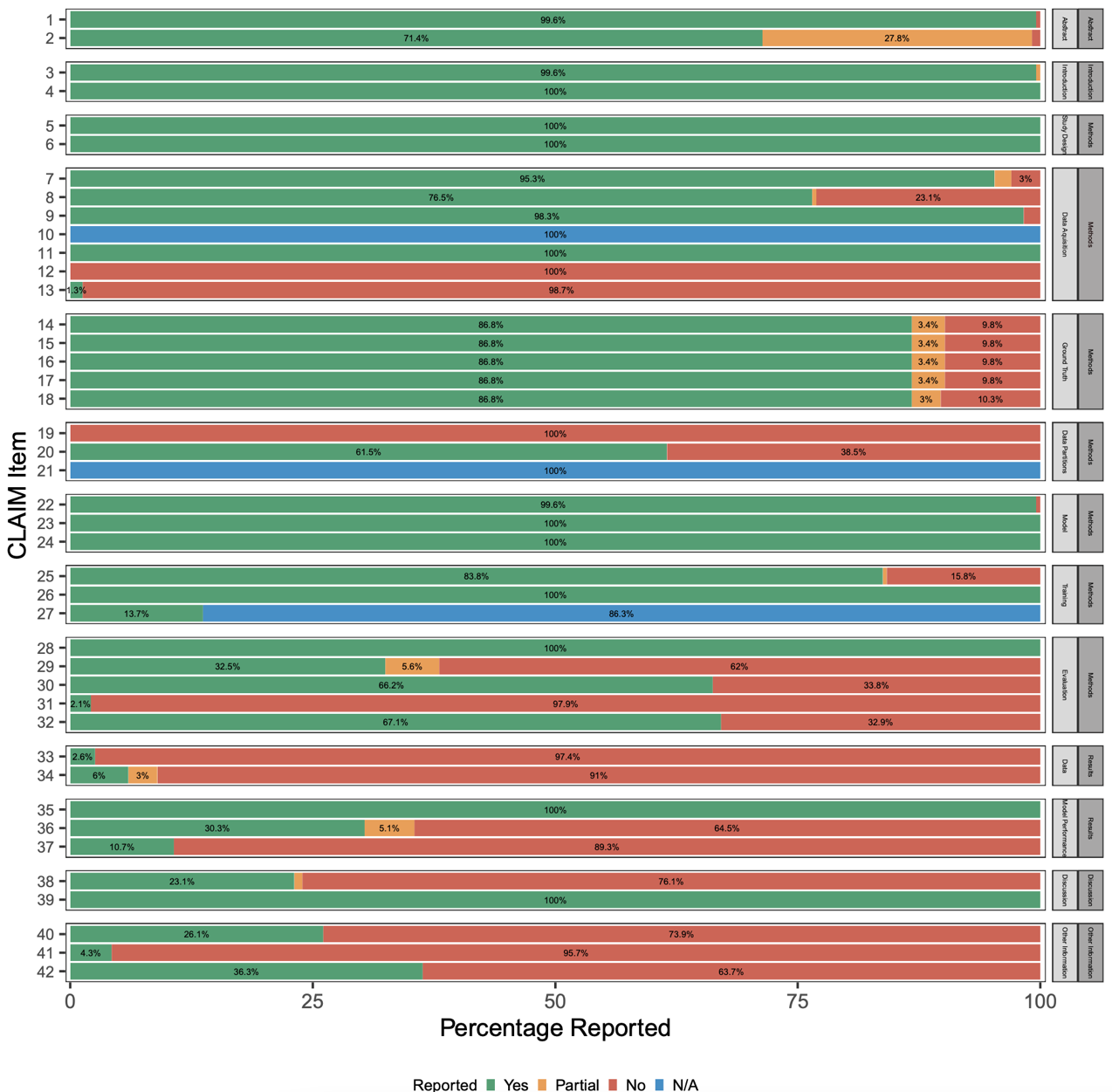


# Supplementary Figure 10: Summary of QUADAS-2 risk of bias and applicability assessment across included studies


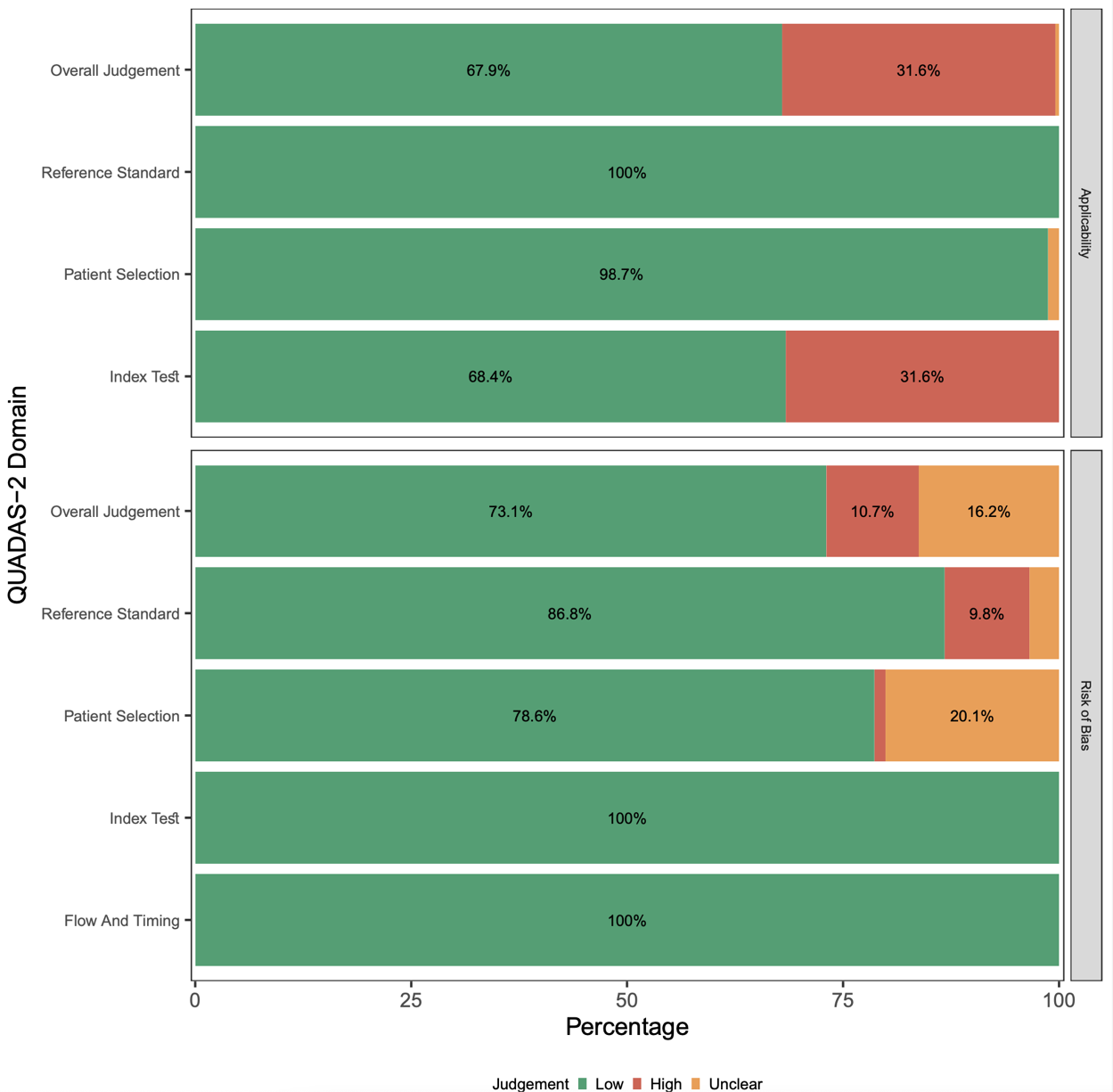


# References:

1. Fletcher-Heath LM, Hall LO, Goldgof DB, Murtagh FR. Automatic segmentation of non-enhancing brain tumors in magnetic resonance images. Artificial intelligence in medicine. 2001 Jan 1;21(1-3):43-63.
2. Kaus MR, Warfield SK, Nabavi A, Black PM, Jolesz FA, Kikinis R. Automated segmentation of MR images of brain tumors. Radiology. 2001 Feb;218(2):586-91.
3. Prastawa M, Bullitt E, Ho S, Gerig G. A brain tumor segmentation framework based on outlier detection. Medical image analysis. 2004 Sep 1;8(3):275-83.
4. Xie K, Yang J, Zhang ZG, Zhu YM. Semi-automated brain tumor and edema segmentation using MRI. European journal of radiology. 2005 Oct 1;56(1):12-9.
5. Corso JJ, Sharon E, Dube S, El-Saden S, Sinha U, Yuille A. Efficient multilevel brain tumor segmentation with integrated bayesian model classification. IEEE transactions on medical imaging. 2008 Apr 25;27(5):629-40.
6. Nie J, Xue Z, Liu T, Young GS, Setayesh K, Guo L, Wong ST. Automated brain tumor segmentation using spatial accuracy-weighted hidden Markov Random Field. Computerized Medical Imaging and Graphics. 2009 Sep 1;33(6):431-41.
7. Taheri S, Ong SH, Chong VF. Level-set segmentation of brain tumors using a threshold-based speed function. Image and Vision Computing. 2010 Jan 1;28(1):26-37.
8. Hsieh TM, Liu YM, Liao CC, Xiao F, Chiang IJ, Wong JM. Automatic segmentation of meningioma from non-contrasted brain MRI integrating fuzzy clustering and region growing. BMC medical informatics and decision making. 2011 Dec;11(1):1-2.
9. Hamamci A, Kucuk N, Karaman K, Engin K, Unal G. Tumor-cut: segmentation of brain tumors on contrast enhanced MR images for radiosurgery applications. IEEE transactions on medical imaging. 2011 Dec 26;31(3):790-804.
10. Sanjuán A, Price CJ, Mancini L, Josse G, Grogan A, Yamamoto AK, Geva S, Leff AP, Yousry TA, Seghier ML. Automated identification of brain tumors from single MR images based on segmentation with refined patient-specific priors. Frontiers in neuroscience. 2013 Dec 17;7:241.
11. Jiang J, Wu Y, Huang M, Yang W, Chen W, Feng Q. 3D brain tumor segmentation in multimodal MR images based on learning population-and patient-specific feature sets. Computerized Medical Imaging and Graphics. 2013 Oct 1;37(7-8):512-21.
12. Wu Y, Yang W, Jiang J, Li S, Feng Q, Chen W. Semi-automatic segmentation of brain tumors using population and individual information. Journal of digital imaging. 2013 Aug 1;26(4):786-96.
13. Kharrat A, BenMessaoud M, Abid M. Brain tumour diagnostic segmentation based on optimal texture features and support vector machine classifier. International Journal of Signal and Imaging Systems Engineering. 2014 Jan 1;7(2):65-74.
14. Tustison NJ, Shrinidhi KL, Wintermark M, Durst CR, Kandel BM, Gee JC, Grossman MC, Avants BB. Optimal symmetric multimodal templates and concatenated random forests for supervised brain tumor segmentation (simplified) with ANTsR. Neuroinformatics. 2015 Apr;13(2):209-25.
15. Wu W, Chen AY, Zhao L, Corso JJ. Brain tumor detection and segmentation in a CRF (conditional random fields) framework with pixel-pairwise affinity and superpixel-level features. International journal of computer assisted radiology and surgery. 2014 Mar;9(2):241-53.
16. Demirhan A, Törü M, Güler I. Segmentation of tumor and edema along with healthy tissues of brain using wavelets and neural networks. IEEE journal of biomedical and health informatics. 2014 Sep 26;19(4):1451-8.
17. Abdel-Maksoud E, Elmogy M, Al-Awadi R. Brain tumor segmentation based on a hybrid clustering technique. Egyptian Informatics Journal. 2015 Mar 1;16(1):71-81.
18. Ali H, Elmogy M, El-Daydamony E, Atwan A. Multi-resolution MRI brain image segmentation based on morphological pyramid and fuzzy c-mean clustering. Arabian Journal for Science and Engineering. 2015 Nov;40(11):3173-85.
19. Njeh I, Sallemi L, Ayed IB, Chtourou K, Lehericy S, Galanaud D, Hamida AB. 3D multimodal MRI brain glioma tumor and edema segmentation: a graph cut distribution matching approach. Computerized Medical Imaging and Graphics. 2015 Mar 1;40:108-19.
20. Juan-Albarracín J, Fuster-Garcia E, Manjon JV, Robles M, Aparici F, Martí-Bonmatí L, Garcia-Gomez JM. Automated glioblastoma segmentation based on a multiparametric structured unsupervised classification. PloS one. 2015 May 15;10(5):e0125143.
21. Bahadure NB, Ray AK, Thethi HP. Image analysis for MRI based brain tumor detection and feature extraction using biologically inspired BWT and SVM. International journal of biomedical imaging. 2017 Mar 6;2017.
22. Nabizadeh N, Kubat M. Brain tumors detection and segmentation in MR images: Gabor wavelet vs. statistical features. Computers & Electrical Engineering. 2015 Jul 1;45:286-301.
23. Dvorak P, Bartusek K, Kropatsch WG, Smékal Z. Automated multi-contrast brain pathological area extraction from 2D MR images. Journal of applied research and technology. 2015;13(1):58-69.
24. Szwarc P, Kawa J, Rudzki M, Pietka E. Automatic brain tumour detection and neovasculature assessment with multiseries MRI analysis. Computerized Medical Imaging and Graphics. 2015 Dec 1;46:178-90.
25. Steed TC, Treiber JM, Patel KS, Taich Z, White NS, Treiber ML, Farid N, Carter BS, Dale AM, Chen CC. Iterative probabilistic voxel labeling: automated segmentation for analysis of The Cancer Imaging Archive glioblastoma images. American journal of neuroradiology. 2015 Apr 1;36(4):678-85.
26. Akkus Z, Sedlar J, Coufalova L, Korfiatis P, Kline TL, Warner JD, Agrawal J, Erickson BJ. Semi-automated segmentation of pre-operative low grade gliomas in magnetic resonance imaging. Cancer Imaging. 2015 Dec;15(1):1-0.
27. Hasan AM, Meziane F, Aspin R, Jalab HA. Segmentation of brain tumors in MRI images using three-dimensional active contour without edge. Symmetry. 2016 Nov;8(11):132.
28. Ilunga-Mbuyamba E, Avina–Cervantes JG, Garcia–Perez A, de Jesus Romero–Troncoso R, Aguirre–Ramos H, Cruz–Aceves I, Chalopin C. Localized active contour model with background intensity compensation applied on automatic MR brain tumor segmentation. Neurocomputing. 2017 Jan 12;220:84-97.
29. Vishnuvarthanan G, Rajasekaran MP, Subbaraj P, Vishnuvarthanan A. An unsupervised learning method with a clustering approach for tumor identification and tissue segmentation in magnetic resonance brain images. Applied Soft Computing. 2016 Jan 1;38:190-212.
30. Thiruvenkadam K, Perumal N. Fully automatic method for segmentation of brain tumor from multimodal magnetic resonance images using wavelet transformation and clustering technique. International Journal of Imaging Systems and Technology. 2016 Dec;26(4):305-14.
31. Zhao L, Jia K. Multiscale CNNs for brain tumor segmentation and diagnosis. Computational and mathematical methods in medicine. 2016 Mar 16;2016.
32. Cordier N, Delingette H, Ayache N. A patch-based approach for the segmentation of pathologies: application to glioma labelling. IEEE transactions on medical imaging. 2015 Dec 11;35(4):1066-76.
33. Pereira S, Pinto A, Alves V, Silva CA. Brain tumor segmentation using convolutional neural networks in MRI images. IEEE transactions on medical imaging. 2016 Mar 4;35(5):1240-51.
34. Koley S, Sadhu AK, Mitra P, Chakraborty B, Chakraborty C. Delineation and diagnosis of brain tumors from post contrast T1-weighted MR images using rough granular computing and random forest. Applied Soft Computing. 2016 Apr 1;41:453-65.
35. Liu Y, Stojadinovic S, Hrycushko B, Wardak Z, Lu W, Yan Y, Jiang SB, Timmerman R, Abdulrahman R, Nedzi L, Gu X. Automatic metastatic brain tumor segmentation for stereotactic radiosurgery applications. Physics in Medicine & Biology. 2016 Nov 15;61(24):8440.
36. Li Y, Jia F, Qin J. Brain tumor segmentation from multimodal magnetic resonance images via sparse representation. Artificial intelligence in medicine. 2016 Oct 1;73:1-3.
37. Vishnuvarthanan G, Rajasekaran MP, Vishnuvarthanan NA, Prasath TA, Kannan M. Tumor detection in T1, T2, FLAIR and MPR brain images using a combination of optimization and fuzzy clustering improved by seed‐based region growing algorithm. International Journal of Imaging Systems and Technology. 2017 Mar;27(1):33-45.
38. Kamnitsas K, Ledig C, Newcombe VF, Simpson JP, Kane AD, Menon DK, Rueckert D, Glocker B. Efficient multi-scale 3D CNN with fully connected CRF for accurate brain lesion segmentation. Medical image analysis. 2017 Feb 1;36:61-78.
39. Havaei M, Davy A, Warde-Farley D, Biard A, Courville A, Bengio Y, Pal C, Jodoin PM, Larochelle H. Brain tumor segmentation with deep neural networks. Medical image analysis. 2017 Jan 1;35:18-31.
40. Soltaninejad M, Yang G, Lambrou T, Allinson N, Jones TL, Barrick TR, Howe FA, Ye X. Automated brain tumour detection and segmentation using superpixel-based extremely randomized trees in FLAIR MRI. International journal of computer assisted radiology and surgery. 2017 Feb 1;12(2):183-203.
41. Gupta N, Khanna P. A non-invasive and adaptive CAD system to detect brain tumor from T2-weighted MRIs using customized Otsu’s thresholding with prominent features and supervised learning. Signal Processing: Image Communication. 2017 Nov 1;59:18-26.
42. Anitha R, Raja DS. Segmentation of glioma tumors using convolutional neural networks. International Journal of Imaging Systems and Technology. 2017 Dec;27(4):354-60.
43. Cui S, Mao L, Xiong S. Brain tumor automatic segmentation using fully Convolutional networks. Journal of Medical Imaging and Health Informatics. 2017 Nov 1;7(7):1641-7.
44. Sasikanth S, Suresh Kumar S. Glioma tumor detection in brain MRI image using ANFIS‐based normalized graph cut approach. International Journal of Imaging Systems and Technology. 2018 Mar;28(1):64-71.
45. Imtiaz T, Rifat S, Fattah SA, Wahid KA. Automated brain tumor segmentation based on multi-planar superpixel level features extracted from 3D MR images. IEEE Access. 2019 Dec 23;8:25335-49.
46. Kaur T, Saini BS, Gupta S. A novel fully automatic multilevel thresholding technique based on optimized intuitionistic fuzzy sets and tsallis entropy for MR brain tumor image segmentation. Australasian physical & engineering sciences in medicine. 2018 Mar;41(1):41-58.
47. Rajinikanth V, Raja NS, Kamalanand K. Firefly algorithm assisted segmentation of tumor from brain MRI using Tsallis function and Markov random field. Journal of Control Engineering and Applied Informatics. 2017 Sep 29;19(3):97-106.
48. Rajinikanth V, Satapathy SC, Fernandes SL, Nachiappan S. Entropy based segmentation of tumor from brain MR images–a study with teaching learning based optimization. Pattern Recognition Letters. 2017 Jul 15;94:87-95.
49. Liu Y, Stojadinovic S, Hrycushko B, Wardak Z, Lau S, Lu W, Yan Y, Jiang SB, Zhen X, Timmerman R, Nedzi L. A deep convolutional neural network-based automatic delineation strategy for multiple brain metastases stereotactic radiosurgery. PloS one. 2017 Oct 6;12(10):e0185844.
50. Zhuge Y, Krauze AV, Ning H, Cheng JY, Arora BC, Camphausen K, Miller RW. Brain tumor segmentation using holistically nested neural networks in MRI images. Medical physics. 2017 Oct;44(10):5234-43.
51. Li Z, Wang Y, Yu J, Shi Z, Guo Y, Chen L, Mao Y. Low-grade glioma segmentation based on CNN with fully connected CRF. Journal of healthcare engineering. 2017 Mar;2017.
52. Selvapandian A, Manivannan K. Fusion based glioma brain tumor detection and segmentation using ANFIS classification. Computer methods and programs in biomedicine. 2018 Nov 1;166:33-8.
53. Raju AR, Suresh P, Rao RR. Bayesian HCS-based multi-SVNN: a classification approach for brain tumor segmentation and classification using Bayesian fuzzy clustering. Biocybernetics and Biomedical Engineering. 2018 Jan 1;38(3):646-60.
54. Essadike A, Ouabida E, Bouzid A. Brain tumor segmentation with Vander Lugt correlator based active contour. Computer methods and programs in biomedicine. 2018 Jul 1;160:103-17.
55. Kermi A, Andjouh K, Zidane F. Fully automated brain tumour segmentation system in 3D-MRI using symmetry analysis of brain and level sets. IET Image Processing. 2018 Oct 25;12(11):1964-71.
56. Pinto A, Pereira S, Rasteiro D, Silva CA. Hierarchical brain tumour segmentation using extremely randomized trees. Pattern Recognition. 2018 Oct 1;82:105-17.
57. Narayanan A, Rajasekaran MP, Zhang Y, Govindaraj V, Thiyagarajan A. Multi-channeled MR brain image segmentation: A novel double optimization approach combined with clustering technique for tumor identification and tissue segmentation. Biocybernetics and Biomedical Engineering. 2019 Apr 1;39(2):350-81.
58. Ma C, Luo G, Wang K. Concatenated and connected random forests with multiscale patch driven active contour model for automated brain tumor segmentation of MR images. IEEE transactions on medical imaging. 2018 Feb 13;37(8):1943-54.
59. Amin J, Sharif M, Yasmin M, Fernandes SL. Big data analysis for brain tumor detection: Deep convolutional neural networks. Future Generation Computer Systems. 2018 Oct 1;87:290-7.
60. Tong JJ, Zhang P, Weng YX, Zhu DH. Kernel sparse representation for MRI image analysis in automatic brain tumor segmentation. Frontiers of Information Technology & Electronic Engineering. 2018 Apr;19(4):471-80.
61. Laukamp KR, Thiele F, Shakirin G, Zopfs D, Faymonville A, Timmer M, Maintz D, Perkuhn M, Borggrefe J. Fully automated detection and segmentation of meningiomas using deep learning on routine multiparametric MRI. European radiology. 2019 Jan;29(1):124-32.
62. Lim KY, Mandava R. A multi-phase semi-automatic approach for multisequence brain tumor image segmentation. Expert Systems with Applications. 2018 Dec 1;112:288-300.
63. Szilagyi L, Iclănzan D, Kapas Z, Szabó Z, Győrfi Á, Lefkovits L. Low and high grade glioma segmentation in multispectral brain MRI data. Acta Universitatis Sapientiae, Informatica. 2018 Aug 1;10(1):110-32.
64. Abd-Ellah MK, Awad AI, Khalaf AA, Hamed HF. Two-phase multi-model automatic brain tumour diagnosis system from magnetic resonance images using convolutional neural networks. EURASIP Journal on Image and Video Processing. 2018 Dec;2018(1):1-0.
65. Angulakshmi M, Priya GL. Brain tumour segmentation from MRI using superpixels based spectral clustering. Journal of King Saud University-Computer and Information Sciences. 2018 Feb 1.
66. Perkuhn M, Stavrinou P, Thiele F, Shakirin G, Mohan M, Garmpis D, Kabbasch C, Borggrefe J. Clinical evaluation of a multiparametric deep learning model for glioblastoma segmentation using heterogeneous magnetic resonance imaging data from clinical routine. Investigative radiology. 2018 Nov;53(11):647.
67. Soltaninejad M, Yang G, Lambrou T, Allinson N, Jones TL, Barrick TR, Howe FA, Ye X. Supervised learning based multimodal MRI brain tumour segmentation using texture features from supervoxels. Computer methods and programs in biomedicine. 2018 Apr 1;157:69-84.
68. Saouli R, Akil M, Kachouri R. Fully automatic brain tumor segmentation using end-to-end incremental deep neural networks in MRI images. Computer methods and programs in biomedicine. 2018 Nov 1;166:39-49.
69. Naceur MB, Saouli R, Akil M, Kachouri R. Fully Automatic Brain Tumor Segmentation using End-To-End Incremental Deep Neural Networks in MRI images [Internet]. Vol. 166, Computer Methods and Programs in Biomedicine. Elsevier BV; 2018. p. 39–49.
70. Charron O, Lallement A, Jarnet D, Noblet V, Clavier JB, Meyer P. Automatic detection and segmentation of brain metastases on multimodal MR images with a deep convolutional neural network. Computers in biology and medicine. 2018 Apr 1;95:43-54.
71. Li Q, Gao Z, Wang Q, Xia J, Zhang H, Zhang H, Liu H, Li S. Glioma segmentation with a unified algorithm in multimodal MRI images. IEEE Access. 2018 Feb 21;6:9543-53.
72. Zaouche R, Belaid A, Aloui S, Solaiman B, Lecornu L, Salem DB, Tliba S. Semi-automatic method for low-grade gliomas segmentation in magnetic resonance imaging. IRBM. 2018 Apr 1;39(2):116-28.
73. Hussain S, Anwar SM, Majid M. Segmentation of glioma tumors in brain using deep convolutional neural network. Neurocomputing. 2018 Mar 22;282:248-61.
74. Iqbal S, Ghani MU, Saba T, Rehman A. Brain tumor segmentation in multi‐spectral MRI using convolutional neural networks (CNN). Microscopy research and technique. 2018 Apr;81(4):419-27.
75. Cui S, Mao L, Jiang J, Liu C, Xiong S. Automatic semantic segmentation of brain gliomas from MRI images using a deep cascaded neural network. Journal of healthcare engineering. 2018 Mar 19;2018.
76. Bonte S, Goethals I, Van Holen R. Machine learning based brain tumour segmentation on limited data using local texture and abnormality. Computers in biology and medicine. 2018 Jul 1;98:39-47.
77. Zhan T, Shen F, Hong X, Wang X, Chen Y, Lu Z, Yang G. A glioma segmentation method using cotraining and superpixel-based spatial and clinical constraints. IEEE Access. 2018 Oct 4;6:57113-22.
78. Virupakshappa, Amarapur B. Computer-aided diagnosis applied to MRI images of brain tumor using cognition based modified level set and optimized ANN classifier [Internet]. Vol. 79, Multimedia Tools and Applications. Springer Science and Business Media LLC; 2018. p. 3571–99.
79. Zhao X, Wu Y, Song G, Li Z, Zhang Y, Fan Y. A deep learning model integrating FCNNs and CRFs for brain tumor segmentation. Medical image analysis. 2018 Jan 1;43:98-111.
80. Zhao Z, Yang G, Lin Y, Pang H, Wang M. Automated glioma detection and segmentation using graphical models. PloS one. 2018 Aug 21;13(8):e0200745.
81. Jijja A, Rai D. Efficient MRI segmentation and detection of brain tumor using convolutional neural network. Int. J. Adv. Comput. Sci. Appl. 2019;10(4):536-41.
82. Natarajan A, Kumarasamy S. Efficient segmentation of brain tumor using FL-SNM with a metaheuristic approach to optimization. Journal of medical systems. 2019 Feb;43(2):1-4.
83. Chaudhari A, Kulkarni J. Semi-automatic unsupervised MR brain tumour segmentation using a simple Bayesian Framework. The Imaging Science Journal. 2019 Nov 17;67(8):434-46.
84. Hachemi B, Chama Z, Alim‐Ferhat F, Lamini ES, Abderrahmane A, Anani M, Choquet C. Fully automatic multisegmentation approach for magnetic resonance imaging brain tumor detection using improved region‐growing and quasi‐Monte Carlo‐expectation maximization algorithm. International Journal of Imaging Systems and Technology. 2020 Mar;30(1):104-11.
85. Sheela CJ, Suganthi G. Automatic brain tumor segmentation from MRI using greedy snake model and fuzzy C-means optimization. Journal of King Saud University-Computer and Information Sciences. 2019 Apr 11.
86. Zhang C, Shen X, Cheng H, Qian Q. Brain tumor segmentation based on hybrid clustering and morphological operations. International journal of biomedical imaging. 2019 Apr 9;2019.
87. Grøvik E, Yi D, Iv M, Tong E, Rubin D, Zaharchuk G. Deep learning enables automatic detection and segmentation of brain metastases on multisequence MRI. Journal of Magnetic Resonance Imaging. 2020 Jan;51(1):175-82.
88. Eltayeb EN, Salem NM, Al-Atabany W. Automated brain tumor segmentation from multi-slices FLAIR MRI images. Bio-medical materials and engineering. 2019 Jan 1;30(4):449-62.
89. Nagarathinam E, Ponnuchamy T. Image registration‐based brain tumor detection and segmentation using ANFIS classification approach. International Journal of Imaging Systems and Technology. 2019 Dec;29(4):510-7.
90. Wang G, Li W, Ourselin S, Vercauteren T. Automatic brain tumor segmentation based on cascaded convolutional neural networks with uncertainty estimation. Frontiers in computational neuroscience. 2019 Aug 13;13:56.
91. Li H, Li A, Wang M. A novel end-to-end brain tumor segmentation method using improved fully convolutional networks. Computers in biology and medicine. 2019 May 1;108:150-60.
92. Amin J, Sharif M, Raza M, Saba T, Anjum MA. Brain tumor detection using statistical and machine learning method. Computer methods and programs in biomedicine. 2019 Aug 1;177:69-79.
93. Sun J, Chen W, Peng S, Liu B. DRRNet: Dense residual refine networks for automatic brain tumor segmentation. Journal of medical systems. 2019 Jul;43(7):1-9.
94. Chang J, Zhang L, Gu N, Zhang X, Ye M, Yin R, Meng Q. A mix-pooling CNN architecture with FCRF for brain tumor segmentation. Journal of Visual Communication and Image Representation. 2019 Jan 1;58:316-22.
95. Tong J, Zhao Y, Zhang P, Chen L, Jiang L. MRI brain tumor segmentation based on texture features and kernel sparse coding. Biomedical Signal Processing and Control. 2019 Jan 1;47:387-92.
96. Shapey J, Wang G, Dorent R, Dimitriadis A, Li W, Paddick I, Kitchen N, Bisdas S, Saeed SR, Ourselin S, Bradford R. An artificial intelligence framework for automatic segmentation and volumetry of vestibular schwannomas from contrast-enhanced T1-weighted and high-resolution T2-weighted MRI. Journal of neurosurgery. 2019 Dec 6;134(1):171-9.
97. Zhao J, Meng Z, Wei L, Sun C, Zou Q, Su R. Supervised brain tumor segmentation based on gradient and context-sensitive features. Frontiers in neuroscience. 2019 Mar 14;13:144.
98. Dogra J, Jain S, Sood M. Glioma extraction from MR images employing gradient based kernel selection graph cut technique. The visual computer. 2020 May;36(5):875-91.
99. Hu K, Gan Q, Zhang Y, Deng S, Xiao F, Huang W, Cao C, Gao X. Brain tumor segmentation using multi-cascaded convolutional neural networks and conditional random field. IEEE Access. 2019 Jul 8;7:92615-29.
100. Na L, Zhiyong X, Tianqi D, Kai R. Automated brain tumor segmentation from multimodal MRI data based on Tamura texture feature and an ensemble SVM classifier. International Journal of Intelligent Computing and Cybernetics. 2019 Nov 11.
101. Wang L, Wang S, Chen R, Qu X, Chen Y, Huang S, Liu C. Nested dilation networks for brain tumor segmentation based on magnetic resonance imaging. Frontiers in neuroscience. 2019 Apr 5;13:285.
102. Thaha MM, Kumar KP, Murugan BS, Dhanasekeran S, Vijayakarthick P, Selvi AS. Brain tumor segmentation using convolutional neural networks in MRI images. Journal of medical systems. 2019 Sep;43(9):1-0.
103. Razzak MI, Imran M, Xu G. Efficient brain tumor segmentation with multiscale two-pathway-group conventional neural networks. IEEE journal of biomedical and health informatics. 2018 Oct 4;23(5):1911-9.
104. Sriramakrishnan P, Kalaiselvi T, Rajeswaran R. Modified local ternary patterns technique for brain tumour segmentation and volume estimation from MRI multi-sequence scans with GPU CUDA machine. Biocybernetics and Biomedical Engineering. 2019 Apr 1;39(2):470-87.
105. Kumar P, VijayKumar B. Brain tumor MRI segmentation and classification using ensemble classifier. International Journal of Recent Technology and Engineering (IJRTE). 2019 Jun;8(1S4).
106. Mlynarski P, Delingette H, Criminisi A, Ayache N. 3D convolutional neural networks for tumor segmentation using long-range 2D context. Computerized Medical Imaging and Graphics. 2019 Apr 1;73:60-72.
107. Mallick PK, Ryu SH, Satapathy SK, Mishra S, Nguyen GN, Tiwari P. Brain MRI image classification for cancer detection using deep wavelet autoencoder-based deep neural network. IEEE Access. 2019 Mar 15;7:46278-87.
108. Sun R, Wang K, Guo L, Yang C, Chen J, Ti Y, Sa Y. A potential field segmentation based method for tumor segmentation on multi-parametric MRI of glioma cancer patients. BMC medical imaging. 2019 Dec;19(1):1-9.
109. Janardhanaprabhu S, Malathi V. Brain tumor detection using depth-first search tree segmentation. Journal of medical systems. 2019 Aug;43(8):1-2.
110. Tchoketch Kebir S, Mekaoui S, Bouhedda M. A fully automatic methodology for MRI brain tumour detection and segmentation. The Imaging Science Journal. 2019 Jan 2;67(1):42-62.
111. Meghana S, Amulya P, Manisha A, Rajarajeswari P. A Deep Learning Approach For Brain Tumor Segmentation Using Convolution Neural Network. International Journal of Scientific & Technology Research. 2019 Dec;8(12):1697-1702.
112. Iqbal S, Ghani Khan MU, Saba T, Mehmood Z, Javaid N, Rehman A, Abbasi R. Deep learning model integrating features and novel classifiers fusion for brain tumor segmentation. Microscopy research and technique. 2019 Aug;82(8):1302-15.
113. Alagarsamy S, Kamatchi K, Govindaraj V, Zhang YD, Thiyagarajan A. Multi-channeled MR brain image segmentation: A new automated approach combining BAT and clustering technique for better identification of heterogeneous tumors. Biocybernetics and Biomedical Engineering. 2019 Oct 1;39(4):1005-35.
114. Shivhare SN, Kumar N, Singh N. A hybrid of active contour model and convex hull for automated brain tumor segmentation in multimodal MRI. Multimedia Tools and Applications. 2019 Dec;78(24):34207-29.
115. Nema S, Dudhane A, Murala S, Naidu S. RescueNet: An unpaired GAN for brain tumor segmentation. Biomedical Signal Processing and Control. 2020 Jan 1;55:101641.
116. Peng S, Chen W, Sun J, Liu B. Multi‐scale 3d u‐nets: an approach to automatic segmentation of brain tumor. International Journal of Imaging Systems and Technology. 2020 Mar;30(1):5-17.
117. Kalaiselvi T, Kumarashankar P, Sriramakrishnan P. Three-phase automatic brain tumor diagnosis system using patches based updated run length region growing technique. Journal of digital imaging. 2020 Apr;33(2):465-79.
118. Yang T, Song J, Li L. A deep learning model integrating SK-TPCNN and random forests for brain tumor segmentation in MRI. Biocybernetics and Biomedical Engineering. 2019 Jul 1;39(3):613-23.
119. Virupakshappa, Basavaraj A. Brain MRI segmentation using initial contour KPCM and optimal speed function for improved level set method [Internet]. Vol. 9, Health and Technology. Springer Science and Business Media LLC; 2019. p. 701–13.
120. Wu Y, Zhao Z, Wu W, Lin Y, Wang M. Automatic glioma segmentation based on adaptive superpixel. BMC medical imaging. 2019 Dec;19(1):1-4.
121. Wang Y, Li C, Zhu T, Zhang J. Multimodal brain tumor image segmentation using WRN-PPNet. Computerized Medical Imaging and Graphics. 2019 Jul 1;75:56-65.
122. Rehman ZU, Naqvi SS, Khan TM, Khan MA, Bashir T. Fully automated multi-parametric brain tumour segmentation using superpixel based classification. Expert systems with applications. 2019 Mar 15;118:598-613.
123. Kharrat A, Neji M. A System for Brain Image Segmentation and Classification Based on Three-Dimensional Convolutional Neural Network. Computación y Sistemas. 2020 Jul 12;24(4).
124. Rehman A, Khan MA, Saba T, Mehmood Z, Tariq U, Ayesha N. Microscopic brain tumor detection and classification using 3D CNN and feature selection architecture. Microscopy Research and Technique. 2021 Jan;84(1):133-49.
125. Khosravanian A, Rahmanimanesh M, Keshavarzi P, Mozaffari S. Fast level set method for glioma brain tumor segmentation based on superpixel fuzzy clustering and lattice boltzmann method. Computer Methods and Programs in Biomedicine. 2021 Jan 1;198:105809.
126. Srinivas B, Sasibhushana Rao G. Segmentation of Multi-Modal MRI Brain Tumor Sub-Regions Using Deep Learning. Journal of Electrical Engineering & Technology. 2020 Jul;15:1899-909.
127. Yogananda CG, Shah BR, Vejdani-Jahromi M, Nalawade SS, Murugesan GK, Yu FF, Pinho MC, Wagner BC, Emblem KE, Bjørnerud A, Fei B. A Fully automated deep learning network for brain tumor segmentation. Tomography. 2020 Jun;6(2):186-93.
128. Zhou C, Ding C, Wang X, Lu Z, Tao D. One-pass multi-task networks with cross-task guided attention for brain tumor segmentation. IEEE Transactions on Image Processing. 2020 Feb 19;29:4516-29.
129. Kumar DM, Satyanarayana D, Prasad MG. MRI brain tumor detection using optimal possibilistic fuzzy C-means clustering algorithm and adaptive k-nearest neighbor classifier. Journal of Ambient Intelligence and Humanized Computing. 2021 Feb;12(2):2867-80.
130. Chen G, Li Q, Shi F, Rekik I, Pan Z. RFDCR: Automated brain lesion segmentation using cascaded random forests with dense conditional random fields. NeuroImage. 2020 May 1;211:116620.
131. Chen H, Qin Z, Ding Y, Tian L, Qin Z. Brain tumor segmentation with deep convolutional symmetric neural network. Neurocomputing. 2020 Jun 7;392:305-13.
132. Tjahyaningtijas HP, Nugroho AK, Angkoso CV, Purnama IK, Purnomo MH. Automatic segmentation on glioblastoma brain tumor magnetic resonance imaging using modified u-net. EMITTER International Journal of Engineering Technology. 2020 Jun 2;8(1):161-77.
133. Khan H, Shah PM, Shah MA, ul Islam S, Rodrigues JJ. Cascading handcrafted features and Convolutional Neural Network for IoT-enabled brain tumor segmentation. Computer Communications. 2020 Mar 1;153:196-207.
134. Zhang J, Jiang Z, Dong J, Hou Y, Liu B. Attention gate resU-Net for automatic MRI brain tumor segmentation. IEEE Access. 2020 Mar 24;8:58533-45.
135. Zhang J, Lv X, Zhang H, Liu B. AResU-Net: Attention residual U-net for brain tumor segmentation. Symmetry. 2020 May;12(5):721.
136. Xue J, Wang B, Ming Y, Liu X, Jiang Z, Wang C, Liu X, Chen L, Qu J, Xu S, Tang X. Deep learning–based detection and segmentation-assisted management of brain metastases. Neuro-oncology. 2020 Apr 15;22(4):505-14.
137. Sun J, Peng Y, Guo Y, Li D. Segmentation of the multimodal brain tumor image used the multi-pathway architecture method based on 3D FCN. Neurocomputing. 2021 Jan 29;423:34-45.
138. Liu J, Liu H, Tang Z, Gui W, Ma T, Gong S, Gao Q, Xie Y, Niyoyita JP. IOUC-3DSFCNN: Segmentation of brain tumors via IOU constraint 3D symmetric full convolution network with multimodal auto-context. Scientific reports. 2020 Apr 10;10(1):1-5.
139. Chaudhary J, Rani R, Kamboj A. Deep learning-based approach for segmentation of glioma sub-regions in MRI. International Journal of Intelligent Computing and Cybernetics. 2020 Jul 21.
140. Laukamp KR, Pennig L, Thiele F, Reimer R, Görtz L, Shakirin G, Zopfs D, Timmer M, Perkuhn M, Borggrefe J. Automated meningioma segmentation in multiparametric MRI. Clinical neuroradiology. 2021 Jun;31(2):357-66.
141. Thiruvenkadam K, Nagarajan K. Fully automatic brain tumor extraction and tissue segmentation from multimodal MRI brain images. International Journal of Imaging Systems and Technology. 2021 Mar;31(1):336-50.
142. Gupta KK, Dhanda N, Kumar U. A Novel Hybrid Method for Segmentation and Analysis of Brain MRI for Tumor Diagnosis. Advances in Science, Technology and Engineering Systems Journal. 2020;5(3):16-27.
143. Yepuganti K, Saladi S, Narasimhulu CV. Segmentation of tumor using PCA based modified fuzzy C means algorithms on MR brain images. International Journal of Imaging Systems and Technology. 2020 Dec;30(4):1337-45.
144. Bousabarah K, Ruge M, Brand JS, Hoevels M, Rueß D, Borggrefe J, Hokamp NG, Visser-Vandewalle V, Maintz D, Treuer H, Kocher M. Deep convolutional neural networks for automated segmentation of brain metastases trained on clinical data. Radiation Oncology. 2020 Dec;15(1):1-9.
145. Ejaz K, Rahim MS, Bajwa UI, Chaudhry H, Rehman A, Ejaz F. Hybrid segmentation method with confidence region detection for tumor identification. IEEE Access. 2020 Aug 13;9:35256-78.
146. Pennig L, Hoyer UC, Goertz L, Shahzad R, Persigehl T, Thiele F, Perkuhn M, Ruge MI, Kabbasch C, Borggrefe J, Caldeira L. Primary central nervous system lymphoma: clinical evaluation of automated segmentation on multiparametric MRI using deep learning. Journal of Magnetic Resonance Imaging. 2021 Jan;53(1):259-68.
147. Ait Mohamed L, Cherfa A, Cherfa Y, Belkhamsa N, Alim‐Ferhat F. Hybrid method combining superpixel, supervised learning, and random walk for glioma segmentation. International Journal of Imaging Systems and Technology. 2021 Mar;31(1):288-301.
148. Ali M, Gilani SO, Waris A, Zafar K, Jamil M. Brain Tumour Image Segmentation Using Deep Networks. IEEE Access. 2020 Aug 20;8:153589-98.
149. Katouli M, Rahmani AE. Brain Tumor Diagnosis in MRI Images Using Image Processing Techniques and Pixel-Based Clustering. Traitement du Signal. 2020 Apr 1;37(2).
150. Ben naceur M, Akil M, Saouli R, Kachouri R. Fully automatic brain tumor segmentation with deep learning-based selective attention using overlapping patches and multi-class weighted cross-entropy [Internet]. Vol. 63, Medical Image Analysis. Elsevier BV; 2020. p. 101692.
151. Sharif M, Amin J, Raza M, Anjum MA, Afzal H, Shad SA. Brain tumor detection based on extreme learning. Neural Computing and Applications. 2020 Jan 11:1-3.
152. Aboelenein NM, Songhao P, Koubaa A, Noor A, Afifi A. HTTU-Net: hybrid two track U-net for automatic brain tumor segmentation. IEEE Access. 2020 May 29;8:101406-15.
153. Zaihani NH, Roslan R, Ibrahim Z, Samah KA. Automated segmentation and detection of T1-weighted magnetic resonance imaging brain images of glioma brain tumor. Bulletin of Electrical Engineering and Informatics. 2020 Jun 1;9(3):1032-7.
154. Hassen OA, Abter SO, Abdulhussein AA, Darwish SM, Ibrahim YM, Sheta W. Nature-Inspired Level Set Segmentation Model for 3D-MRI Brain Tumor Detection. CMC-Computers Materials & Continua. 2021 Jan 1;68(1):961-81.
155. Chithra PL, Dheepa G. Di‐phase midway convolution and deconvolution network for brain tumor segmentation in MRI images. International Journal of Imaging Systems and Technology. 2020 Sep;30(3):674-86.
156. Kao PY, Shailja S, Jiang J, Zhang A, Khan A, Chen JW, Manjunath BS. Improving patch-based convolutional neural networks for MRI brain tumor segmentation by leveraging location information. Frontiers in neuroscience. 2020 Jan 24;13:1449.
157. Pitchai R, Madhu Babu C, Supraja P, Challa MK. Cerebrum Tumor Segmentation of High Resolution Magnetic Resonance Images Using 2D-Convolutional Network with Skull Stripping. Neural Processing Letters. 2021 Aug;53(4):2567-80.
158. Zeineldin RA, Karar ME, Coburger J, Wirtz CR, Burgert O. DeepSeg: deep neural network framework for automatic brain tumor segmentation using magnetic resonance FLAIR images. International journal of computer assisted radiology and surgery. 2020 Jun;15(6):909-20.
159. Alqazzaz S, Sun X, Yang X, Nokes L. Automated brain tumor segmentation on multi-modal MR image using SegNet. Computational Visual Media. 2019 Jun;5(2):209-19.
160. Al-qazzaz S, Sun X, Yang H, Yang Y, Xu R, Nokes L, Yang X. Image classification-based brain tumour tissue segmentation. Multimedia Tools and Applications. 2021 Jan;80(1):993-1008.
161. Sathish P, Elango NM. Gaussian hybrid fuzzy clustering and radial basis neural network for automatic brain tumor classification in MRI images. Evolutionary Intelligence. 2020 Jun 14:1-9.
162. Banerjee S, Mitra S. Novel volumetric sub-region segmentation in brain tumors. Frontiers in computational neuroscience. 2020 Jan 24;14:3.
163. Tripathi S, Verma A, Sharma N. Automatic segmentation of brain tumour in MR images using an enhanced deep learning approach. Computer Methods in Biomechanics and Biomedical Engineering: Imaging & Visualization. 2021 Mar 4;9(2):121-30.
164. Kanniappan S, Samiayya D, Vincent PM DR, Srinivasan K, Jayakody DN, Reina DG, Inoue A. An Efficient Hybrid Fuzzy-Clustering Driven 3D-Modeling of Magnetic Resonance Imagery for Enhanced Brain Tumor Diagnosis. Electronics. 2020 Mar;9(3):475.
165. Debnath S, Talukdar FA, Islam M. Combination of contrast enhanced fuzzy c-means (CEFCM) clustering and pixel based voxel mapping technique (PBVMT) for three dimensional brain tumour detection. Journal of Ambient Intelligence and Humanized Computing. 2021 Feb;12(2):2421-33.
166. Baid U, Talbar S, Rane S, Gupta S, Thakur MH, Moiyadi A, Sable N, Akolkar M, Mahajan A. A novel approach for fully automatic intra-tumor segmentation with 3D U-Net architecture for gliomas. Frontiers in computational neuroscience. 2020 Feb 18;14:10.
167. Hu X, Luo W, Hu J, Guo S, Huang W, Scott MR, Wiest R, Dahlweid M, Reyes M. Brain SegNet: 3D local refinement network for brain lesion segmentation. BMC medical imaging. 2020 Dec;20(1):1-0.
168. Zhou Z, He Z, Jia Y. AFPNet: A 3D fully convolutional neural network with atrous-convolution feature pyramid for brain tumor segmentation via MRI images. Neurocomputing. 2020 Aug 18;402:235-44.
169. Zhou Z, He Z, Shi M, Du J, Chen D. 3D dense connectivity network with atrous convolutional feature pyramid for brain tumor segmentation in magnetic resonance imaging of human heads. Computers in biology and medicine. 2020 Jun 1;121:103766.
170. Barzegar Z, Jamzad M. A reliable ensemble-based classification framework for glioma brain tumor segmentation. Signal, Image and Video Processing. 2020 Nov;14:1591-9.
171. Győrfi Á, Szilágyi L, Kovács L. A Fully Automatic Procedure for Brain Tumor Segmentation from Multi-Spectral MRI Records Using Ensemble Learning and Atlas-Based Data Enhancement. Applied Sciences. 2021 Jan;11(2):564.
172. Di Ieva A, Russo C, Liu S, Jian A, Bai MY, Qian Y, Magnussen JS. Application of deep learning for automatic segmentation of brain tumors on magnetic resonance imaging: a heuristic approach in the clinical scenario. Neuroradiology. 2021 Jan 26:1-0.
173. Biratu ES, Schwenker F, Debelee TG, Kebede SR, Negera WG, Molla HT. Enhanced Region Growing for Brain Tumor MR Image Segmentation. Journal of Imaging. 2021 Feb;7(2):22.
174. Rai HM, Chatterjee K, Dashkevich S. Automatic and accurate abnormality detection from brain MR images using a novel hybrid UnetResNext-50 deep CNN model. Biomedical Signal Processing and Control. 2021 Apr 1;66:102477.
175. Mitchell JR, Kamnitsas K, Singleton KW, Whitmire SA, Clark-Swanson KR, Ranjbar S, Rickertsen CR, Johnston SK, Egan KM, Rollison DE, Arrington J. Deep neural network to locate and segment brain tumors outperformed the expert technicians who created the training data. Journal of Medical Imaging. 2020 Oct;7(5):055501.
176. Abirami MS, Uma M, Gurumoorthy R, Narayan S, Hameed J. Brain Tumor Segmentation in MRI Images Using UNet based 3D CNN. Annals of the Romanian Society for Cell Biology. 2021 Mar 20:325-35.
177. AL-Dabagh MZ. Automated tumor segmentation in MR brain image using fuzzy c-means clustering and seeded region methodology. Int J Artif Intell ISSN.;2252(8938):8938.
178. Sohail N, Anwar SM, Majeed F, Sanin C, Szczerbicki E. Smart Approach for Glioma Segmentation in Magnetic Resonance Imaging using Modified Convolutional Network Architecture (U-NET). Cybernetics and Systems. 2020 Dec 31:1-6.
179. Sran PK, Gupta S, Singh S. Integrating saliency with fuzzy thresholding for brain tumor extraction in MR images. Journal of Visual Communication and Image Representation. 2021 Jan 1;74:102964.
180. Saxena S, Kumari N, Pattnaik S. Brain Tumour Segmentation in FLAIR MRI Using Sliding Window Texture Feature Extraction Followed by Fuzzy C-Means Clustering. International Journal of Healthcare Information Systems and Informatics (IJHISI). 2021 Jul 1;16(3):1-20.
181. Takahashi S, Takahashi M, Kinoshita M, Miyake M, Kawaguchi R, Shinojima N, Mukasa A, Saito K, Nagane M, Otani R, Higuchi F. Fine-Tuning Approach for Segmentation of Gliomas in Brain Magnetic Resonance Images with a Machine Learning Method to Normalize Image Differences among Facilities. Cancers. 2021 Jan;13(6):1415.
182. Latif U, Shahid AR, Raza B, Ziauddin S, Khan MA. An end‐to‐end brain tumor segmentation system using multi‐inception‐UNET. International Journal of Imaging Systems and Technology. 2021 Apr 19.
183. Zhang W, Yang G, Huang H, Yang W, Xu X, Liu Y, Lai X. ME‐Net: Multi‐encoder net framework for brain tumor segmentation. International Journal of Imaging Systems and Technology. 2021 Mar 7.
184. Lei X, Yu X, Chi J, Wang Y, Zhang J, Wu C. Brain tumor segmentation in MR images using a sparse constrained level set algorithm. Expert Systems With Applications. 2021 Apr 15;168:114262.
185. Wang Y, Peng J, Jia Z. Brain tumor segmentation via C-dense convolutional neural network. Progress in Artificial Intelligence. 2021 Jun;10(2):147-56.
186. Zhao Y, Ren X, Hou K, Li W. Recurrent Multi-Fiber Network for 3D MRI Brain Tumor Segmentation. Symmetry. 2021 Feb;13(2):320.
187. Cao Y, Vassantachart A, Jason CY, Yu C, Ruan D, Sheng K, Lao Y, Shen ZL, Balik S, Bian S, Zada G. Automatic detection and segmentation of multiple brain metastases on magnetic resonance image using asymmetric UNet architecture. Physics in Medicine & Biology. 2021 Jan 8;66(1):015003.
188. Barzegar Z, Jamzad M. WLFS: Weighted label fusion learning framework for glioma tumor segmentation in brain MRI. Biomedical Signal Processing and Control. 2021 Jul 1;68:102617.
189. Jayachandran A, Dhanasekaran R. Automatic detection of brain tumor in magnetic resonance images using multi‐texton histogram and support vector machine. International Journal of Imaging Systems and Technology. 2013 Jun;23(2):97-103.
190. Farjam R, Parmar HA, Noll DC, Tsien CI, Cao Y. An approach for computer-aided detection of brain metastases in post-Gd T1-W MRI. Magnetic resonance imaging. 2012 Jul 1;30(6):824-36.
191. Jayachandran A, Dhanasekaran R. Brain tumor severity analysis using modified multi‐texton histogram and hybrid kernel SVM. International journal of imaging systems and technology. 2014 Mar;24(1):72-82.
192. Dvořák P, Kropatsch WG, Bartušek K. Automatic brain tumor detection in t2-weighted magnetic resonance images. Measurement Science Review. 2013 Sep 1;13(5):223-30.
193. El-Dahshan ES, Mohsen HM, Revett K, Salem AB. Computer-aided diagnosis of human brain tumor through MRI: A survey and a new algorithm. Expert systems with Applications. 2014 Sep 1;41(11):5526-45.
194. Helen R, Kamaraj N. CAD scheme to detect brain tumour in MR images using active contour models and tree classifiers. Journal of Electrical Engineering and Technology. 2015;10(2):670-5.
195. Thirumurugan P, Ramkumar D, Batri K, Siva Sundhara Raja D. Automated detection of glioblastoma tumor in brain magnetic imaging using ANFIS classifier. International Journal of Imaging Systems and Technology. 2016 Jun;26(2):151-6.
196. Banerjee S, Mitra S, Shankar BU, Hayashi Y. A novel GBM saliency detection model using multi-channel MRI. PloS one. 2016 Jan 11;11(1):e0146388.
197. Amin J, Sharif M, Yasmin M, Fernandes SL. A distinctive approach in brain tumor detection and classification using MRI. Pattern Recognition Letters. 2017 Nov 1;139:118-27.
198. Anitha R, Siva Sundhara Raja D. Development of computer‐aided approach for brain tumor detection using random forest classifier. International Journal of Imaging Systems and Technology. 2018 Mar;28(1):48-53.
199. Lahmiri S. Glioma detection based on multi-fractal features of segmented brain MRI by particle swarm optimization techniques. Biomedical Signal Processing and Control. 2017 Jan 1;31:148-55.
200. Deepa AR, Emmanuel WS. An efficient detection of brain tumor using fused feature adaptive firefly backpropagation neural network. Multimedia Tools and Applications. 2019 May;78(9):11799-814.
201. Selvapandian A, Manivannan K. Performance analysis of meningioma brain tumor classifications based on gradient boosting classifier. International Journal of Imaging Systems and Technology. 2018 Dec;28(4):295-301.
202. Arunkumar N, Mohammed MA, Mostafa SA, Ibrahim DA, Rodrigues JJ, de Albuquerque VH. Fully automatic model‐based segmentation and classification approach for MRI brain tumor using artificial neural networks. Concurrency and Computation: Practice and Experience. 2020 Jan 10;32(1):e4962.
203. Edalati-rad A, Mosleh M. Improving Brain Tumor Diagnosis Using MRI Segmentation Based on Collaboration of Beta Mixture Model and Learning Automata. Arabian Journal for Science and Engineering. 2019 Apr;44(4):2945-57.
204. Song G, Huang Z, Zhao Y, Zhao X, Liu Y, Bao M, Han J, Li P. A noninvasive system for the automatic detection of gliomas based on hybrid features and PSO-KSVM. IEEE Access. 2019 Jan 31;7:13842-55.
205. Johnpeter JH, Ponnuchamy T. Computer aided automated detection and classification of brain tumors using CANFIS classification method. International Journal of Imaging Systems and Technology. 2019 Dec;29(4):431-8.
206. Alam MS, Rahman MM, Hossain MA, Islam MK, Ahmed KM, Ahmed KT, Singh BC, Miah MS. Automatic human brain tumor detection in MRI image using template-based K means and improved fuzzy C means clustering algorithm. Big Data and Cognitive Computing. 2019 Jun;3(2):27.
207. Atici MA, Sagiroglu S, Celtikci P, Ucar M, Borcek AO, Emmez H, Celtikci E. A novel deep learning algorithm for the automatic detection of high-grade gliomas on T2-weighted magnetic resonance images: A preliminary machine learning study. Turkish neurosurgery. 2020 Jan 1;30(2):199-205.
208. Çinar A, Yildirim M. Detection of tumors on brain MRI images using the hybrid convolutional neural network architecture. Medical hypotheses. 2020 Jun 1;139:109684.
209. Devanathan B, Venkatachalapathy K. Brain Tumor Detection and Classification Model Using Optimal Kapur’s Thresholding Based Segmentation with Deep Neural Networks. IIOABJ. 2020 Jul;11(4);1-8
210. Dikici E, Ryu JL, Demirer M, Bigelow M, White RD, Slone W, Erdal BS, Prevedello LM. Automated brain metastases detection framework for T1-weighted contrast-enhanced 3D MRI. IEEE journal of biomedical and health informatics. 2020 Mar 23;24(10):2883-93.
211. Dheepa G, Chithra PL. A Fully-Automated Detection of Brain Tumor in MRI Images using Input Cascaded CNN. Computer Science. 2020;15(4):1193-7.
212. Gurunathan A, Krishnan B. Detection and diagnosis of brain tumors using deep learning convolutional neural networks. International Journal of Imaging Systems and Technology. 2020 Dec 16.
213. Wang J, Shao W, Kim J. Automated classification for brain MRIs based on 2D MF-DFA method. Fractals. 2020 Sep 18;28(06):2050109.
214. Kesav OH, Rajini GK. Automated detection system for texture feature based classification on different image datasets using S-transform. International Journal of Speech Technology. 2021 Jun;24(2):251-8.
215. Murali E, Meena K. Brain Tumor Detection from MRI using Adaptive Thresholding and Histogram based Techniques. Scalable Computing: Practice and Experience. 2020 Mar 19;21(1):3-10.
216. Kaur T, Gandhi TK. Deep convolutional neural networks with transfer learning for automated brain image classification. Machine Vision and Applications. 2020 Mar;31(3):1-6.
217. Thangarajan SK, Chokkalingam A. Integration of optimized neural network and convolutional neural network for automated brain tumor detection. Sensor Review. 2021 Feb 8.
218. Kalaiselvi T, Padmapriya T, Sriramakrishnan P, Priyadharshini V. Development of automatic glioma brain tumor detection system using deep convolutional neural networks. International Journal of Imaging Systems and Technology. 2020 Dec;30(4):926-38.
219. Rajinikanth V, Joseph Raj AN, Thanaraj KP, Naik GR. A customized VGG19 network with concatenation of deep and handcrafted features for brain tumor detection. Applied Sciences. 2020 Jan;10(10):3429.
220. Huang Z, Xu H, Su S, Wang T, Luo Y, Zhao X, Liu Y, Song G, Zhao Y. A computer-aided diagnosis system for brain magnetic resonance imaging images using a novel differential feature neural network. Computers in biology and medicine. 2020 Jun 1;121:103818.
221. Chen B, Zhang L, Chen H, Liang K, Chen X. A novel extended Kalman filter with support vector machine based method for the automatic diagnosis and segmentation of brain tumors. Computer Methods and Programs in Biomedicine. 2021 Mar 1;200:105797.
222. Patil DO, Hamde ST. Automated detection of brain tumor disease using empirical wavelet transform based LBP variants and ant-lion optimization. Multimedia Tools and Applications. 2021 May;80(12):17955-82.
223. Simaiya S, Lilhore UK, Prasad D, Verma DK. MRI Brain Tumour Detection & Image Segmentation by Hybrid Hierarchical K-means clustering with FCM based Machine Learning Model. Annals of the Romanian Society for Cell Biology. 2021 Jan 28:88-94.
224. Tejas P. A novel hybrid approach to detect brain tumor in MRI images. Turkish Journal of Computer and Mathematics Education (TURCOMAT). 2021 Apr 11;12(3):3412-6.
